# Autoimmune PaneLs as PrEdictors of Toxicity in Patients TReated with Immune Checkpoint InhibiTors (ALERT)

**DOI:** 10.1186/s13046-023-02851-6

**Published:** 2023-10-21

**Authors:** Sofia Genta, Katherine Lajkosz, Noelle R. Yee, Pavlina Spiliopoulou, Alya Heirali, Aaron R. Hansen, Lillian L. Siu, Sam Saibil, Lee-Anne Stayner, Maryia Yanekina, Maxwell B. Sauder, Sareh Keshavarzi, Abdulazeez Salawu, Olga Vornicova, Marcus O. Butler, Philippe L. Bedard, Albiruni R. Abdul Razak, Robert Rottapel, Andrzej Chruscinski, Bryan Coburn, Anna Spreafico

**Affiliations:** 1 Division of Medical Oncology and Hematology, Princess Margaret Cancer Centre, University Health Network, University of Toronto, Toronto, ON Canada; 2 Department of Biostatistics, Princess Margaret Cancer Centre, Toronto, ON Canada; 3 Toronto General Research Institute, University Health Network Toronto, Toronto, ON Canada; 4 Division of Dematology, Department of Medicine, University of Toronto, Toronto, ON Canada; 5 Department of Immunology, University of Toronto, Toronto, ON Canada; 6 University Health Network, University of Toronto, Toronto, ON Canada

## Abstract

**Background:**

Immune-checkpoint inhibitors (ICI) can lead to immune-related adverse events (irAEs) in a significant proportion of patients. The mechanisms underlying irAEs development are mostly unknown and might involve multiple immune effectors, such as T cells, B cells and autoantibodies (AutoAb).

**Methods:**

We used custom autoantigen (AutoAg) microarrays to profile AutoAb related to irAEs in patients receiving ICI. Plasma was collected before and after ICI from cancer patients participating in two clinical trials (NCT03686202, NCT02644369). A one-time collection was obtained from healthy controls for comparison. Custom arrays with 162 autoAg were used to detect IgG and IgM reactivities. Differences of median fluorescent intensity (MFI) were analyzed with Wilcoxon sign rank test and Kruskal–Wallis test. MFI 500 was used as threshold to define autoAb reactivity.

**Results:**

A total of 114 patients and 14 healthy controls were included in this study. irAEs of grade (G) ≥ 2 occurred in 37/114 patients (32%). We observed a greater number of IgG and IgM reactivities in pre-ICI collections from patients versus healthy controls (62 vs 32 *p* < *0.001*). Patients experiencing irAEs G ≥ 2 demonstrated pre-ICI IgG reactivity to a greater number of AutoAg than patients who did not develop irAEs (39 vs 33 *p* = *0.040)*. We observed post-treatment increase of IgM reactivities in subjects experiencing irAEs G ≥ 2 (29 vs 35, *p* = *0.021*) and a decrease of IgG levels after steroids (38 vs 28, *p* = *0.009)*.

**Conclusions:**

Overall, these results support the potential role of autoAb in irAEs etiology and evolution. A prospective study is ongoing to validate our findings (NCT04107311).

**Supplementary Information:**

The online version contains supplementary material available at 10.1186/s13046-023-02851-6.

## Background

Immune checkpoint inhibitors (ICI) targeting the PD1/PD-L1 and CTLA4 pathways have rapidly become one of the main treatment option for cancer patients [1]. In a significant proportion of recipients, ICI can lead to life-threatening or disabling immune-related adverse events (irAEs) [2–4]. As compared to side effects from cytotoxic treatments, the type, severity and the timing of irAEs is less predictable [5]. The majority of irAEs occur within the first few months of treatment [6]. However, it is not uncommon to observe delayed and long lasting toxicities that fluctuate over time [7]. Although some organs such as skin, thyroid and the gastrointestinal tract are more frequently involved, all body systems can be affected by immune-mediated toxicity [6]. While endocrine toxicities can be managed with hormone replacement, irAEs involving other organs often require immunosuppressive therapies, ranging from systemic steroids to biological agents [7]. Importantly, irAEs can result in death in up to 1.3% of patients [7]. A deeper understanding the etiology of irAEs and the identification of reliable predictive biomarkers of toxicity are unmet clinical needs to appropriately implement novel therapies and reduce morbidity in cancer patients [8, 9].

The correlation between clinical benefit from ICI and immune-mediated toxicities may be linked to T cell activation [10–15]. The observation of organ-infiltrating T-cell during irAEs including myocarditis [16], colitis [17], nephritis [18], pneumonitis [19] and sicca syndrome [20] supports this hypothesis. The antitumor activity of ICI however, does not solely rely on T lymphocytes and can be deeply influenced by other elements of the tumor microenvironment [21]. Similarly, irAEs might be the result of a complex network of interactions between tumors, healthy cells and multiple immune system effectors [5, 22–24]. Several reports indicate that ICI-induced de-regulation of B-cells and altered production of autoantibodies (AutoAb) may contribute to the onset of irAEs [25–34]. The ability to detect AutoAb in patients at risk to develop irAEs even prior ICI exposure has been explored in multiple studies [26, 28, 35–41]. While several studies support the use of pre-ICI anti-thyroid antibodies to predict the risk of thyroiditis [42, 43], no clear correlation has been identified between other types of tissue specific AutoAb and organ toxicity.

Our group has developed a large, customized panel for AutoAb profiling to enable the simultaneous detection of reactivity against multiple tissue-specific antigens [44]. In the current study, we used the customized AutoAb array to interrogate the levels of AutoAb at baseline and throughout the course of ICI therapy in cancer patients who developed irAEs versus patients who did not experience immune-mediated toxicities.

## Methods

### Study population

Key eligibility criteria for this study included a diagnosis of solid tumor and history of treatment with an anti-PD1 based regimen. We identified a cohort of 114 patients with metastatic solid cancers who received treatment with ICI in two clinical trials (INSPIRE, NCT02644369 and MET4-IO, NCT03686202) [45]. INSPIRE was a phase 2 study investigating novel immune biomarkers in patients with solid tumors receiving pembrolizumab [46, 47] while MET4-IO evaluated an orally-delivered microbiome intervention in cancer patients receiving ICI [48, 49]. Both were investigator-initiated studies, approved by the Princess Margaret Cancer Centre Research Ethics Board (#18–5950 and 15–9828). Samples obtained from the participants were used for this correlative study without interfering with the primary objectives of the two clinical trials. Plasma samples obtained from patients before and after ICI were retrospectively analyzed. Collection time points in the MET4-IO study included baseline (within 14 days before the start of ICI), 3–4 weeks, 6–8 weeks, 24 weeks and at the end of treatment, while time-points for the patients enrolled in the INSPIRE trial included baseline (within 10 days from the start of ICI) and 6 weeks (cycle 3). Plasma samples collected from 14 healthy controls at a single time point were used for comparison. Treatment emergent adverse events with potential immune related etiology were prospectively recorded for all the patients and graded according to the Common Terminology Criteria for Adverse Events (CTCAE), version 5.0. For the purpose of this study, we considered irAEs of grade (G) ≥ 2 requiring medical intervention including steroids or other immunosuppressive agents or hormone replacement. Information regarding pharmacological interventions for the treatment of irAEs, including the type, dose and duration of treatment were collected for all the participants.

### AutoAb detection and measurement with antigen microarray

A total of 162 antigens, customized based on tissues or organs most frequently affected by irAEs were selected (a list of the antigens used for this study is provided in Supplementary Table 1). The antigens included in the panel are associated with multiple autoimmune disorders and have been used to screen for autoantibodies in a variety of conditions such as heart failure/heart transplantation [44, 50], kidney transplantation [51], liver transplantation [52], lung transplantation [53], systemic autoimmune rheumatic diseases [54], and post-covid vaccination [55]. Antigens were diluted in PBS to 0.2 mg/ml and subsequently stored at -80 °C. Customized antigen microarrays were generated as described previously [56, 57]. Briefly, antigens were spotted in duplicate onto nitrocellulose coated slides (GVS, Sanford, ME, USA) using a VersArray Chipwriter Pro (Virtek, Waterloo, Canada) with solid pins (Arrayit, Sunnyvale, CA, USA). Slides were blocked overnight in blocking buffer (PBS with 0.1% Tween and 5% FBS). The slides were then probed with human serum (diluted 1:100) in blocking buffer for one hour at 4 °C. After washing, the slides were probed with a Cy3-labelled goat anti-human IgG Fc antibody (Jackson ImmunoResearch, Westgrove, PA, USA) and an Alexa Fluor 647-labelled goat anti-human IgM antibody (Jackson ImmunoResearch) for 45 min at 4 °C in blocking buffer. After additional washing, the slides were dried by centrifugation. Fluorescence was quantified using an Axon 4200A scanner (Molecular Devices, Sunnyvale, CA). Median fluorescence intensity (MFI) on both the IgG and IgM channels was calculated for each antigen by subtracting the local background fluorescence and then averaging duplicate features.

### AutoAb detection and measurement by enzyme-linked immunosorbent assay (ELISA)

The concentrations of IgM and IgG antibodies in plasma samples were measured by commercial ELISA kits (Invitrogen; Thermo Fisher Scientific, Inc., Waltham, MA, USA) according to manufacturer protocol. Briefly, 96-well plates were coated with anti-human IgM or IgG monoclonal antibodies overnight at 4 °C. After blocking, the plates were incubated with plasma samples (diluted 10,000-fold and 500,000-fold for IgM and IgG assays, respectively) and appropriate standards at room temperature for 2 h, followed by incubation with HRP-conjugated antibody at room temperature for 1 h. Tetramethylbenzidine substrate solution was then added to each well to incubate in the dark for 15 min at room temperature before the addition of stop solution (2N H2SO4). All samples were run in duplicate. Finally, absorbance at 450 nm was detected by a plate reader to calculate IgM and IgG concentrations via standard curve OD values.

### Statistical analysis

Mann–Whitney U test and Fisher’s exact test or the Chi-squared test were used to compare continuous and categorical variables, respectively, across patients with and without irAEs G ≥ 2. The assessment of differences in IgG and IgM distribution incorporated the Benjamini–Hochberg correction for multiple comparisons. Receiver operating characteristics (ROC) analysis was used to determine the optimal cutoff for IgG and IgM MFI to differentiate patients with and without irAEs G ≥ 2. The optimal cutoff was selected using the Youden index. AutoAb with MFI greater than the optimal cutoff per individual, as well as greater than 500, were compared between healthy controls and patients with and without irAEs G ≥ 2 using the Kruskal–Wallis test. Amongst patients with irAEs G ≥ 2, change in MFI > optimal cutoff from screening to toxicity, and change from toxicity to steroid use were modelled using the Wilcoxon signed-rank test. The analysis was repeated with patients without irAEs to evaluate change from screening to next collection. To assess cumulative incidence of irAEs G ≥ 2 by MFI group, time to irAEs G ≥ 2 was calculated as the number of months between first cycle and date of irAEs G ≥ 2. Patients without irAEs G ≥ 2 were censored at date of last follow up. Death was considered a competing risk. For each group, patients were categorized into “high” vs. “low” groups based on the median number of AutoAb above the selected cutoff. Differences in the cumulative incidence by group were assessed using Gray’s test. Patients were stratified by age group (≤ 45 vs. > 45, ≤ 50 vs. > 50 and ≤ 60 vs. 60 years) and differences in AutoAb distribution were assessed using the Mann–Whitney U test.

## Results

### Patients’ characteristics

A total of 14 healthy controls and 114 cancer patients were included in this analysis. Clinical and treatment characteristics of the study population are described in Table 1. The median age of the healthy controls was 35 years, and the majority were female (64%). The median age of the patients was 61 years and 51% were male. Thirty-seven patients (32%) developed irAEs G ≥ 2. The type of therapy received (anti-PD1 with or without anti-CTLA4 antibodies), the type of tumor, the number of prior lines and the sex of the patients were significantly associated with the risk of developing irAEs. A greater proportion of irAEs G ≥ 2 was observed in patients receiving anti-PD1 in combination with anti-CTLA4 agents (24% vs 4%, *p* = *0.0018*). As compared with other tumor types, patients with melanoma had a higher proportion of irAEs G ≥ 2 (43% vs 12%, *p* = *0.022*) and greater proportion of irAEs G ≥ 2 occurred in patients who received immunotherapy as their first line of anticancer treatment as compared to those who had one or more prior lines of treatment (51% vs 18%, *p* = *0.012*). A higher risk of irAEs G ≥ 2 was observed in male vs female (41% vs 23%, *p* = *0.017*). The association between tumor type, number of prior lines and sex of the patients and the probability to develop irAEs was confirmed after adjusting for the type of treatment received. Patients who experienced irAEs G ≥ 2 were older; however, this difference was not significant after adjusting for treatment type (65 vs 59 years, *p* = *0.07*). Eight patients had a past history of autoimmune disorders (3 hypothyroidism, 1 Hashimoto syndrome, 1 psoriasis, 1 rheumatoid arthritis, 1 diabetes mellitus, 1 Schoenlein-Henoch purpura). No significant association between prior autoimmune conditions and development of irAEs G ≥ 2 was observed in this cohort (*p* = *0.90*). We observed 18 different types of irAEs (Supplementary Fig. 1). The most frequently observed were hypothyroidism (13 patients, 11%), pneumonitis (10 patients, 9%), colitis/diarrhea (7 patients, 6%), skin toxicity (7 patients, 6%), hepatitis (5 patients, 4%), infusion reaction (2 patients, 2%), myocarditis/troponin increase (2 patients, 2%) and pancreatitis/lipase increase (2 patients, 2%). In 4/13 cases (31%) hypothyroidism was preceded by subclinical hyperthyroidism detected with routine blood tests. Nineteen patients (17%) developed more than one irAE G ≥ 2.

**Table 1:** Demographic and treatment characteristics

*Covariate*	*All patients*	*Patients without irAEs G* ≥ *2*	*Patients with irAES G* ≥ *2*	*Healthy Controls*	p-value*
**Total**	114	77	37	14	
**Median Age**	61 (21–81)	59 (21–81)	65 (24–81)	31 (18–58)	0.070
**Sex**					
Male	58 (51)	34 (44)	24 (65)	5 (36)	**0.017**
Female	56 (49)	43 (56)	13 (35)	9 (64)	
**History of prior autoimmune disease**					
Yes	8 (7)	5 (6)	3 (8)	NA	0.90
No	106 (93)	72 (94)	34 (92)	NA	
**Type of immunotherapy**					
Anti-PD1	102 (89)	74 (96)	28 (76)	NA	
Anti-PD1 + Anti-CTLA4	12 (11)	3 (4)	9 (24)	NA	
**Tumor type**					**0.022**
HNSCC	33 (29)	20 (26)	13 (35)	NA	
Melanoma	25 (22)	9 (12)	16 (43)	NA	
TNBC	22 (19)	21 (27)	1 (3)	NA	
Others	34 (30)	27 (35)	7 (19)	NA	
**N of prior lines**					**0.012**
0	33 (29)	13 (17)	19 (51)	NA	
1	38 (33)	26 (34)	14 (38)	NA	
2	25 (22)	20 (26)	4 (11)	NA	
≥ 3	18 (16)	18 (24)	0 (0)	NA	
**Prior Immunotherapy**					0.42
No	109 (96)	74 (96)	34 (92)	NA	
Yes	5 (4)	3 (4)	3 (8)	NA	
**Study**					**0.039**
INSPIRE	84 (74)	65 (84)	19 (51)	NA	
MET4	30 (26)	12 (16)	18 (49)	NA	

*HNSCC* head and neck squamous cell cancer, *irAEs* immune-related adverse events, *NA* not applicable, *TNBC* triple negative breast cancer

^*^P-values from Cochran-Mantel–Haenszel test stratified by monotherapy (anti-PD1 alone) vs. combination therapy (anti-PD1 + anti-CTLA4) for categorical characteristics, and from logistic regression models adjusting for mono vs. combination therapy for continuous characteristics

### Cancer patients had a greater number of IgG and IgM reactivities before ICI administration than healthy controls

**Fig. 1:**
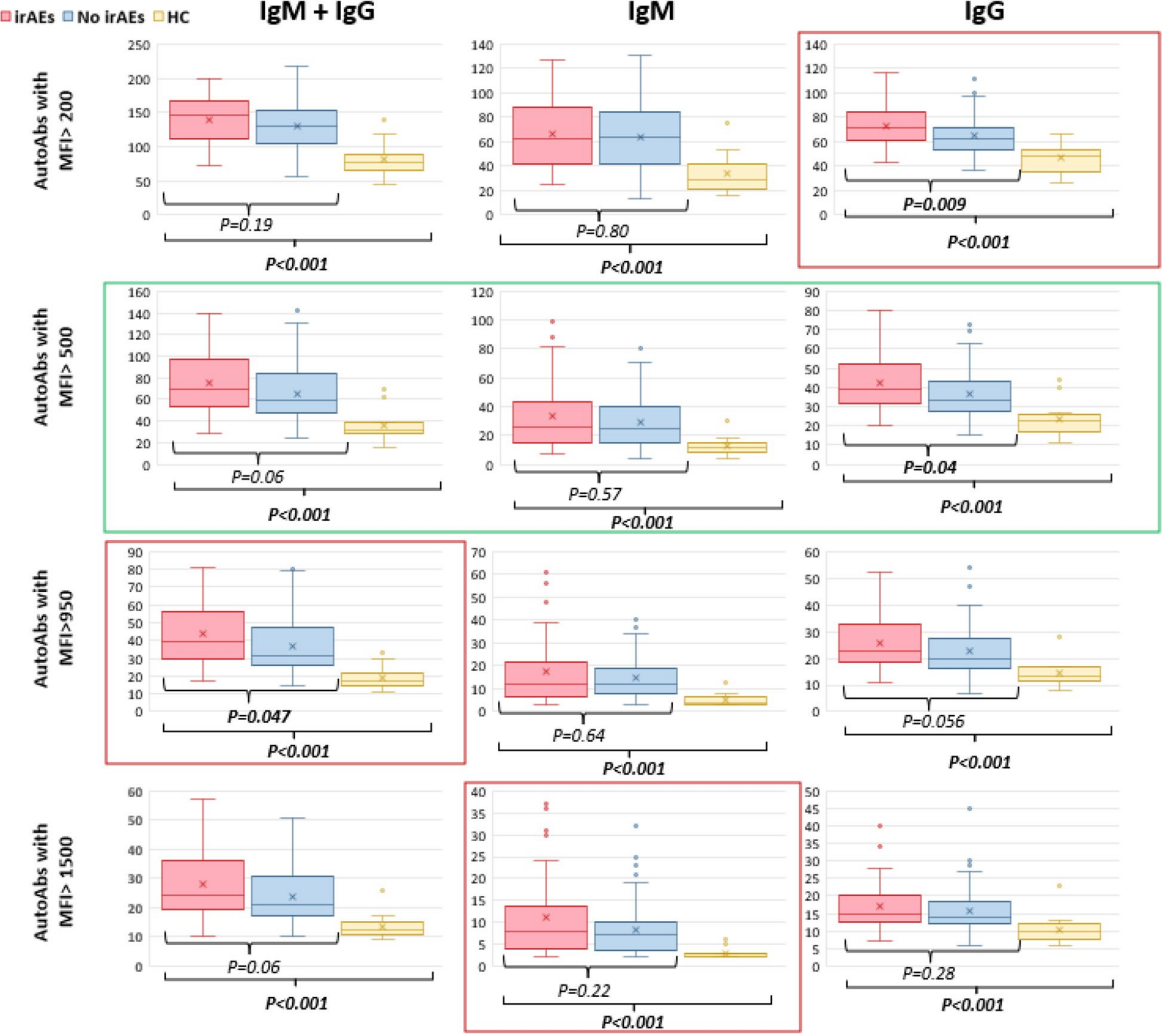
Comparison of AutoAbs in pre-ICI collections from patients with and without irAEs G ≥ 2 and in healthy controls using different cut-offs. A cut-off of MFI 500 (circled in green) was initially selected as this represent the value at which we expect to observe reactivity with the ELISA test. Optimal cut-offs identified with ROC analysis for different classes of AutoAbs are circled in red

Autoimmune reactivity between patients with and without irAEs G ≥ 2 and healthy controls was compared using an MFI > 500 as a threshold. This level was previously identified as the threshold for signal identification by ELISA [44]. We evaluated both IgG and IgM AutoAb with MFI > or ≤ 500 in plasma samples collected from 114 cancer patients before ICI administration and from 14 healthy controls and assessed differences using the Mann–Whitney U test. A statistically significant difference in AutoAb reactivities with MFI > 500 was observed in samples collected from the healthy controls (median 32, [IQR 29–37.5]) versus AutoAb with MFI > 500 samples collected from cancer patients before the start of immunotherapy (median 62; [IQR 50–90]; *p* < *0.001*). The median number of IgM reactivities in samples from healthy controls was 11.5 (IQR 9–14) while in cancer patients was 25 (IQR 15–41; *p* < *0.001*). The median number of IgG reactivities in healthy controls was 23 (IQR 18–26) while in cancer patients was 34.5 (IQR 29–45) (*p* < *0.001*) (Fig. 1). The healthy control’s median age was lower as compared to patients (31 vs 61 years, see Table 1). To evaluate the potential impact of this variable we divided the patients into two groups according to their age and compared IgG and IgM reactivities before ICI exposure in the two groups. Multiple thresholds were explored. The only significant difference observed was between patients ≤ 50 years vs > 50 years. Using this threshold, a lower median number of IgM and IgG with MFI > 500 was observed in the elderly population (60 vs 73, *p* = *0.04*, see Supplementary Table 2).

###  Patients who develop G ≥ 2 irAEs have higher number of AutoAb reactivities at baseline

We compared the number of IgG and IgM reactivities with MFI > 500 in plasma collected before ICI administration from patients who subsequently developed irAEs G ≥ 2 versus those who did not using the Mann–Whitney U test. Samples from 37/114 patients (32%), who developed irAEs and 77/114 patients (68%) who did not develop irAEs were available for this analysis. Patients who experienced irAEs G ≥ 2 had a median of 69 (IQR 54–96) autoAg reactivities in their pre-ICI collection while the median number in patients who did not develop irAEs G ≥ 2 was 59 (IQR 49–84) (*p* = *0.06*). We observed a statistically significantly higher number of reactive IgG in plasma collected from patients who developed irAEs (median 39, IQR 32–52) than in samples from patients who did not develop irAEs G ≥ 2 (median 33, IQR 28–43, *p* = *0.04*). This difference was confirmed in the subgroup of patients who received anti-PD1 single agent (median 33 [IQR 27–42] vs 38 [IQR 32–53], *p* = *0.04*) but not in those who received the combination with anti-CTLA-4 (median 50 [IQR 40–52] vs 39 [IQR 33–44] *p* = *0.64*). No differences in the number of IgM reactivities were observed in plasma samples collected before the start of ICI from patients who did develop irAEs G ≥ 2 (median 26, IQR 16–43) and those who did not experience irAEs G ≥ 2 (median 25, IQR 15–40) (*p* = *0.57*).

Beside to using the a priori selected MFI threshold of 500, ROC analysis was performed to identify additional thresholds for IgG and IgM reactivity, to further explore whether there was a difference in pre-ICI IgM and IgG levels in patients with vs without irAEs G ≥ 2. Cut-offs ranging from 100 to 1500 (with interval width of 25) were considered. The selected cutoff for IgM and IgG, which maximized the differences between the two groups of patients, was found to be MFI 950. ROC analyses were conducted separately for IgG alone and IgM alone. The optimal cut-off for IgG alone was identified as MFI 200, and for IgM alone was identified as MFI 1500. Differences in elevated AutoAb between healthy controls and cancer patients with and without irAEs G ≥ 2 using different thresholds are reported in Fig. 1.

### Patients with a greater number of IgG reactivities at baseline are at higher risk to develop irAEs

**Fig. 2:**
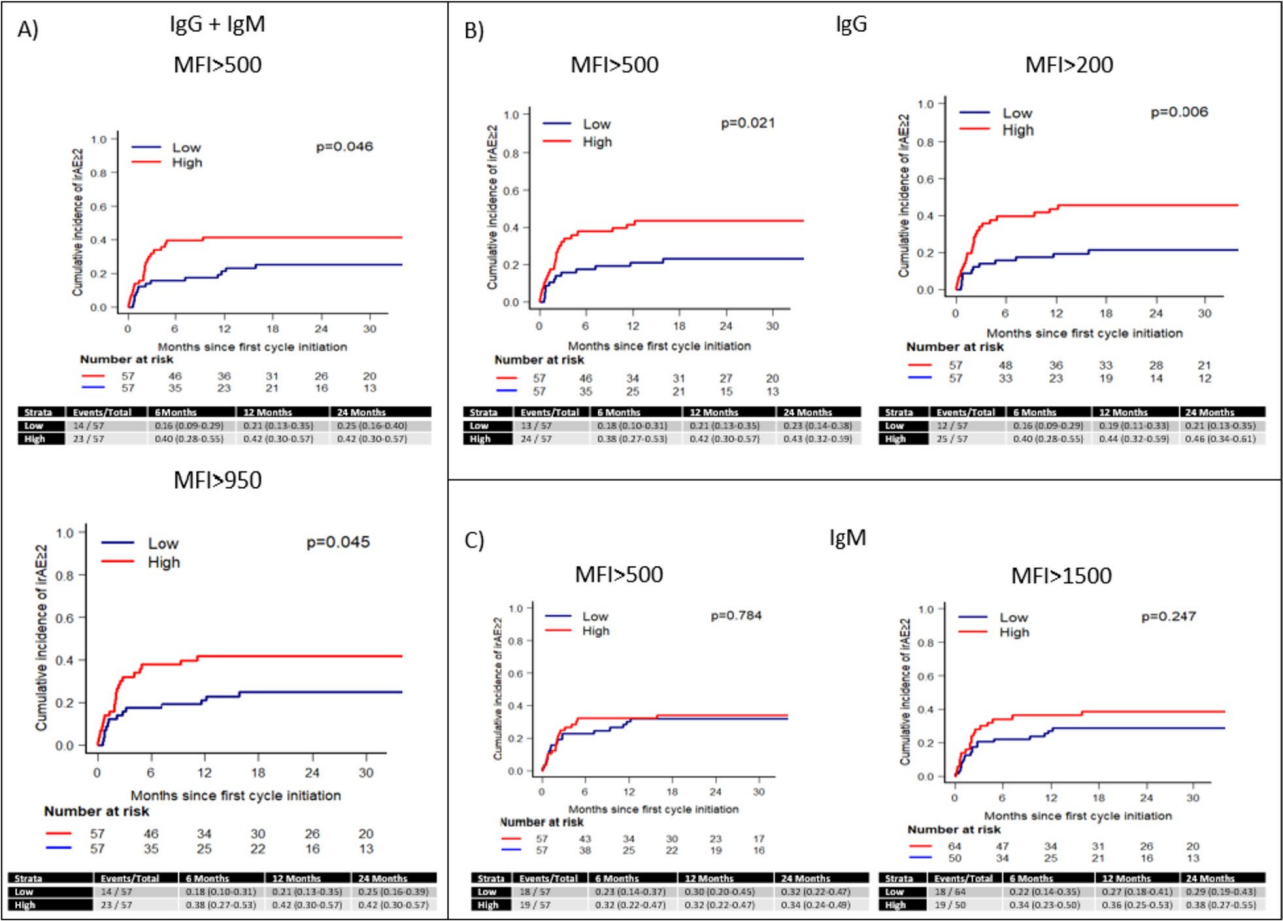
Cumulative incidence of irAEs in patients with “high” vs “low” autoAb at baseline using different cut-offs. **A** Cumulative incidence of irAEs in patients with “high” vs “low” levels of elevated IgG and IgM at baseline using MFI 500 and 950 as cut-offs. **B** Cumulative incidence of irAEs in patients with “high” vs “low” levels of elevated IgG at baseline using MFI 500 and 200 as cut-offs. **C** Cumulative incidence of irAEs in patients with “high” vs “low” levels of elevated IgM at baseline using MFI 500 and 1500 as cut-offs

We used the cut-offs identified in the prior analysis to divide the patients into “high” and “low” groups based on the number of AutoAb reactivities per patient detected in their pre-ICI collection. For total antibody reactivities as well as IgG reactivities at both thresholds, the “high” group had greater incidence of irAEs than the “low” group (Fig. 2A-B). No differences were observed in patients with “high” vs “low” IgM reactivity using MFI 500 or 1500 as cut-off (Fig. 2C).

### Patients who develop irAEs G ≥ 2 have an increase of IgM reactivities after ICI

**Fig. 3:**
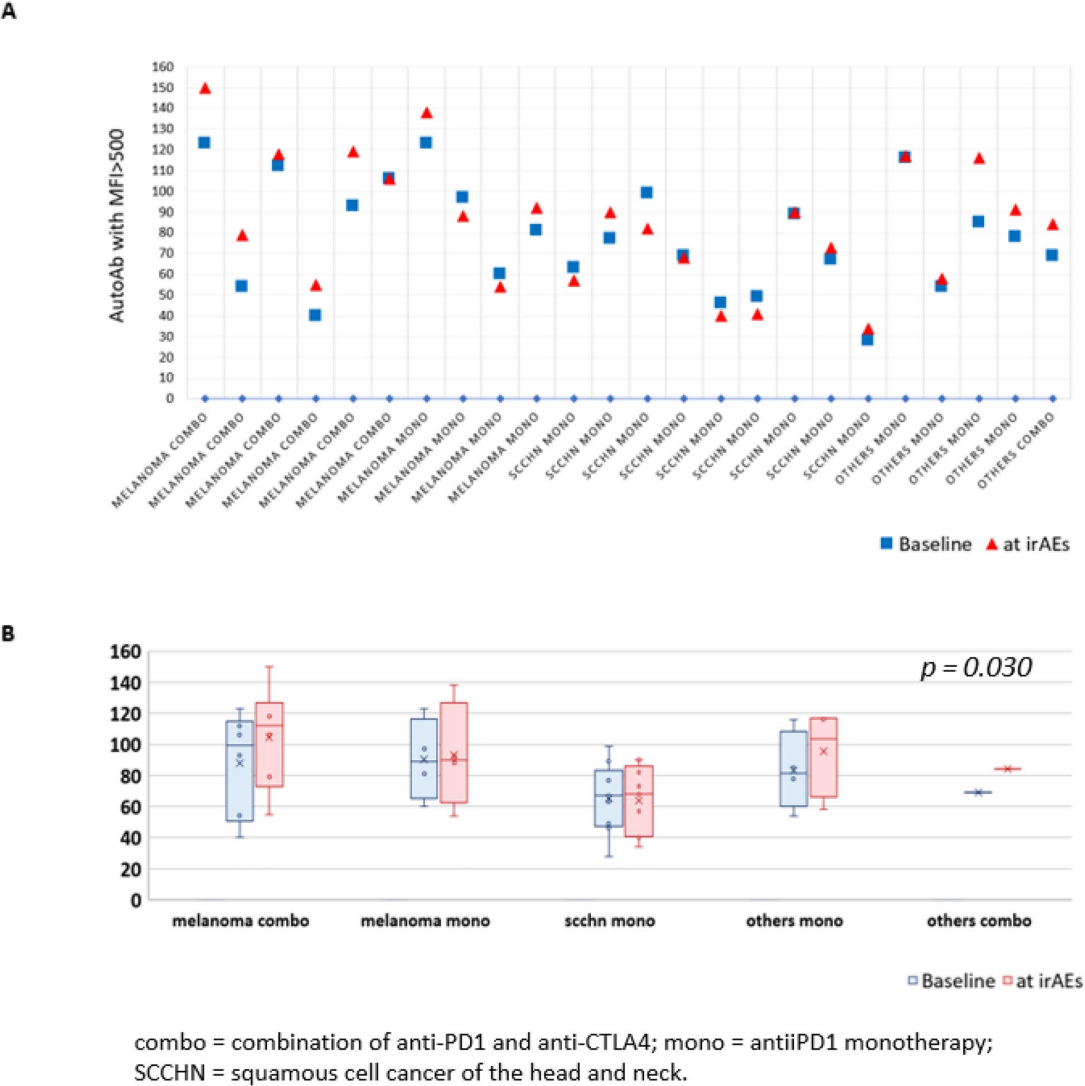
Comparison of the level of IgM and IgG with MFI > 500 before ICI administration (baseline) and within 8 weeks from the onset of irAEs in 24 patients. **A** The number of elevated autoAbs before ICI is represented as a blue square (one for each patient) while the number of elevate autoAbs at the time of the irAEs is represented as a red triangle. For each patient is reported the tumor type and the type of treatment received (monotherapy vs combination). **B** The differences in the number of IgM and IgG with MFI > 500 are reported for different group of patients divided based on treatment received and tumor types. While a significant increase was observed in melanoma patients receiving anti-PD1 + anti-CTLA4, no significant difference was observed in the other subgroups

Twenty-four of the 37 patients who developed irAEs (65%) had available plasma samples collected after ICI administration, within 8 weeks from the development of irAEs (median 21 days, range from -13 to 55 days after irAEs diagnosis) and prior to the start of any immunosuppressive therapy. The Wilcoxon signed-rank test was used to evaluate changes in samples. Using MFI 500 as threshold, the median number of total AutoAg reactivities before ICI initiation in this group was 78 while a median of 86 reactivities were detected in the post-ICI collection (*p* = *0.030*) (Fig. 3). A significant increase in post-ICI samples was confirmed for IgM reactivities (median 35 vs 29, *p* = *0.021*) while the number of IgG reactivities did not change (median 39.5 vs 39, *p* = *0.17).* No significant changes were observed in the number of AutoAb reactivities pre- and post-ICI in patients who did not develop G ≥ 2 irAEs (median 60 vs 58, *p* = *0.12 *Supplementary Fig. 2). The absence of significant difference was confirmed for IgM (24 vs 23, *p* = *0.72*) while post-treatment samples had a higher level of IgG (median 37 vs 33, *p* = *0.006*). Differences in the level of AutoAg reactivities from baseline to the first post ICI collection or to the closest collection to irAEs development, separated by IgG and IgM are reported in Supplementary Fig. 3*.*

### Patients who receive immuno-suppressive medications have a decrease of the number of IgG reactivities

We evaluated changes in the number of AutoAg reactivities in patients who received systemic immune suppressive agents to treat irAEs G ≥ 2 using the Wilcoxon signed-rank test. Nine of the 24 patients (37.5%) with available plasma collection within 8 weeks from the onset of irAEs had also available samples obtained after the start of immunosuppressive treatment. We observed a decrease in reactivities after immune suppression (median number of IgG and IgM with MFI > 500 54 post vs 79 pre-immune suppression *p* = *0.097*). The changes were mainly related to a decrease of IgG (median number of IgG with MFI > 500 28 vs 38 pre-immune suppression, *p* = *0.033*) while the levels of IgM were overall stable (median number of IgM with MFI > 500 33 vs 35 pre-immune suppression, *p* = *0.40 *Supplementary Fig. 4).

### Patients who develop irAEs have different mean and median MFI levels of specific AutoAb at baseline (pre-ICI) as compared with those who do not develop irAEs

The mean and median MFI for each specific AutoAg at baseline in patients with and without G ≥ 2 irAEs is reported in the Supplementary Table 3. No significant difference was observed with multiple comparison. AutoAb with significant difference between patients with and without irAEs at univariate analysis are reported in Supplementary Table 4*.* Additionally, no significant differences in the median MFI for each specific AutoAg were observed between patients with and without history of autoimmune disorders (Supplementary Table 5). We did not observe a correlation between a specific toxicity and high reactivity against a certain AutoAg (Supplementary Table 6). However, in some patients who experienced organ-specific irAEs we observed pre-ICI high levels of AutoAb against the related tissue. For instance, we detected high levels of IgG anti cardiac myosin before ICI exposure in a patient who subsequently experienced immune related myocarditis and very high pre-ICI levels of IgG anti-desmin in a patient who experienced colitis. Baseline and post-ICI reactivity of organ-specific AutoAb in selected patients, compared with median values in healthy controls and in all the patients before ICI exposure are reported in Supplementary Table 7.

### No difference in the level of IgG and IgM measured by ELISA is observed between patients with and without irAEs

We compared the value of total IgM and IgG measured by ELISA in the pre- and post-ICI samples from patients with and without G ≥ 2 irAEs. No differences were observed at baseline between patients with and without irAEs G ≥ 2 (median IgM value 3.47 g/L vs 2.88 g/L, *p* = *0.10*; median IgG value 15.18 g/L vs 13.97 g/L *p* = *0.49*). No significant changes from baseline were observed at the time of irAEs (median IgM value pre-ICI 3.63 g/L vs 3.87 g/L post ICI, *p* = *0.64*; median IgG value pre-ICI 15.91 g/L vs 15.60 g/L post-ICI, *p* = *0.22*) or between the time of toxicity and after steroids administration (median IgM value post-ICI 10.70 g/L vs post-steroid 8.73 g/L *p* = *0.58*; median IgG value post-ICI 17.11 g/L vs post-steroid 17.33 g/L *p* = *0.33)* (Supplementary Fig. 5)*.*

## Discussion

The relationship between cancer and immune system is only partially understood. The identification of reliable factors to distinguish upfront subjects with a higher risk of developing irAEs could improve treatment outcomes. Our data indicate the potential role of antibody-mediated autoreactivity as a predictive marker and/or causal factor in irAEs.

The link between generic pre-ICI elevation of AutoAb and subsequent development of irAEs is controversial [25]. Most published studies assess on a limited number of AutoAb, including rheumatoid factor, anti-thyroid and antinuclear antibodies (ANA) [25, 28, 58–60]. Few case reports have indicated an overall elevation of total IgG in patients who developed severe irAEs from ICI, supporting the potential role of gammaglobulin in irAEs etiopathogenesis [61–63]. However, the autoantibody levels were tested only after the development of the toxicities. To our knowledge, this is the first study using a large, customized panel of AutoAg to test reactivity against multiple antigens before immunotherapy exposure. Interestingly we observed a correlation between high pre-treatment IgG reactivity and irAEs development in the subgroup of patients treated with anti-PD1 single agent but not in those who received combined treatment. This might be due to the sample size (only 12/114 patients received anti-PD1 + anti-CTLA4). Nevertheless, it is possible that other factors, independent from autoAbs, are predominant in driving irAEs from anti-CTLA4.

We did not observe a significant correlation between pre-ICI reactivity against a specific AutoAg and a particular irAE, such as pneumonitis or hepatitis. Different types of antibodies however can cause similar clinical manifestations. As an example, ANA, anti-smooth muscle antibodies, and anti-liver–kidney microsomal antibodies are only some of the autoAbs that can lead to autoimmune liver disease [64]. Of note we observed some isolated cases of patients who developed toxicity of a particular organ such as the heart or the gut and had extremely high levels of specific AutoAb pre-ICI (Supplementary Table 7). These observations can be hypothesis-generating for future mechanistic studies.

In our study, a significant increase of IgM was detected near the time of ICI toxicity in patients who developed irAEs G ≥ 2. IgM are the first type of antibody generated during immune activation [65], therefore the increase of this subclass of immunoglobulins at the time of inflammation is not unexpected. The decrease in AutoAg reactive antibodies with immunosuppression is consistent with the historical data on the use of corticosteroids leading to IgG depletion without affecting the levels of other immunoglobulins subtypes [66–69]. Further research is needed to clarify if hypogammaglobulinemia following immunosuppressive treatment can represent a useful biomarker to predict early recovery from ICI-toxicities and select patients for rapid prednisone tapering.

Neoplastic processes are known to stimulate the production of AutoAb through different mechanisms, including rapid cancer cell turnover, altered protein expression and chronic inflammation [70, 71]. The median IgM levels detected with ELISA in pre-ICI collection from cancer patients included in this study were above the normal range established for the healthy population [72]. Moreover, in our cohort, cancer patients had higher IgG and IgM reactivity as compared to healthy controls. The patients and the healthy controls were not matched and multiple factors such as age and sex might have contributed to this difference. Still, a connection between cancer and increased AutoAb production can not be excluded. Paraneoplastic syndromes are a well-known phenomenon where tumors lead to the production of AutoAb attacking different organs such as the nervous system [73]. It is possible that there is a proportion of patients who, while developing a certain degree of autoreactivity, do not develop clinical manifestations. Anti-cancer treatments such as immunotherapy, can alter the equilibrium between cancer and the immune system by triggering AutoAb production and autoimmune phenomena through several mechanisms (e.g. cross-reactivity between cancer and healthy tissue antigens) [8]. The involvement of tissue specific AutoAb has been demonstrated for some of the most lethal ICI-related toxicities, such as AutoAb against the acetylcholine receptor in myasthenia-like syndrome [74], and anti-HU AutoAb in immune-related encephalitis [75]. These events could represent the effect of immunotherapy on an immune system already altered by cancer and prone to autoreactivity.

To assess the potential effect of senescence on AutoAb levels we compared IgG and IgM reactivity at baseline in patients, grouping them by age. Interestingly we observed a higher IgG and IgM reactivity in patients ≤ 50 years and no other significant differences. These results suggest that the greater reactivity observed in patients versus healthy controls might be related to the cancer rather than to the older age.

In our study, we observed a greater proportion of male patients developing G ≥ 2 irAE; however, a clear correlation between demographic characteristics, such as age or sex and probability of toxicity from ICI has not been established in large randomized clinical trials [35, 76, 77]. The female sex is known to have a greater propensity to develop some autoimmune conditions such as systemic lupus erythematosus and rheumatoid arthritis due to the effect of estrogen and prolactin on the immune system [78]. ICI activate the immune system regardless of the presence of sex hormones. This can explain why there is no evidence of an increased risk of irAEs in the female sex.

We did not observe a correlation between pre-existent autoimmune disorders and irAEs development. The absence of an increased risk of irAEs in patients with a history of autoimmune disease has also been reported in other studies and prospective trials are ongoing to validate the safety of ICI in this population [79–82]. Moreover, we did not detect higher reactivity against a specific AutoAg in patients with prior autoimmune conditions. This is not unexpected, the decrease of AutoAbs titers overtime following the acute phase of an autoimmune disease is a common event [83, 84].

Our study has several limitations, including the heterogeneity of tumor types. The indications of approved ICI-based therapies however are constantly expanding. Therefore, a standardized test, able to identify upfront patients with a higher risk of irAEs, across different tumor types would be of great clinical value. We used a novel approach to measure global reactivity against a large number of antigens to assess the potential role of pre-existent AutoAg reactivity in irAEs development. Importantly this approach, going beyond the assessment of each single tissue-specific antibody, was able to predict the risk of irAEs irrespectively of the specific type of toxicity. Of note, the total levels of IgG/IgM tested with ELISA were not able to discriminate patients with a higher probability to develop irAEs, indicating the higher sensitivity in AutoAb detection of the array technology. The retrospective nature, the small sample size and the low proportion of patients with blood samples collected at the time of irAE represent additional limitations of our analysis. A prospective study is currently ongoing at our institution to validate these observations (NCT04107311). If validated in a prospective cohort, autoantibody profiling might represent a useful tool to identify upfront patients at high risk of clinically relevant irAEs through a simple blood draw.

### Supplementary Information

**Additional file 1: Supplementary Table 1.** Summary of Antigens included in the Microarray.

**Additional file 2: Supplementary Table 2. **Screening MFI Distribution by Age Group.

**Additional file 3: Supplementary Table 3. **Baseline (pre-ICI) distribution of single AutoAb by irAEs Status.

**Additional file 4: Supplementary Table 4. **Autoantibodies with significantly different mean MFI levels at baseline (pre-ICI) in patients with and without irAEs.

**Additional file 5: Supplementary Table 5. **AutoAbs Distribution by History of Autoimmune Disease Status

**Additional file 6: Supplementary Table 6.** AutoAb distrubution by specific types of irAEs.

**Additional file 7: Supplementary Table 7. **Selected cases of patients in which a significant elevation of autoAb correlated with organ-related toxicity.

**Additional file 8: Supplementary Fig. 1**. Frequencies of different irAEs G≥2.

**Additional file 9: Supplementary Fig. 2. **Changes in IgM and IgG levels in 61 patients without irAEs from baseline (pre-ICI collection) to the first collection after ICI administration.

**Additional file 10: Supplementary Fig. 3. **Dynamic changes of autoAbs in patients with and without irAEs G≥2.

**Additional file 11: Supplementary Fig. 4. **Comparison of the level of IgM and IgG with MFI> 500 at the time of irAEs and post steroids administration in 9 patients.

**Additional file 12: Supplementary Fig. 5.** Comparison of AutoAbs levels measured with ELISA in patients with and without irAEs G≥2 and dynamic changes in patients who developed irAEs G>2.

## Data Availability

Data supporting this study are included in the article and/or in the supporting materials.

## Abbreviations

ANA: Antinuclear antibodies
AutoAb: Autoantibody
AutoAg: Autoantigen
CTCAE: Common Terminology Criteria for Adverse Events
CTLA4: Cytotoxic T-lymphocyte-associated antigen 4
ELISA: Enzyme-linked immunosorbent assay
G: Grade
ICI: Immune checkpoint inhibitor
IgG: Immunoglobulin G
IgM: Immunoglobulin M
IQR: Interquartile range
irAEs: Immune-related adverse events
MFI: Median fluorescence intensity
OD: Optical density
PD-1: Programmed cell death 1
PD-L1: Programmed cell death 1 ligand
SCCHN: Squamous cell cancer of the head and neck
TAAs: Tumor associated antigens
