# Supplementary material for: Autoimmune PaneLs as PrEdictors of Toxicity in Patients TReated with Immune Checkpoint InhibiTors (ALERT)
Source: J Exp Clin Cancer Res. 2023 Oct 21;42:276. doi: 10.1186/s13046-023-02851-6 (PMC10589949; doi:10.1186/s13046-023-02851-6)
Supplement: Supplementary file 1 — Additional file 1: Supplementary Table 1. Summary of Antigens included in the Microarray. [file 13046_2023_2851_MOESM1_ESM.pdf]

Supplementary Table 1 Summary of Antigens included in the Microarray

Antigen Name (Used in Assay)	Full name	Explanation / Species	Company	Catalog ID
A1AR	Alpha 1A adrenergic receptor (A1AR) fragm	Peptide	GenScript	custom peptide
Actin (Bovine)	Actin	Bovine muscle	Sigma	A3653
Actin Rabbit	Actin (rabbit muscle)	Rabbit muscle	Molecular Probes	A12375
Aggrecan	Aggrecan	Bovine articular cartilage	Sigma	A1960
Aldolase, Type X	Aldolase, Type X	Rabbit muscle	Sigma	A2714
Alpha elastin	Alpha-elastin	Recombinant, human	Dr. Keeley (University of Toronto)	recombinant protein prepared in-house
alpha-Actinin	Alpha-actinin	Chicken gizzard	Sigma	A9776
Alpha-KGDH	Alpha-ketoglutarate dehydrogenase	Porcine heart	Sigma	K1502
alphaB-crystallin	Alpha B-crystallin	Recombinant, human	Enzo (stressgen)	ADI-SPP-228
Asparaginyl-tRNA Synthetase	Asparaginyl-tRNA Synthetase (KS)	Recombinant, human	Diarect	30100
AT1	angiotensin 2 receptor (AT1R) fragment	Peptide	GenScript	custom peptide
B1AR	Beta 1 adrenergic receptor (B1AR) fragme	Peptide	GenScript	custom peptide
B2AR	Beta 2 adrenergic receptor (B2AR) fragme	Peptide	GenScript	custom peptide
BCOADC-E2	branched chain 2-oxo-acid dehydrogenase	Recombinant, human	Diarect	17700
Beta 2 Glyc (recombinant human)	Beta 2 Glycoprotein I	Recombinant, human	Diarect	14900
beta 2 GPI (non-recombinant) (Bovine)	Beta 2 Glycoprotein I	Non-recombinant, bovine	Diarect	11400
beta 2 GPI (non-recombinant) (Human)	Beta 2 Glycoprotein I	Non-recombinant, human	Diarect	11300
Beta galactosidase 2B	Beta galactosidase 2B	Recombinant Protein	Creative Enzymes	NATE-1397
Bovin Histone H4 and H2A	Bovine Histone H4 and H2A	Purified from bovine thymus	Immunovision	HIS-1002
Bovin Histone subclass F1	Bovine Histone subclass F1	Purified from bovine tissue	Immunovision	HIS-1001
Bovine Histone H2b (F2b)	Bovine Histone H2b (F2b)	Purified from bovine tissue	Immunovision	HIS-1003
Bovine Histone H3	Bovine Histone H3	Purified from bovine tissue	Immunovision	HIS-1004
BPI	Bactericidal/Permeability Increasing protei	Non-recombinant, human	Diarect	19200
C1q (purified non-recombinant)	Complement component C1q	Purified from human serum	Sigma	C1740
Carbonic Anhydrase	Carbonic Anhydrase VI (CAG)	Recombinant	MyBiosource	MBS2011329
Cardiolipin C1649	Cardiolipin	Bovine heart, solution in ethanol	Sigma	C1649
CENP-A	Centromere Protein A	Recombinant, human	Diarect	16900
CENP-B	Centromere Protein B	Recombinant, human	Diarect	12500
Collagen I	Collagen, I	Human placenta	Sigma	C7774
Collagen III	Collagen, III	Human placenta	Sigma	C4407
Collagen IV	Collagen, IV	Human placenta	Sigma	C5533
Collagen V	Collagen, V	Human placenta	Sigma	C3657
Collagen VI	Collagen, VI	Human placenta	Sigma	C7521
Desmin	Desmin	Recombinant, human	GenWay	GWB-2E8E72
DNA Topoisomerase I (Scl-70; non recomb	DNA Topoisomerase I	Non-recombinant, bovine	Diarect	11500
dsDNA (genomic)	Deoxyribonucleic acid, double stranded	Salmon testes, sodium salt	Sigma	D1626
dsDNA (plasmid)	DNA circualar plasmid, Double Stranded	Bacterial plasmid	Diarect	12300
Ebna peptide	EBV nuclear antigen	398-412 peptide	Stanford	custom peptide
Enolase	Enolase	Rabbit muscle	Sigma	E0379
Fib I	Fibrinogen, type I	Human plasma	Sigma	F3879
Fib I-S	Fibrinogen, type I-S	Bovine plasma	Sigma	F8630
Fib IV	Fibrinogen, type IV	Bovine plasma	Sigma	F4753

GBM, diss	Glomerular Basement Membrane (GBM; d	Recombinant, human	Diarect	16800
Gliadin	Gliadin	Recombinant	Diarect	19500
Glycyl-tRNA Synthetase (EJ)	Glycyl-tRNA synthetase (EJ)	Recombinant, human	Diarect	11100
GP2	Zymogen granule membrane glycoprotein	Recombinant, human	Diarect	19600
gp210	glycoprotein 210	Recombinant, human	Diarect	19000
Grp78/BiP	GRP78 (HSPa5 or BiP) immunoglobulin hea	Recombinant hamster	Enzo (Stressgen)	ADI-SPP-765
HCEC cytop	Human Cardiac Endothelial Cells Lysate	Human	Dr. Viv Rao's lab	custom lysate prepared in-house
HCEC memb	Human Cardiac Endothelial Cell Lysate	Human	Dr. Viv Rao's lab	custom lysate prepared in-house
HCEC total (SDS)	Human Cardiac Endothelial Cell Lysate	Human	Dr. Viv Rao's lab	custom lysate prepared in-house
HCEC total (Triton)	Human Cardiac Endothelial Cell Lysate	Human	Dr. Viv Rao's lab	custom lysate prepared in-house
Heparin	Heparin Sulfate	Bovine kidney, sodium salt	Sigma	H7640
Histone H2A-H2B dimers	Histone H2A-H2B dimers	Recombinant, human	EMDMillipore	14-1052
Histone H2B	Histone H2B	Recombinant, human	Active Motif	31492
Histone H3 (1-136 aa)	Histone H3 (1-136 aa)	Recombinant, human	RayBiotech	268-11222-1
Histone H4 (1-103 aa)	Histone H4 (1-103 aa)	Recombinant, human	RayBiotech	268-11223-1
HMG CoA	3-hydroxy-3-methylglutaryl-Coenzyme A R	Recombinant, human	Cayman	14944
HSP 27	Heat shock protein 27	Recombinant, human	Enzo (Stressgen)	ADI-SPP-715
HSP 40	Heat shock protein 40	Recombinant, human	Enzo (Stressgen)	ADI-SPP-400
HSP 47	Heat shock protein 47	Recombinant, human	Enzo (Stressgen)	ADI-SPP-535
HSP 60	Heat shock protein 60	Recombinant, human	Enzo (Stressgen)	ADI-NSP-540-E
HSP 70	Heat shock protein 70	Recombinant, human	Enzo (Stressgen)	ADI-ESP-555
HSP 90	Heat shock protein 90	Native Human, from HeLa Cells	Enzo (Stressgen)	ADI-SPP-770
human albumin	Human albumin	recombinant, expressed in rice, lyophilized	Sigma	A9731
human C1q (Abcam)	Native Human C1q protein	Source: plasma	Abcam	ab96363
Human core histones	Human core histones	Purified from Hela cells	RayBiotech	268-11229-1
human fgl2	fibrinogen-like 2	Recombinant, human	Dr. Gary Levy (University Health Network)	recombinant protein prepared in-house
Human H1 Chromatin	Human H1 Chromatin	Purified protein	Dr. Joan Wither(University Health Network	prepared in-house
Human IgA	Human IgA	Purified from human serum	Jackson Immunoresearch	009-000-011
Human IgE	Human IgE	From a monoclonal hybridoma, human	Abcam	ab65866
Human IgG	Human IgG	ChromPure human IgG, whole molecule	Jackson Immunoresearch	009-000-003
human IgG F(ab)2	Human IgG F(ab)2 fragment	Purified from human serum	Jackson Immunoresearch	009-000-006
human IgG Fc	Human IgG Fc fragment	Purified from human serum	Jackson Immunoresearch	009-000-008
Human IgM	Human IgM	ChromPure human IgM, myeloma	Jackson Immunoresearch	009-000-012
human intestinal smooth muscle cell	Human Intestinal Smooth Muscle Cell Lysa	Lysate from primary human cells	ScienCell Research Laboratories	2916
Human LEDGF	lens epithelium-derived growth factor) or c	Recombinant, human	R&D Systems	3468-LE-050
Human LGALS3/Galectin 3	Human LGALS3/Galectin 3	Recombinant, human	LSBio	LS-G478
human nucleosome	Human nucleosome	Purified from Hela cells	EpiGex	EPX-01-NCN
ICAM-1	Inter-Cellular Adhesion Molecule 1	Recombinant, human	ProSci	96-400
Insulin	Insulin	Recombinant, human	Sigma	I2643
Intrinsic Factor	Intrinsic Factor	Recombinant, human	Diarect	16700
Jo-1	Histidyl-tRNA synthetase	Recombinant, human	Diarect	12900
kidney endothelial cell lysate	Kidney Endothelial Cell Lysate	Lysate from primary cell line	Dr. Anna Konvalinka (University Health Net	custom lysate
Ku (p70/p80)	Ku (p70/p80)	Recombinant, human	Diarect	17300
La (SS-B) Antigens (Immunovision)	La (SS-B) Antigens (47 kD ribonucleic prote	Purified from calf and/or rabbit thymus	Immunovision	SSB-3000
La/SS-B (Recombinant, human diarect)	La (SS-B) Antigens (47 kD ribonucleic prote	Recombinant, human	Diarect	12800

Laminin	Laminin	Engelbreth-Holm-Swarm murine sarcoma l	Sigma	L2020
LCI	liver cytosol type 1 antigen	Recombinant, human	Diarect	13700
LG3	immunogenic fragment of perlecan	Recombinant, human	Dr. Marie-Josee Hebert (University of Mon	recombinant protein prepared in-house
LKM 1 hp	Cytochrome p450 2D6	Recombinant, human	Diarect	19800
M2	The branched-chain α-ketoacid dehydroge	Recombinant, human	Diarect	18000
M2AR	M2 Muscarinc Receptor	Part of the second extracellular loop	GenScript	custom peptide
MDA5	melanoma differentiation-associated prote	Recombinant, human	Diarect	30000
Measles	Measles antigen	Edmonston Strain ATCC #VR-24	Meridian	R14120
Mi-2	Subunit of the nucleosome remodeling dea	Recombinant, human	Diarect	18100
mouse fgl2	Mouse fibrinogen-like 2	Recombinant, mouse	Dr. Gary Levy (University Health Network)	recombinant protein prepared in-house
mouse IgG F(ab')2	mouse IgG F(ab')2	Purified from serum	Jackson Immunoresearch	015-000-006
mouse IgG Fc	mouse IgG Fc	Purified from serum	Jackson Immunoresearch	015-000-008
mouse IgM	mouse IgM	Purified from serum	Biolegend	MM-30
MPO	Myeloperoxidase (MPO)	Nonrecombinant, human	Diarect	18500
Muscarinic Receptor 3	Muscarinic acetylcholine receptor 3, fragm	Peptide; second extracellular loop: KRTVPF	GenScript	custom peptide
MYH6	alpha cardiac myosin heavy chain	Recombinant, human	CUSABIO	CSB-YP015299HU
Myosin Bind Protein C	Myosin Binding Protein C	Recombinant, human	Dr. Sadayappan (Loyola University)	recombinant protein prepared in-house
Myosin	Myosin, calcium activated	Rabbit muscle	Sigma	M1636
Nucleolin	Nucleolin	Recombinant, human	Diarect	19700
Nucleosome (non-recombinant; bovine)	Nucleosome	Non-recombinant, bovine	Diarect	31000
Nup62	nucleoporin Nup62	Recombinant, human	Diarect	A194
OGDC-E2	2-oxoglutarate dehydrogenase complex	Recombinant, human	Diarect	17800
Ox LDL	Copper Oxidized human LDL	Human plasma	Academy Bio-Medical Company	20P-OX-L102
PBS	phosphate buffered saline		Wisent Bioproducts	311-010-CL
PCNA	Proliferating cell nuclear antigen	Recombinant, human	Diarect	36600
PDC-E2	E2 component of mitochondrial pyruvate d	Recombinant, human	Diarect	A179
PDH	Pyruvate dehydrogenase	Porcine heart	Sigma	P7032
PL-12	Alanyl-tRNA Synthase (PL-12)	Recombinant, human	Diarect	15700
PL-7	Threonyl-tRNA Synthase (PL-7)	Recombinant, human	Diarect	15600
PM/Scl 100	polymyositis/scleroderma (PM/Scl) comple	Recombinant, human	Diarect	16000
PM/Scl 75	polymyositis/scleroderma (PM/Scl) comple	Recombinant, human	Diarect	17000
Porcine Myosin Heart	Myosin, calcium activated, porcine heart	Porcine heart	Sigma	M0531
PR3	Proteinase 3	Non-recomb human leukocytes	Diarect	PR3
Proteoglycan	Proteoglycan	Bovine nasal septum	Sigma	P5864
Recombinant Histone H2A (hu)	Recombinant Histone H2A (hu)	Recombinant, human	Active Motif	31490
Ribo P0	Ribosomal Phosphoprotein P0	Recombinant, human	Diarect	14100
Ribo P1	Ribosomal Phosphoprotein P1	Recombinant, human	Diarect	14200
Ribo P2	Ribosomal Phosphoprotein P2	Recombinant, human	Diarect	14300
RNP/Sm (non-recombinant; bovine)	RNP/Sm (non-recombinant; bovine)	Non-recombinant, bovine	Diarect	11600
Ro/SS-A (52 kDa, human recombinant)	Ro/SS-A (52 kDa)	Recombinant, human	Diarect	12700
Ro/SS-A (60 kD; non-recombinant; bovine)	Ro/SS-A (60 kDa)	Non-recombinant bovine	Diarect	15500
Ro/SS-A (60kD; recombinant)	Ro/SS-A (60 kDa)	Recombinant, human	Diarect	17400
SCGB1A1	Secretoglobin family 1A member 1	Recombinant, human	Sigma	APrEST78092
Scl-70 Full	DNA Topoisomerase I (Scl-70) full length	Recombinant, human	Diarect	12400
Scl-70: trunc	DNA Topoisomerase I (Scl-70) truncated	Recombinant, human	Diarect	14500

Sm (non-recombinant; bovine)	Sm	Non-recombinant, bovine	Diarect	17500
Sm Antigens	Sm Antigens	Purified from bovine thymus	Immunovision	SMA-3000
SmD	SmD	Recombinant, human	Diarect	11700
SmD1	Human small nuclear ribonucleoprotein D1	Recombinant, human	Diarect	11800
SmD2	Human small nuclear ribonucleoprotein D2	Recombinant, human	Diarect	11900
SmD3	Human small nuclear ribonucleoprotein D3	Recombinant, human	Diarect	12000
snRNP 68	U1-snRNP 68 Protein	Recombinant, human	Diarect	13000
snRNP 68 B/B	U1-snRNP 68 Protein B/B	Recombinant, human	Diarect	13300
snRNP A	U1-snRNP A Protein	Recombinant, human	Diarect	13100
snRNP C	U1-snRNP C Protein	Recombinant, human	Diarect	13200
SP-D	Surfactant protein D	Recombinant, human	Novusbio	1920-SP
Sp100	Sp100 nuclear antigen (Speckled 100 kDa)	Recombinant, human	Diarect	18900
SPLUNC2	Human Parotid Secretory Protein (SPLUNC	Recombinant, human	MyBiosource	MBS2122495
SRP54	signal recognition particle (SRP) 54 GTPase	Recombinant, human	Diarect	18400
ssDNA	Deoxyribonucleic acid, single stranded	Calf thymus	Sigma	D8899
thyroglobulin #2	dimeric glycoprotein thyroglobulin	Native. Isolated from human thyroid gland	Diarect	A12200
Thyrogobulin	dimeric glycoprotein thyroglobulin	Native. Isolated from human thyroid gland	Diarect	A12200
TIF1 gamma	transcription intermediary factor 1-gamma	Recombinant, human	Diarect	11000
TPO	Thyroid peroxidase (TPO)	Recombinant, human	Diarect	12100
Tropoelastin	Tropoelastin	Recombinant, human	Dr. Keeley (University of Toronto)	recombinant protein prepared in-house
Tropomyosin	Tropomyosin	Porcine muscle	Sigma	T2400
Troponin C	Troponin C	Recombinant, human	CalBioreagents	A093
Troponin I	Troponin I	Human heart	Sigma	T9924
Troponin T	Troponin T	Human heart	Sigma	T0175
tTG baculovirus	tTG (Tissue transglutaminase) baculovirus	Recombinant, human	Diarect	15200
tTG E. coli	tTG (Tissue transglutaminase) E. coli	Recombinant, human	Diarect	14400
TUBA1B protein	Tubulin alpha-1B chain	Recombinant, human	Abnova	H00010376-P01
Vimentin	Vimentin	Recombinant, human	Cedarlane	CLPR0309
whole histones	Whole Histones	Purified from chicken RBC	Immunovision	HIS-1000
whole mouse IgG	mouse IgG	Purified from serum	Jackson immunoresearch	015-000-003

. .
