# Supplementary material for: Autoimmune PaneLs as PrEdictors of Toxicity in Patients TReated with Immune Checkpoint InhibiTors (ALERT)
Source: J Exp Clin Cancer Res. 2023 Oct 21;42:276. doi: 10.1186/s13046-023-02851-6 (PMC10589949; doi:10.1186/s13046-023-02851-6)
Supplement: Supplementary file 2 — Additional file 2: Supplementary Table 2. Screening MFI Distribution by Age Group. [file 13046_2023_2851_MOESM2_ESM.docx]

Supplementary table 2: Screening MFI Distribution by Age Group. MFI distributions are summarized by age group (≤45 vs. >45, ≤50 vs. >50, ≤60 vs. >60). Differences in distribution are assessed using the Mann-Whitney U test.

# <=45 vs. >45

|  | <=45 (n=20) | >45 (n=94) | p-value |
| --- | --- | --- | --- |
| **Screening IgM 500** |  |  | 0.12 |
| Mean (sd) | 37.4 (24.7) | 29.0 (19.6) |  |
| Median (Q1,Q3) | 28.0 (24.5, 41.5) | 24.0 (14.0, 40.8) |  |
| Range (min, max) | (9, 99) | (4, 88) |  |
| **Screening IgG 500** |  |  | 0.23 |
| Mean (sd) | 42.0 (15.2) | 37.5 (13.3) |  |
| Median (Q1,Q3) | 42.0 (29.8, 56.5) | 34.0 (28.2, 43.8) |  |
| Range (min, max) | (21, 69) | (15, 80) |  |
| **Screening Both 500** |  |  | 0.06 |
| Mean (sd) | 79.3 (29.1) | 66.5 (26.2) |  |
| Median (Q1,Q3) | 73.5 (58.8, 96.8) | 60.0 (49.2, 85.8) |  |
| Range (min, max) | (33, 143) | (25, 139) |  |
| **Screening IgM 1500** |  |  | 0.24 |
| Mean (sd) | 10.7 (8.5) | 8.7 (7.4) |  |
| Median (Q1,Q3) | 7.5 (5.8, 12.2) | 7.0 (3.2, 10.0) |  |
| Range (min, max) | (3, 36) | (2, 37) |  |
| **Screening IgG 200** |  |  | 0.14 |
| Mean (sd) | 72.2 (18.5) | 65.9 (15.4) |  |
| Median (Q1,Q3) | 69.5 (59.2, 88.5) | 64 (55, 75) |  |
| Range (min, max) | (38, 111) | (37, 116) |  |
| **Screening Both 950** |  |  | 0.13 |
| Mean (sd) | 44.6 (17.2) | 38.0 (15.2) |  |
| Median (Q1,Q3) | 43.5 (30.0, 55.5) | 33 (27, 48) |  |
| Range (min, max) | (19, 80) | (15, 81) |  |

# <=50 vs. >50

|  | <=50 (n=29) | >50 (n=85) | p-value |
| --- | --- | --- | --- |
| **Screening IgM 500** |  |  | 0.12 |
| Mean (sd) | 35.3 (22.9) | 28.8 (19.8) |  |
| Median (Q1,Q3) | 29 (21, 41) | 24 (14, 41) |  |
| Range (min, max) | (7, 99) | (4, 88) |  |
| **Screening IgG 500** |  |  | 0.15 |
| Mean (sd) | 42.3 (15.7) | 36.9 (12.7) |  |
| Median (Q1,Q3) | 41 (29, 56) | 34 (29, 42) |  |
| Range (min, max) | (21, 73) | (15, 80) |  |
| **Screening Both 500** |  |  | **0.04** |
| Mean (sd) | 77.6 (28.4) | 65.7 (26.0) |  |
| Median (Q1,Q3) | 73 (58, 94) | 60 (49, 85) |  |
| Range (min, max) | (29, 143) | (25, 139) |  |
| **Screening IgM 1500** |  |  | 0.30 |
| Mean (sd) | 9.6 (7.4) | 8.9 (7.7) |  |
| Median (Q1,Q3) | 7 (6, 11) | 7 (3, 10) |  |
| Range (min, max) | (3, 36) | (2, 37) |  |
| **Screening IgG 200** |  |  | 0.09 |
| Mean (sd) | 72.0 (18.1) | 65.4 (15.1) |  |
| Median (Q1,Q3) | 70 (57, 88) | 64 (55, 75) |  |
| Range (min, max) | (38, 111) | (37, 116) |  |
| **Screening Both 950** |  |  | 0.10 |
| Mean (sd) | 43.1 (16.0) | 37.8 (15.5) |  |
| Median (Q1,Q3) | 42 (31, 51) | 33 (27, 48) |  |
| Range (min, max) | (19, 80) | (15, 81) |  |

# <=60 vs. >60

|  | <=60 (n=56) | >60 (n=58) | p-value |
| --- | --- | --- | --- |
| **Screening IgM 500** |  |  | 0.14 |
| Mean (sd) | 33.5 (22.0) | 27.5 (19.2) |  |
| Median (Q1,Q3) | 27.5 (16.8, 43.5) | 21.5 (14.0, 31.0) |  |
| Range (min, max) | (4, 99) | (6, 88) |  |
| **Screening IgG 500** |  |  | 0.47 |
| Mean (sd) | 40.1 (15.9) | 36.6 (11.1) |  |
| Median (Q1,Q3) | 34.5 (28.0, 52.0) | 34.5 (29.0, 42.0) |  |
| Range (min, max) | (15, 80) | (18, 63) |  |
| **Screening Both 500** |  |  | 0.07 |
| Mean (sd) | 73.6 (29.7) | 64.1 (23.4) |  |
| Median (Q1,Q3) | 71.5 (53.0, 95.2) | 59.0 (49.2, 80.0) |  |
| Range (min, max) | (25, 143) | (29, 139) |  |
| **Screening IgM 1500** |  |  | 0.22 |
| Mean (sd) | 9.6 (7.2) | 8.6 (8.0) |  |
| Median (Q1,Q3) | 7.0 (4.8, 12.0) | 6.5 (3.2, 9.0) |  |
| Range (min, max) | (2, 36) | (2, 37) |  |
| **Screening IgG 200** |  |  | 0.34 |
| Mean (sd) | 69.0 (18.1) | 65.2 (13.7) |  |
| Median (Q1,Q3) | 65.0 (57.0, 85.2) | 64.0 (54.2, 75.0) |  |
| Range (min, max) | (37, 116) | (38, 95) |  |
| **Screening Both 950** |  |  | 0.24 |
| Mean (sd) | 41.1 (16.8) | 37.2 (14.4) |  |
| Median (Q1,Q3) | 40.5 (27.8, 51.0) | 32.5 (27.0, 45.8) |  |
| Range (min, max) | (15, 80) | (17, 81) |  |
