# Supplementary material for: Autoimmune PaneLs as PrEdictors of Toxicity in Patients TReated with Immune Checkpoint InhibiTors (ALERT)
Source: J Exp Clin Cancer Res. 2023 Oct 21;42:276. doi: 10.1186/s13046-023-02851-6 (PMC10589949; doi:10.1186/s13046-023-02851-6)
Supplement: Supplementary file 3 — Additional file 3: Supplementary Table 3. Baseline (pre-ICI) distribution of single AutoAb by irAEs Status. [file 13046_2023_2851_MOESM3_ESM.docx]

**Supplementary Table 3:** **baseline (pre-ICI) distribution of single AutoAb by irAEs Status.**

|  | **Full Sample (n=114)** | **No**  **(n=77)** | **Yes**  **(n=37)** | **Unadj. P-Value** | **Adj.**  **P-Value** |  |
| --- | --- | --- | --- | --- | --- | --- |
| **IgG SP D** |  |  |  | 0.62 | 0.851 |  |
| Mean (sd) | 6563.4 (4158.6) | 6344.4 (3771.9) | 7019.2 (4892.1) |  |  |  |
| Median (Q1,Q3) | 5368.0 (3743.6, 8751.8) | 5231.0 (3724.5, 8779.5) | 5916.0 (4022.0, 8439.5) |  |  |  |
| Range (min, max) | (481.5, 25235.5) | (481.5, 14759.0) | (1577.5, 25235.5) |  |  |  |
| **IgG Bovin Histone H4 and H2A** |  |  |  | **0.04** | 0.325 |  |
| Mean (sd) | 318.4 (476.9) | 246.6 (292.2) | 467.8 (706.9) |  |  |  |
| Median (Q1,Q3) | 173.5 (111.0, 331.6) | 157.0 (110.5, 285.5) | 205.5 (144.5, 400.5) |  |  |  |
| Range (min, max) | (5, 3701) | (5, 2194) | (65.5, 3701.0) |  |  |  |
| **IgG Human core histones** |  |  |  | 0.36 | 0.715 |  |
| Mean (sd) | 385.2 (431.1) | 332.9 (344.3) | 494.1 (561.4) |  |  |  |
| Median (Q1,Q3) | 243.0 (173.8, 410.8) | 242.0 (170.5, 392.5) | 244.0 (187.0, 479.5) |  |  |  |
| Range (min, max) | (32, 2651) | (32, 2651) | (93, 2482) |  |  |  |
| **IgG Human IgG** |  |  |  | 0.87 | 0.947 |  |
| Mean (sd) | 36708.1 (6729.3) | 36825.6 (7165.8) | 36463.7 (5800.5) |  |  |  |
| Median (Q1,Q3) | 35671.2 (31400.4, 42783.5) | 34786.0 (31329.5, 43141.5) | 35828.0 (32395.5, 41919.0) |  |  |  |
| Range (min, max) | (19863.5, 52294.5) | (19863.5, 52294.5) | (23401.0, 46227.5) |  |  |  |
| **IgG HCEC cytop** |  |  |  | 0.93 | 0.965 |  |
| Mean (sd) | 594.6 (300.2) | 597.8 (312.9) | 587.7 (275.8) |  |  |  |
| Median (Q1,Q3) | 501.2 (386.6, 676.9) | 500.5 (390.0, 686.0) | 530.5 (375.5, 670.5) |  |  |  |
| Range (min, max) | (243.5, 1576.5) | (251.5, 1576.5) | (243.5, 1295.0) |  |  |  |
| **IgG MDA5** |  |  |  | 0.47 | 0.794 |  |
| Mean (sd) | 127.0 (1055.6) | 198.2 (1259.3) | -21.1 (340.4) |  |  |  |
| Median (Q1,Q3) | -115.5 (-213.5, 11.6) | -123.5 (-234.0, 2.0) | -109.0 (-201.5, 53.5) |  |  |  |
| Range (min, max) | (-371.0, 7326.5) | (-371.0, 7326.5) | (-324.5, 1625.0) |  |  |  |
| **IgG Gliadin** |  |  |  | 0.59 | 0.84 |  |
| Mean (sd) | 3345.2 (5069.7) | 3212.1 (4548.2) | 3622.2 (6073.6) |  |  |  |
| Median (Q1,Q3) | 1893.2 (684.2, 3982.6) | 2042.5 (638.5, 3987.5) | 1890.0 (787.5, 3959.0) |  |  |  |
| Range (min, max) | (-91.0, 36447.5) | (-91.0, 23984.5) | (86.0, 36447.5) |  |  |  |
| **IgG tTG E coli** |  |  |  | 0.13 | 0.449 |  |
| Mean (sd) | 86.7 (215.1) | 84.0 (243.5) | 92.3 (141.5) |  |  |  |
| Median (Q1,Q3) | 20.2 (-13.2, 117.8) | 12.5 (-17.0, 114.0) | 77.0 (2.5, 133.5) |  |  |  |
| Range (min, max) | (-107.5, 1745.0) | (-107.5, 1745.0) | (-79.0, 579.5) |  |  |  |
| **IgG Myosin Bind Protein C** |  |  |  | 0.62 | 0.851 |  |
| Mean (sd) | 1036.7 (1975.8) | 1152.6 (2348.7) | 795.6 (720.6) |  |  |  |
| Median (Q1,Q3) | 537.5 (298.5, 1022.2) | 542.0 (313.5, 982.5) | 447 (246, 1164) |  |  |  |
| Range (min, max) | (17.5, 19076.0) | (17.5, 19076.0) | (69, 2586) |  |  |  |
| **IgG Tropoelastin** |  |  |  | 0.59 | 0.842 |  |
| Mean (sd) | 591.6 (599.3) | 585.0 (529.9) | 605.4 (731.0) |  |  |  |
| Median (Q1,Q3) | 385.0 (167.6, 833.6) | 398.5 (194.0, 841.5) | 370.0 (153.5, 810.0) |  |  |  |
| Range (min, max) | (-11.0, 3190.5) | (-4.5, 2226.0) | (-11.0, 3190.5) |  |  |  |
| **IgG Insulin** |  |  |  | **0.05** | 0.327 |  |
| Mean (sd) | -59.8 (40.5) | -65.6 (20.1) | -47.7 (63.9) |  |  |  |
| Median (Q1,Q3) | -62.5 (-78.5, -51.5) | -65.0 (-80.5, -53.5) | -58.0 (-72.5, -49.0) |  |  |  |
| Range (min, max) | (-116.0, 302.5) | (-116, 22) | (-93.5, 302.5) |  |  |  |
| **IgG Tropomyosin** |  |  |  | 0.65 | 0.852 |  |
| Mean (sd) | 1127.8 (1418.7) | 1147.0 (1471.9) | 1087.9 (1319.5) |  |  |  |
| Median (Q1,Q3) | 644.2 (289.9, 1285.6) | 635.5 (279.0, 1214.0) | 689.5 (315.0, 1287.0) |  |  |  |
| Range (min, max) | (32.5, 8821.0) | (32.5, 8821.0) | (149.0, 7472.5) |  |  |  |
| **IgG Myosin M1636** |  |  |  | 0.84 | 0.94 |  |
| Mean (sd) | -21.9 (62.3) | -18.0 (73.3) | -29.8 (26.7) |  |  |  |
| Median (Q1,Q3) | -33.8 (-53.0, -6.5) | -35 (-58, -2) | -33.5 (-39.5, -22.0) |  |  |  |
| Range (min, max) | (-103.5, 434.5) | (-103.5, 434.5) | (-82.5, 45.5) |  |  |  |
| **IgG Jo 1** |  |  |  | 0.07 | 0.366 |  |
| Mean (sd) | 218.5 (820.3) | 151.8 (595.5) | 357.1 (1155.0) |  |  |  |
| Median (Q1,Q3) | 35.0 (-20.0, 141.2) | 15 (-28, 116) | 60.0 (-4.5, 207.0) |  |  |  |
| Range (min, max) | (-120.0, 6874.5) | (-120, 4915) | (-112.5, 6874.5) |  |  |  |
| **IgG Ribo P1** |  |  |  | 0.34 | 0.689 |  |
| Mean (sd) | 205.1 (292.1) | 219.7 (340.9) | 174.8 (145.0) |  |  |  |
| Median (Q1,Q3) | 152.0 (94.1, 250.2) | 154.0 (101.5, 266.0) | 148.5 (70.0, 199.0) |  |  |  |
| Range (min, max) | (-37.0, 2997.5) | (-37.0, 2997.5) | (-15, 590) |  |  |  |
| **IgG MPO** |  |  |  | **0.03** | 0.283 |  |
| Mean (sd) | -26.9 (115.1) | -33.6 (120.2) | -12.9 (103.7) |  |  |  |
| Median (Q1,Q3) | -48.0 (-85.2, -1.5) | -58.5 (-96.5, -13.0) | -28.5 (-68.0, 21.0) |  |  |  |
| Range (min, max) | (-239.5, 489.0) | (-239.5, 479.5) | (-145.5, 489.0) |  |  |  |
| **IgG Scl 70 Full** |  |  |  | 0.42 | 0.758 |  |
| Mean (sd) | 261.7 (433.9) | 239.7 (434.8) | 307.7 (434.5) |  |  |  |
| Median (Q1,Q3) | 134.5 (32.9, 330.0) | 126.5 (37.0, 269.0) | 160.0 (31.5, 387.0) |  |  |  |
| Range (min, max) | (-73, 2832) | (-73, 2832) | (-13, 1871) |  |  |  |
| **IgG Scl 70 trunc** |  |  |  | 0.63 | 0.852 |  |
| Mean (sd) | 312.5 (533.1) | 273.5 (483.5) | 393.5 (623.3) |  |  |  |
| Median (Q1,Q3) | 167.8 (63.4, 297.1) | 159.5 (63.0, 271.5) | 191.0 (72.0, 304.5) |  |  |  |
| Range (min, max) | (-90.0, 3178.5) | (-90.0, 3178.5) | (-54.5, 2506.5) |  |  |  |
| **IgG SPLUNC2** |  |  |  | **0.03** | 0.277 |  |
| Mean (sd) | 173.4 (107.6) | 187.2 (118.2) | 144.8 (74.6) |  |  |  |
| Median (Q1,Q3) | 148.2 (106.9, 210.2) | 164.0 (112.5, 228.5) | 121.0 (95.0, 181.5) |  |  |  |
| Range (min, max) | (40.0, 707.5) | (40.5, 707.5) | (40.0, 363.5) |  |  |  |
| **IgG Sm Antigens** |  |  |  | 0.64 | 0.852 |  |
| Mean (sd) | 438.7 (335.5) | 444.8 (363.8) | 426.0 (271.1) |  |  |  |
| Median (Q1,Q3) | 351.8 (241.6, 555.5) | 371.0 (248.0, 546.5) | 323 (225, 565) |  |  |  |
| Range (min, max) | (89.5, 2959.5) | (89.5, 2959.5) | (118.0, 1124.5) |  |  |  |
| IgG Histone H3 1 136 aa |  |  |  | 0.62 | 0.851 |  |
| Mean (sd) | 1205.9 (1015.9) | 1119.5 (865.9) | 1385.7 (1267.9) |  |  |  |
| Median (Q1,Q3) | 854.5 (468.9, 1647.2) | 819 (519, 1387) | 899.0 (437.5, 1825.5) |  |  |  |
| Range (min, max) | (56.5, 4457.0) | (56.5, 3559.5) | (279, 4457) |  |  |  |
| **IgG mouse IgM** |  |  |  | 0.76 | 0.896 |  |
| Mean (sd) | 319.5 (476.2) | 321.6 (507.8) | 315.0 (409.3) |  |  |  |
| Median (Q1,Q3) | 150.8 (67.9, 336.2) | 152.5 (65.0, 290.0) | 132.5 (83.5, 434.5) |  |  |  |
| Range (min, max) | (-35.0, 3354.5) | (-35.0, 3354.5) | (-12.0, 1938.5) |  |  |  |
| **IgG Beta galactosidase 2B** |  |  |  | 0.75 | 0.894 |  |
| Mean (sd) | 588.1 (1307.0) | 497.1 (849.1) | 777.5 (1945.6) |  |  |  |
| Median (Q1,Q3) | 218.0 (103.0, 493.9) | 202.5 (92.0, 501.0) | 230 (107, 379) |  |  |  |
| Range (min, max) | (-23.0, 11462.5) | (-23.0, 4772.5) | (15.5, 11462.5) |  |  |  |
| **IgG DNA Topoisomerase I Scl 70 non recombinant bovine** |  |  |  | **0.04** | 0.321 |  |
| Mean (sd) | 123.7 (235.3) | 79.7 (104.2) | 215.1 (371.7) |  |  |  |
| Median (Q1,Q3) | 55.0 (18.1, 136.1) | 45.5 (17.5, 107.0) | 98.5 (22.5, 181.0) |  |  |  |
| Range (min, max) | (-61, 1820) | (-61, 448) | (-18, 1820) |  |  |  |
| **IgG Sm non recombinant bovine** |  |  |  | **0.01** | 0.235 |  |
| Mean (sd) | 225.7 (476.3) | 218.0 (549.1) | 241.6 (273.8) |  |  |  |
| Median (Q1,Q3) | 86.0 (7.0, 236.9) | 46.5 (-1.0, 202.0) | 186.5 (35.5, 297.0) |  |  |  |
| Range (min, max) | (-77.5, 3915.0) | (-77.5, 3915.0) | (-42, 1058) |  |  |  |
| **IgG OGDC E2** |  |  |  | 0.06 | 0.36 |  |
| Mean (sd) | 124.9 (595.3) | 130.4 (703.9) | 113.2 (257.2) |  |  |  |
| Median (Q1,Q3) | -10.0 (-60.8, 68.6) | -17.5 (-67.5, 50.0) | 9.5 (-40.5, 189.5) |  |  |  |
| Range (min, max) | (-134, 4612) | (-124.5, 4612.0) | (-134, 973) |  |  |  |
| **IgG mouse fgl2** |  |  |  | 0.91 | 0.961 |  |
| Mean (sd) | 152.4 (193.5) | 163.1 (230.3) | 130.2 (69.3) |  |  |  |
| Median (Q1,Q3) | 111.0 (86.1, 154.8) | 110.5 (84.5, 165.5) | 111.5 (95.0, 138.5) |  |  |  |
| Range (min, max) | (27.5, 1848.0) | (27.5, 1848.0) | (32, 337) |  |  |  |
| **IgG HSP 70** |  |  |  | 0.58 | 0.839 |  |
| Mean (sd) | 636.2 (2931.2) | 783.1 (3558.3) | 330.5 (311.9) |  |  |  |
| Median (Q1,Q3) | 170.5 (91.1, 427.6) | 165.5 (87.0, 365.0) | 182.0 (109.0, 567.5) |  |  |  |
| Range (min, max) | (14.5, 31135.5) | (14.5, 31135.5) | (22.0, 1254.5) |  |  |  |
| **IgG HSP 60** |  |  |  | 0.67 | 0.854 |  |
| Mean (sd) | 1973.8 (5851.4) | 2191.8 (6995.1) | 1520.2 (1964.9) |  |  |  |
| Median (Q1,Q3) | 709.0 (217.6, 1812.2) | 707.0 (281.5, 1503.5) | 730.5 (161.5, 2153.0) |  |  |  |
| Range (min, max) | (3.5, 59911.0) | (3.5, 59911.0) | (35.0, 8460.5) |  |  |  |
| **IgG Collagen III C4407** |  |  |  | **0.008** | 0.207 |  |
| Mean (sd) | 59.5 (61.4) | 46.8 (43.4) | 85.8 (82.5) |  |  |  |
| Median (Q1,Q3) | 47.8 (20.8, 82.5) | 41.5 (17.0, 65.5) | 68.5 (40.0, 96.0) |  |  |  |
| Range (min, max) | (-25.5, 396.5) | (-25.5, 215.0) | (-12.0, 396.5) |  |  |  |
| **IgG Heparin** |  |  |  | 0.08 | 0.394 |  |
| Mean (sd) | 50.0 (85.1) | 56.9 (89.8) | 35.8 (73.3) |  |  |  |
| Median (Q1,Q3) | 25.2 (0.1, 59.4) | 27.5 (8.0, 59.5) | 9 (-12, 59) |  |  |  |
| Range (min, max) | (-46.5, 427.0) | (-44.5, 427.0) | (-46.5, 234.0) |  |  |  |
| **IgG Ebna peptide** |  |  |  | **0.02** | 0.272 |  |
| Mean (sd) | 686.0 (1641.3) | 848.9 (1911.4) | 346.8 (753.3) |  |  |  |
| Median (Q1,Q3) | 123.8 (-22.9, 491.8) | 167.5 (-13.0, 516.0) | 51 (-41, 363) |  |  |  |
| Range (min, max) | (-75.5, 11997.5) | (-57.0, 11997.5) | (-75.5, 3292.5) |  |  |  |
| **IgG Fib I S** |  |  |  | 0.06 | 0.365 |  |
| Mean (sd) | 374.5 (739.0) | 295.3 (589.8) | 539.3 (968.7) |  |  |  |
| Median (Q1,Q3) | 166.0 (70.1, 389.6) | 144.0 (65.5, 336.0) | 265.5 (97.5, 517.5) |  |  |  |
| Range (min, max) | (-19.0, 5456.5) | (-19, 4836) | (-14.0, 5456.5) |  |  |  |
| **IgG PL 12** |  |  |  | 0.92 | 0.963 |  |
| Mean (sd) | 1871.5 (1478.9) | 1947.1 (1588.6) | 1714.3 (1224.7) |  |  |  |
| Median (Q1,Q3) | 1302.2 (763.8, 2648.8) | 1267.5 (754.5, 2893.0) | 1432 (781, 2582) |  |  |  |
| Range (min, max) | (63, 6128) | (245.5, 6128.0) | (63.0, 5813.5) |  |  |  |
| **IgG La SS B Recombinant human diarect** |  |  |  | 0.64 | 0.852 |  |
| Mean (sd) | 745.6 (1330.8) | 816.6 (1499.1) | 597.8 (884.2) |  |  |  |
| Median (Q1,Q3) | 169.2 (71.0, 766.1) | 155 (65, 778) | 184.5 (117.0, 706.0) |  |  |  |
| Range (min, max) | (-48.5, 7487.5) | (-40.5, 7487.5) | (-48.5, 4607.0) |  |  |  |
| **IgG Ku p70 p80** |  |  |  | 0.32 | 0.684 |  |
| Mean (sd) | 184.8 (675.7) | 154.7 (412.5) | 247.5 (1033.4) |  |  |  |
| Median (Q1,Q3) | 39.0 (7.1, 107.0) | 34.0 (2.0, 107.5) | 45.0 (20.0, 105.5) |  |  |  |
| Range (min, max) | (-80, 6329) | (-56.5, 2570.5) | (-80, 6329) |  |  |  |
| **IgG empty** |  |  |  | 0.19 | 0.532 |  |
| Mean (sd) | -0.5 (4.4) | -1.1 (4.0) | 0.8 (5.1) |  |  |  |
| Median (Q1,Q3) | -1.0 (-2.5, 1.5) | -1.0 (-3.0, 1.5) | 0.0 (-2.5, 4.0) |  |  |  |
| Range (min, max) | (-12, 17) | (-12, 7) | (-7, 17) |  |  |  |
| **IgG Human H1 Chromatin** |  |  |  | 0.12 | 0.449 |  |
| Mean (sd) | 447.4 (488.1) | 431.2 (515.1) | 481.3 (431.4) |  |  |  |
| Median (Q1,Q3) | 257.8 (129.6, 589.8) | 221.0 (119.5, 532.5) | 374.0 (190.5, 599.5) |  |  |  |
| Range (min, max) | (-3.0, 2872.5) | (21.5, 2872.5) | (-3, 2188) |  |  |  |
| **IgG Human LGALS3 Galectin 3** |  |  |  | 0.12 | 0.44 |  |
| Mean (sd) | 3.3 (8.1) | 4.2 (9.2) | 1.5 (4.3) |  |  |  |
| Median (Q1,Q3) | 2.0 (-0.5, 5.5) | 3.0 (-0.5, 6.0) | 1.0 (-0.5, 3.5) |  |  |  |
| Range (min, max) | (-8.5, 63.5) | (-7.0, 63.5) | (-8.5, 11.0) |  |  |  |
| **IgG Human IgA** |  |  |  | 0.50 | 0.813 |  |
| Mean (sd) | 1286.4 (1970.5) | 1288.8 (2156.6) | 1281.4 (1539.1) |  |  |  |
| Median (Q1,Q3) | 626.8 (130.5, 1587.0) | 429.0 (101.5, 1607.0) | 766.5 (153.5, 1522.0) |  |  |  |
| Range (min, max) | (-5, 14356) | (-5, 14356) | (36.5, 5789.5) |  |  |  |
| **IgG kidney endothelial cell lysate** |  |  |  | 0.31 | 0.677 |  |
| Mean (sd) | 2062.5 (3459.7) | 1987.7 (3655.5) | 2218.1 (3053.5) |  |  |  |
| Median (Q1,Q3) | 1017.2 (414.9, 1774.0) | 1007.5 (400.0, 1730.5) | 1033.0 (593.5, 2273.0) |  |  |  |
| Range (min, max) | (135.0, 22763.5) | (158.5, 22763.5) | (135, 13998) |  |  |  |
| **IgG MYH6** |  |  |  | 0.09 | 0.433 |  |
| Mean (sd) | 4556.2 (4616.6) | 4971.8 (4843.6) | 3691.4 (4029.2) |  |  |  |
| Median (Q1,Q3) | 2763.8 (1137.4, 6670.2) | 3394.5 (1370.5, 7487.5) | 1915.5 (811.5, 6414.5) |  |  |  |
| Range (min, max) | (269.5, 25903.5) | (269.5, 25903.5) | (318.5, 18681.0) |  |  |  |
| **IgG Ro SS A 60kD recombinant** |  |  |  | **0.02** | 0.272 |  |
| Mean (sd) | 349.8 (1928.9) | 436.2 (2332.4) | 169.8 (381.5) |  |  |  |
| Median (Q1,Q3) | 22.2 (-17.9, 85.4) | 10.5 (-26.5, 75.0) | 55.5 (10.0, 107.0) |  |  |  |
| Range (min, max) | (-84.5, 16428.5) | (-84.5, 16428.5) | (-67.0, 1938.5) |  |  |  |
| **IgG Sp100** |  |  |  | 0.52 | 0.82 |  |
| Mean (sd) | 768.1 (2127.8) | 640.2 (1301.0) | 1034.2 (3245.2) |  |  |  |
| Median (Q1,Q3) | 158.2 (24.6, 485.4) | 156.5 (22.0, 490.0) | 186.0 (54.5, 471.5) |  |  |  |
| Range (min, max) | (-117.5, 19268.5) | (-117.5, 7673.5) | (-23.5, 19268.5) |  |  |  |
| **IgG B2AR** |  |  |  | 0.79 | 0.914 |  |
| Mean (sd) | -1.8 (22.4) | -2.0 (23.4) | -1.4 (20.5) |  |  |  |
| Median (Q1,Q3) | -0.5 (-16.9, 12.4) | -1.0 (-17.5, 11.0) | 0.5 (-13.0, 15.5) |  |  |  |
| Range (min, max) | (-48, 59) | (-48, 59) | (-46.5, 36.0) |  |  |  |
| **IgG AT1** |  |  |  | 0.85 | 0.944 |  |
| Mean (sd) | 40.8 (22.5) | 40.6 (21.8) | 41.1 (24.1) |  |  |  |
| Median (Q1,Q3) | 34.2 (24.6, 51.9) | 34.5 (26.0, 50.5) | 34.0 (22.5, 53.5) |  |  |  |
| Range (min, max) | (6.5, 102.0) | (6.5, 102.0) | (9.0, 101.5) |  |  |  |
| **IgG ICAM 1** |  |  |  | 0.11 | 0.44 |  |
| Mean (sd) | -17.8 (38.3) | -19.2 (42.6) | -14.7 (27.6) |  |  |  |
| Median (Q1,Q3) | -23.8 (-39.9, -2.2) | -26.5 (-41.0, -9.5) | -12 (-34, 7) |  |  |  |
| Range (min, max) | (-74, 260) | (-74, 260) | (-62.5, 36.0) |  |  |  |
| **IgG HSP 27** |  |  |  | 0.62 | 0.852 |  |
| Mean (sd) | 292.0 (560.5) | 223.4 (240.2) | 434.8 (912.9) |  |  |  |
| Median (Q1,Q3) | 153.2 (50.5, 340.9) | 150.5 (49.5, 327.5) | 154.5 (62.5, 356.0) |  |  |  |
| Range (min, max) | (-15.5, 4702.5) | (-15.5, 1468.5) | (-13.0, 4702.5) |  |  |  |
| **IgG Actin Bovine** |  |  |  | **0.02** | 0.252 |  |
| Mean (sd) | -1.3 (53.9) | -9.5 (30.1) | 15.8 (82.3) |  |  |  |
| Median (Q1,Q3) | -4.0 (-25.9, 15.2) | -11.5 (-32.0, 10.0) | 0.5 (-7.0, 18.0) |  |  |  |
| Range (min, max) | (-60.5, 483.0) | (-60.5, 54.0) | (-50.5, 483.0) |  |  |  |
| **IgG Actin Rabbit** |  |  |  | 0.42 | 0.752 |  |
| Mean (sd) | 5.2 (172.7) | -6.6 (78.0) | 29.9 (282.6) |  |  |  |
| Median (Q1,Q3) | -20.5 (-39.8, 1.0) | -24.0 (-42.0, 1.5) | -19.5 (-31.0, -5.5) |  |  |  |
| Range (min, max) | (-86.5, 1695.5) | (-86.5, 403.5) | (-63.5, 1695.5) |  |  |  |
| **IgG Aggrecan recombinant** |  |  |  | **0.05** | 0.337 |  |
| Mean (sd) | -12.7 (114.9) | -11.5 (138.0) | -15.2 (35.8) |  |  |  |
| Median (Q1,Q3) | -27.2 (-46.5, -6.2) | -32.5 (-52.5, -5.5) | -20 (-33, -12) |  |  |  |
| Range (min, max) | (-90, 1136) | (-90, 1136) | (-78.0, 103.5) |  |  |  |
| **IgG PBS** |  |  |  | 0.13 | 0.449 |  |
| Mean (sd) | -31.0 (53.5) | -31.4 (60.1) | -29.9 (37.0) |  |  |  |
| Median (Q1,Q3) | -39.0 (-53.5, -24.5) | -41.5 (-57.5, -26.0) | -32.5 (-44.5, -22.0) |  |  |  |
| Range (min, max) | (-84, 385) | (-84, 385) | (-80.5, 119.0) |  |  |  |
| **IgG Thyrogobulin** |  |  |  | 0.40 | 0.735 |  |
| Mean (sd) | 1150.5 (5708.4) | 1351.5 (6794.9) | 732.2 (2131.7) |  |  |  |
| Median (Q1,Q3) | 117.0 (78.5, 261.6) | 117.0 (78.5, 203.0) | 119.5 (88.0, 383.5) |  |  |  |
| Range (min, max) | (17.5, 42832.5) | (28.5, 42832.5) | (17.5, 11500.0) |  |  |  |
| **IgG BPI** |  |  |  | **0.05** | 0.327 |  |
| Mean (sd) | 278.4 (1251.1) | 362.9 (1510.7) | 102.5 (220.7) |  |  |  |
| Median (Q1,Q3) | 17.0 (-15.2, 71.1) | 7.5 (-26.5, 45.5) | 24.5 (12.0, 90.5) |  |  |  |
| Range (min, max) | (-73.5, 10230.0) | (-73.5, 10230.0) | (-46.5, 980.5) |  |  |  |
| **IgG SCGB1A1** |  |  |  | 0.54 | 0.823 |  |
| Mean (sd) | 16839.5 (19091.0) | 15991.4 (18798.0) | 18604.3 (19831.6) |  |  |  |
| Median (Q1,Q3) | 7914.2 (2043.4, 26421.4) | 7505.5 (2073.0, 24439.5) | 12677.5 (2033.5, 29182.5) |  |  |  |
| Range (min, max) | (219, 64063) | (266, 64057) | (219, 64063) |  |  |  |
| **IgG Bovin Histone subclass F1** |  |  |  | 0.24 | 0.621 |  |
| Mean (sd) | 1189.1 (1758.4) | 981.5 (1159.2) | 1621.0 (2566.1) |  |  |  |
| Median (Q1,Q3) | 617.5 (354.1, 1218.5) | 552 (306, 1111) | 684 (356, 1641) |  |  |  |
| Range (min, max) | (130, 13879) | (130, 6986) | (185.5, 13879.0) |  |  |  |
| **IgG Histone H2B** |  |  |  | 0.10 | 0.44 |  |
| Mean (sd) | 766.3 (1076.2) | 611.1 (601.0) | 1089.3 (1647.5) |  |  |  |
| Median (Q1,Q3) | 494.2 (283.8, 810.1) | 489.5 (270.5, 728.5) | 509.0 (320.0, 1010.5) |  |  |  |
| Range (min, max) | (106.5, 8550.5) | (106.5, 4477.5) | (194.5, 8550.5) |  |  |  |
| **IgG human IgG F ab 2** |  |  |  | 0.78 | 0.904 |  |
| Mean (sd) | 11669.3 (13798.4) | 12138.5 (14897.1) | 10692.9 (11299.4) |  |  |  |
| Median (Q1,Q3) | 6983.0 (2762.9, 15028.1) | 5534.5 (2011.5, 16677.5) | 7535.0 (3811.5, 12951.5) |  |  |  |
| Range (min, max) | (213.5, 64357.0) | (213.5, 64357.0) | (292.5, 50928.0) |  |  |  |
| **IgG HCEC memb** |  |  |  | **0.008** | 0.207 |  |
| Mean (sd) | 244.1 (203.2) | 213.4 (186.7) | 308.2 (223.2) |  |  |  |
| Median (Q1,Q3) | 185.2 (120.6, 274.9) | 166.5 (113.5, 234.0) | 207.0 (162.5, 377.5) |  |  |  |
| Range (min, max) | (-28.5, 1060.0) | (-28.5, 1060.0) | (-5.0, 953.5) |  |  |  |
| **IgG Glycyl tRNA Synthetase EJ** |  |  |  | **0.02** | 0.27 |  |
| Mean (sd) | 167.9 (832.1) | 89.2 (542.0) | 331.6 (1229.9) |  |  |  |
| Median (Q1,Q3) | 1.2 (-55.4, 76.0) | -15.5 (-61.0, 55.0) | 45 (-13, 157) |  |  |  |
| Range (min, max) | (-163.5, 7195.5) | (-163.5, 4494.5) | (-129.5, 7195.5) |  |  |  |
| **IgG Nup62** |  |  |  | 0.21 | 0.571 |  |
| Mean (sd) | 220.5 (669.6) | 198.1 (694.9) | 267.0 (620.0) |  |  |  |
| Median (Q1,Q3) | 6.0 (-48.2, 136.0) | 1.5 (-56.5, 115.0) | 15.0 (-18.5, 357.0) |  |  |  |
| Range (min, max) | (-149.5, 4195.0) | (-138.5, 4195.0) | (-149.5, 2749.5) |  |  |  |
| **IgG LCI** |  |  |  | **0.003** | 0.167 |  |
| Mean (sd) | 266.5 (699.3) | 167.9 (372.1) | 471.6 (1085.5) |  |  |  |
| Median (Q1,Q3) | 86.2 (-2.5, 251.0) | 48.5 (-14.0, 171.0) | 161.0 (62.5, 357.5) |  |  |  |
| Range (min, max) | (-140, 6149) | (-140, 1780) | (-50.5, 6149.0) |  |  |  |
| **IgG alphaB crystallin** |  |  |  | **0.04** | 0.321 |  |
| Mean (sd) | 328.5 (883.8) | 406.8 (1061.4) | 165.5 (182.6) |  |  |  |
| Median (Q1,Q3) | 131.8 (79.0, 221.2) | 144.5 (93.0, 238.5) | 104.5 (50.5, 208.5) |  |  |  |
| Range (min, max) | (-1.5, 7327.5) | (-1.5, 7327.5) | (14, 772) |  |  |  |
| **IgG Beta 2 Glyc recombinant human** |  |  |  | 0.27 | 0.645 |  |
| Mean (sd) | 164.5 (617.5) | 179.5 (734.4) | 133.4 (238.3) |  |  |  |
| Median (Q1,Q3) | 44.5 (10.5, 121.4) | 43.5 (8.5, 119.5) | 60.0 (17.5, 140.0) |  |  |  |
| Range (min, max) | (-75.0, 6357.5) | (-75.0, 6357.5) | (-16, 1278) |  |  |  |
| **IgG Ox LDL** |  |  |  | 0.09 | 0.414 |  |
| Mean (sd) | 211.1 (75.6) | 205.8 (82.1) | 222.2 (59.3) |  |  |  |
| Median (Q1,Q3) | 203.5 (148.9, 254.2) | 192.5 (145.5, 247.0) | 225.5 (195.0, 256.0) |  |  |  |
| Range (min, max) | (30.5, 529.0) | (30.5, 529.0) | (109.0, 325.5) |  |  |  |
| **IgG Proteoglycan** |  |  |  | 0.87 | 0.947 |  |
| Mean (sd) | -8.0 (48.1) | -7.8 (49.8) | -8.4 (44.9) |  |  |  |
| Median (Q1,Q3) | -21.2 (-40.9, 20.2) | -22.0 (-44.5, 20.5) | -20.5 (-39.0, 14.0) |  |  |  |
| Range (min, max) | (-76.0, 155.5) | (-76.0, 155.5) | (-67.0, 134.5) |  |  |  |
| **IgG LG3** |  |  |  | 0.14 | 0.449 |  |
| Mean (sd) | 164.3 (325.3) | 195.1 (372.2) | 100.2 (183.0) |  |  |  |
| Median (Q1,Q3) | 47.0 (1.4, 169.6) | 48.5 (4.0, 226.5) | 37.0 (-6.5, 122.0) |  |  |  |
| Range (min, max) | (-27.5, 1683.5) | (-27.5, 1683.5) | (-27.5, 937.5) |  |  |  |
| **IgG Ribo P2** |  |  |  | 0.39 | 0.723 |  |
| Mean (sd) | 193.3 (618.8) | 221.9 (749.5) | 134.0 (102.8) |  |  |  |
| Median (Q1,Q3) | 97.0 (43.0, 183.6) | 90.0 (43.0, 176.5) | 114.5 (38.0, 187.0) |  |  |  |
| Range (min, max) | (-63.5, 6381.5) | (-63.5, 6381.5) | (7.0, 395.5) |  |  |  |
| **IgG SmD2** |  |  |  | 0.27 | 0.645 |  |
| Mean (sd) | 162.1 (262.9) | 167.9 (303.8) | 150.0 (148.0) |  |  |  |
| Median (Q1,Q3) | 92.8 (43.0, 168.1) | 91.5 (38.0, 155.0) | 117.0 (58.0, 175.5) |  |  |  |
| Range (min, max) | (-78.0, 2318.5) | (-78.0, 2318.5) | (-63.5, 606.0) |  |  |  |
| **IgG PR3** |  |  |  | 0.85 | 0.944 |  |
| Mean (sd) | 97.0 (193.5) | 102.6 (199.3) | 85.5 (183.1) |  |  |  |
| Median (Q1,Q3) | 34.8 (5.6, 87.6) | 37.5 (5.0, 104.5) | 33.0 (11.5, 71.0) |  |  |  |
| Range (min, max) | (-34.5, 1016.0) | (-34.5, 1016.0) | (-14, 871) |  |  |  |
| **IgG snRNP C** |  |  |  | **0.01** | 0.224 |  |
| Mean (sd) | 1035.8 (2691.2) | 1003.8 (3172.4) | 1102.4 (1216.8) |  |  |  |
| Median (Q1,Q3) | 430.2 (254.2, 1054.8) | 397.5 (227.0, 774.0) | 657.0 (376.5, 1403.0) |  |  |  |
| Range (min, max) | (58, 27598) | (58, 27598) | (106.0, 5852.5) |  |  |  |
| **IgG snRNP 68 B B** |  |  |  | 0.25 | 0.629 |  |
| Mean (sd) | 849.1 (1697.1) | 825.4 (1836.9) | 898.5 (1383.1) |  |  |  |
| Median (Q1,Q3) | 410.2 (226.4, 749.5) | 389.5 (208.0, 753.0) | 451 (318, 733) |  |  |  |
| Range (min, max) | (76, 14383) | (76, 14383) | (80.5, 7672.5) |  |  |  |
| **IgG Mucarinic Rceptor 3** |  |  |  | **<0.001** | 0.078 |  |
| Mean (sd) | 126.0 (720.2) | 110.0 (851.0) | 159.3 (312.4) |  |  |  |
| Median (Q1,Q3) | 0.8 (-20.4, 45.0) | -10 (-24, 26) | 17.0 (-1.0, 189.5) |  |  |  |
| Range (min, max) | (-66.5, 7451.5) | (-66.5, 7451.5) | (-35.5, 1081.0) |  |  |  |
| **IgG Bovine Histone H3** |  |  |  | **0.03** | 0.281 |  |
| Mean (sd) | 458.2 (790.0) | 331.4 (419.3) | 722.0 (1217.3) |  |  |  |
| Median (Q1,Q3) | 250.0 (131.9, 434.4) | 227.0 (113.0, 400.5) | 284.5 (172.0, 654.5) |  |  |  |
| Range (min, max) | (17, 6679) | (17.0, 3131.5) | (80, 6679) |  |  |  |
| **IgG Histone H2A H2B dimers** |  |  |  | 0.13 | 0.449 |  |
| Mean (sd) | 482.1 (735.7) | 386.4 (467.0) | 681.2 (1085.5) |  |  |  |
| Median (Q1,Q3) | 250.0 (132.8, 503.1) | 222.5 (115.0, 447.5) | 293.0 (170.5, 643.0) |  |  |  |
| Range (min, max) | (44.5, 5541.5) | (44.5, 3185.5) | (73.0, 5541.5) |  |  |  |
| **IgG mouse IgG F ab 2** |  |  |  | 0.83 | 0.936 |  |
| Mean (sd) | 302.2 (874.2) | 326.6 (982.4) | 251.4 (598.0) |  |  |  |
| Median (Q1,Q3) | 66.5 (14.4, 232.9) | 71.0 (11.5, 232.5) | 57.5 (24.0, 264.0) |  |  |  |
| Range (min, max) | (-20.5, 6690.0) | (-14.5, 6690.0) | (-20.5, 3408.0) |  |  |  |
| **IgG human intestinal smooth muscle cell** |  |  |  | 0.06 | 0.366 |  |
| Mean (sd) | 301.9 (167.7) | 270.5 (116.4) | 367.4 (230.7) |  |  |  |
| Median (Q1,Q3) | 249.2 (202.6, 343.4) | 242.0 (196.5, 310.5) | 292.0 (212.0, 391.5) |  |  |  |
| Range (min, max) | (113.5, 1046.5) | (113.5, 851.0) | (133.5, 1046.5) |  |  |  |
| **IgG Intrinsic Factor** |  |  |  | 0.81 | 0.919 |  |
| Mean (sd) | 397.7 (636.7) | 352.5 (461.1) | 491.9 (900.0) |  |  |  |
| Median (Q1,Q3) | 206.5 (70.2, 451.8) | 204.5 (69.0, 410.5) | 208.5 (78.0, 460.5) |  |  |  |
| Range (min, max) | (-47.0, 5109.5) | (-47, 2297) | (-8.0, 5109.5) |  |  |  |
| **IgG RNP Sm non recombinant bovine** |  |  |  | **0.02** | 0.275 |  |
| Mean (sd) | 148.1 (468.4) | 154.0 (558.2) | 135.7 (174.5) |  |  |  |
| Median (Q1,Q3) | 44.8 (-23.0, 174.1) | 22.0 (-31.5, 164.0) | 87.5 (7.0, 196.0) |  |  |  |
| Range (min, max) | (-116.5, 4042.0) | (-116.5, 4042.0) | (-65, 740) |  |  |  |
| **IgG PDC E2** |  |  |  | **0.009** | 0.216 |  |
| Mean (sd) | 455.6 (935.7) | 448.6 (1040.6) | 470.2 (679.3) |  |  |  |
| Median (Q1,Q3) | 97.2 (6.1, 410.1) | 43.5 (-8.5, 308.5) | 172.0 (79.0, 527.5) |  |  |  |
| Range (min, max) | (-80.0, 5421.5) | (-80.0, 5421.5) | (-70.5, 2817.5) |  |  |  |
| **IgG dsDNA plasmid** |  |  |  | 0.31 | 0.677 |  |
| Mean (sd) | 395.3 (747.5) | 394.6 (841.1) | 396.8 (510.0) |  |  |  |
| Median (Q1,Q3) | 153.2 (53.1, 470.5) | 131.0 (45.5, 396.5) | 218.0 (77.0, 530.5) |  |  |  |
| Range (min, max) | (-27.0, 6052.5) | (-26.5, 6052.5) | (-27, 2253) |  |  |  |
| **IgG Measles** |  |  |  | 0.11 | 0.44 |  |
| Mean (sd) | 185.6 (224.7) | 171.9 (220.0) | 214.1 (234.6) |  |  |  |
| Median (Q1,Q3) | 106.0 (48.2, 259.2) | 100.5 (34.0, 233.0) | 130.0 (64.5, 328.5) |  |  |  |
| Range (min, max) | (-5.0, 1243.5) | (-5, 1151) | (21.0, 1243.5) |  |  |  |
| **IgG Laminin** |  |  |  | 0.34 | 0.689 |  |
| Mean (sd) | 11.8 (71.5) | 11.4 (78.6) | 12.5 (54.9) |  |  |  |
| Median (Q1,Q3) | -6.8 (-27.4, 25.6) | -12.5 (-27.5, 29.5) | 2.0 (-25.0, 24.5) |  |  |  |
| Range (min, max) | (-67.5, 452.5) | (-67.5, 452.5) | (-61.0, 199.5) |  |  |  |
| **IgG Collagen V C3657** |  |  |  | **<0.001** | 0.077 |  |
| Mean (sd) | 280.3 (1247.3) | 120.7 (125.5) | 612.4 (2163.9) |  |  |  |
| Median (Q1,Q3) | 92.2 (50.6, 221.8) | 78.5 (44.5, 172.5) | 160 (82, 329) |  |  |  |
| Range (min, max) | (-10.5, 13322.0) | (-10.5, 634.5) | (-6.5, 13322.0) |  |  |  |
| **IgG Porcine Myosin Heart** |  |  |  | 0.27 | 0.645 |  |
| Mean (sd) | 305.5 (581.1) | 273.1 (455.2) | 372.9 (784.7) |  |  |  |
| Median (Q1,Q3) | 123.0 (72.0, 272.4) | 112 (63, 250) | 124.0 (91.5, 345.5) |  |  |  |
| Range (min, max) | (-7, 4183) | (-7, 2517) | (-3.5, 4183.0) |  |  |  |
| **IgG TUBA1B protein** |  |  |  | 0.42 | 0.759 |  |
| Mean (sd) | -47.5 (17.6) | -48.4 (19.0) | -45.7 (14.2) |  |  |  |
| Median (Q1,Q3) | -49.0 (-57.0, -40.5) | -50.0 (-60.0, -40.5) | -47.5 (-53.5, -41.0) |  |  |  |
| Range (min, max) | (-88.5, 16.0) | (-88.5, 16.0) | (-69, -6) |  |  |  |
| **IgG Aldolase Type X** |  |  |  | 0.06 | 0.366 |  |
| Mean (sd) | -79.4 (40.3) | -84.8 (25.9) | -68.2 (59.0) |  |  |  |
| Median (Q1,Q3) | -79.8 (-101.9, -66.1) | -85.5 (-104.5, -67.5) | -72.5 (-93.0, -65.0) |  |  |  |
| Range (min, max) | (-137.5, 258.0) | (-137.5, -16.0) | (-121, 258) |  |  |  |
| **IgG TPO** |  |  |  | 0.13 | 0.449 |  |
| Mean (sd) | 1750.3 (4148.8) | 1374.3 (4008.9) | 2532.8 (4378.1) |  |  |  |
| Median (Q1,Q3) | 532.8 (136.4, 1318.5) | 457.5 (107.0, 1260.5) | 637.0 (271.5, 2711.0) |  |  |  |
| Range (min, max) | (-64, 33800) | (-64, 33800) | (0.0, 19181.5) |  |  |  |
| **IgG SRP54** |  |  |  | 0.13 | 0.449 |  |
| Mean (sd) | 993.7 (3043.4) | 916.0 (3228.7) | 1155.5 (2650.6) |  |  |  |
| Median (Q1,Q3) | 294.5 (130.4, 795.9) | 257.0 (106.5, 779.0) | 382.0 (214.0, 801.5) |  |  |  |
| Range (min, max) | (-31, 27944) | (-31, 27944) | (-10.5, 14414.5) |  |  |  |
| **IgG PL 7** |  |  |  | 0.70 | 0.877 |  |
| Mean (sd) | 558.2 (2555.0) | 734.9 (3095.9) | 190.4 (225.8) |  |  |  |
| Median (Q1,Q3) | 117.8 (18.5, 311.6) | 140.0 (16.5, 325.5) | 104.5 (29.5, 247.0) |  |  |  |
| Range (min, max) | (-79.5, 26511.0) | (-79.5, 26511.0) | (-62.5, 939.5) |  |  |  |
| **IgG human albumin** |  |  |  | 0.36 | 0.715 |  |
| Mean (sd) | 1047.8 (1449.3) | 1009.2 (1451.5) | 1128.3 (1461.3) |  |  |  |
| Median (Q1,Q3) | 418.5 (113.6, 1139.5) | 330.5 (98.5, 1136.5) | 485.5 (162.0, 1478.5) |  |  |  |
| Range (min, max) | (5.5, 5955.5) | (8.5, 5955.5) | (5.5, 5040.0) |  |  |  |
| **IgG C1q purified non recombinant** |  |  |  | 0.20 | 0.548 |  |
| Mean (sd) | 32.9 (53.5) | 28.1 (48.8) | 42.7 (61.7) |  |  |  |
| Median (Q1,Q3) | 14.5 (1.1, 41.2) | 12.5 (0.5, 33.5) | 22.5 (4.0, 53.0) |  |  |  |
| Range (min, max) | (-22, 284) | (-22, 284) | (-12.0, 279.5) |  |  |  |
| **IgG Human LEDGF** |  |  |  | 0.97 | 0.981 |  |
| Mean (sd) | 1368.3 (1542.7) | 1509.6 (1784.5) | 1074.1 (784.6) |  |  |  |
| Median (Q1,Q3) | 873.0 (420.5, 1701.1) | 823.5 (392.0, 1964.5) | 980.5 (507.0, 1242.0) |  |  |  |
| Range (min, max) | (115.0, 10141.5) | (115.0, 10141.5) | (179.0, 3808.5) |  |  |  |
| **IgG mouse IgG Fc** |  |  |  | 0.47 | 0.786 |  |
| Mean (sd) | 194.5 (239.1) | 206.6 (250.2) | 169.4 (215.1) |  |  |  |
| Median (Q1,Q3) | 103.0 (66.2, 212.6) | 119.0 (67.0, 217.5) | 97 (66, 165) |  |  |  |
| Range (min, max) | (-13.5, 1215.5) | (-13.5, 1215.5) | (-1.5, 1103.5) |  |  |  |
| **IgG Troponin I** |  |  |  | 0.96 | 0.98 |  |
| Mean (sd) | 979.5 (2098.2) | 1068.5 (2255.7) | 794.2 (1739.4) |  |  |  |
| Median (Q1,Q3) | 311.8 (119.4, 764.1) | 306 (116, 735) | 423.0 (129.5, 768.5) |  |  |  |
| Range (min, max) | (16.0, 13182.5) | (16.0, 13182.5) | (28.5, 10521.0) |  |  |  |
| **IgG beta 2 GPI non recombinant Bovine** |  |  |  | 0.11 | 0.44 |  |
| Mean (sd) | -24.2 (54.5) | -26.7 (59.4) | -18.8 (42.8) |  |  |  |
| Median (Q1,Q3) | -30.8 (-60.5, -7.2) | -35.0 (-62.5, -10.5) | -19.5 (-46.0, 1.5) |  |  |  |
| Range (min, max) | (-134.0, 267.5) | (-134.0, 267.5) | (-99.5, 95.5) |  |  |  |
| **IgG Ro SS A 60 kD non recombinant bovine** |  |  |  | 0.06 | 0.36 |  |
| Mean (sd) | 575.2 (3197.6) | 606.2 (3605.1) | 510.6 (2156.3) |  |  |  |
| Median (Q1,Q3) | 44.8 (3.9, 140.1) | 43.0 (-3.5, 127.0) | 50.0 (16.5, 184.5) |  |  |  |
| Range (min, max) | (-71, 29926) | (-71, 29926) | (-8.0, 13181.5) |  |  |  |
| **IgG M2** |  |  |  | **0.01** | 0.235 |  |
| Mean (sd) | 275.4 (615.0) | 260.7 (690.6) | 306.1 (423.0) |  |  |  |
| Median (Q1,Q3) | 59.2 (-17.5, 342.6) | 24.0 (-22.5, 251.5) | 169.0 (53.5, 424.5) |  |  |  |
| Range (min, max) | (-104.5, 4984.0) | (-104.5, 4984.0) | (-71.5, 2108.0) |  |  |  |
| **IgG M2AR** |  |  |  | 0.71 | 0.879 |  |
| Mean (sd) | -5.9 (12.0) | -6.4 (10.1) | -4.8 (15.3) |  |  |  |
| Median (Q1,Q3) | -7.5 (-12.9, 0.9) | -6.0 (-12.5, 1.0) | -9 (-13, -2) |  |  |  |
| Range (min, max) | (-29.5, 57.5) | (-26.5, 22.5) | (-29.5, 57.5) |  |  |  |
| **IgG A1AR** |  |  |  | 0.89 | 0.959 |  |
| Mean (sd) | -5.1 (178.6) | 7.5 (216.5) | -31.3 (12.8) |  |  |  |
| Median (Q1,Q3) | -33.0 (-42.5, -21.6) | -33 (-44, -21) | -33 (-38, -27) |  |  |  |
| Range (min, max) | (-56.5, 1746.0) | (-56.5, 1746.0) | (-56, 6) |  |  |  |
| **IgG Troponin C** |  |  |  | 0.33 | 0.689 |  |
| Mean (sd) | 777.2 (829.8) | 821.3 (826.9) | 685.5 (839.6) |  |  |  |
| Median (Q1,Q3) | 467.8 (227.1, 1002.2) | 512.0 (284.5, 1123.5) | 387 (226, 807) |  |  |  |
| Range (min, max) | (-8.5, 4669.0) | (-8.5, 4459.5) | (99.5, 4669.0) |  |  |  |
| **IgG HSP 47** |  |  |  | 0.24 | 0.614 |  |
| Mean (sd) | 246.9 (674.9) | 205.6 (428.5) | 333.0 (1015.4) |  |  |  |
| Median (Q1,Q3) | 60.5 (9.0, 145.2) | 67.5 (18.0, 122.5) | 29.5 (-8.5, 184.0) |  |  |  |
| Range (min, max) | (-26.0, 5946.5) | (-24.0, 2626.5) | (-26.0, 5946.5) |  |  |  |
| **IgG alpha Actinin** |  |  |  | 0.17 | 0.511 |  |
| Mean (sd) | 1.5 (8.9) | 0.2 (7.1) | 4.0 (11.4) |  |  |  |
| Median (Q1,Q3) | 0.5 (-4.5, 5.0) | 0.0 (-4.5, 4.0) | 1.5 (-5.0, 9.0) |  |  |  |
| Range (min, max) | (-11.5, 42.0) | (-11.5, 28.5) | (-11, 42) |  |  |  |
| **IgG Alpha KGDH** |  |  |  | **0.006** | 0.207 |  |
| Mean (sd) | 43.2 (103.8) | 36.5 (115.6) | 57.3 (73.1) |  |  |  |
| Median (Q1,Q3) | 12.5 (-2.8, 48.9) | 5.5 (-4.5, 36.0) | 27.0 (5.5, 79.0) |  |  |  |
| Range (min, max) | (-30.5, 916.5) | (-30.5, 916.5) | (-21.5, 250.0) |  |  |  |
| **IgG Fib IV** |  |  |  | 0.07 | 0.389 |  |
| Mean (sd) | -30.4 (45.1) | -33.0 (44.8) | -25.1 (45.7) |  |  |  |
| Median (Q1,Q3) | -43.0 (-57.1, -23.1) | -45.5 (-59.0, -26.0) | -37.5 (-47.5, -17.5) |  |  |  |
| Range (min, max) | (-77.0, 211.5) | (-77.0, 211.5) | (-73.0, 139.5) |  |  |  |
| **IgG PCNA** |  |  |  | 0.13 | 0.449 |  |
| Mean (sd) | 247.2 (659.7) | 208.0 (546.9) | 328.6 (851.4) |  |  |  |
| Median (Q1,Q3) | 37.0 (-2.5, 148.8) | 36.5 (-12.5, 125.0) | 39.5 (19.0, 175.5) |  |  |  |
| Range (min, max) | (-79.5, 3983.5) | (-79.5, 3490.5) | (-72.5, 3983.5) |  |  |  |
| **IgG snRNP A** |  |  |  | 0.73 | 0.889 |  |
| Mean (sd) | 602.2 (1685.6) | 701.9 (1906.5) | 394.7 (1085.9) |  |  |  |
| Median (Q1,Q3) | 130.8 (27.9, 315.2) | 135.0 (26.0, 539.5) | 98.5 (37.5, 265.5) |  |  |  |
| Range (min, max) | (-84.5, 12387.0) | (-84.5, 12387.0) | (-36.0, 5913.5) |  |  |  |
| **IgG PM Scl 75** |  |  |  | **0.005** | 0.207 |  |
| Mean (sd) | 621.8 (1539.5) | 541.9 (1468.8) | 788.1 (1686.1) |  |  |  |
| Median (Q1,Q3) | 119.8 (-87.2, 602.0) | 28.5 (-128.5, 528.0) | 262.0 (90.0, 884.5) |  |  |  |
| Range (min, max) | (-267.5, 8846.0) | (-267.5, 8586.0) | (-149.5, 8846.0) |  |  |  |
| **IgG human C1q Abcam** |  |  |  | **0.03** | 0.281 |  |
| Mean (sd) | 9484.7 (2035.4) | 9281.2 (2112.9) | 9908.3 (1818.2) |  |  |  |
| Median (Q1,Q3) | 9249.2 (8054.1, 10497.2) | 8859.0 (7658.5, 9909.5) | 9983.0 (8758.5, 11065.5) |  |  |  |
| Range (min, max) | (4880, 15618) | (4880, 15618) | (6567.0, 15240.5) |  |  |  |
| **IgG whole histones** |  |  |  | **0.02** | 0.252 |  |
| Mean (sd) | 869.9 (1172.9) | 678.7 (674.5) | 1267.7 (1765.5) |  |  |  |
| Median (Q1,Q3) | 501.8 (378.6, 872.0) | 465.0 (375.0, 791.5) | 640.5 (411.0, 1316.0) |  |  |  |
| Range (min, max) | (146.5, 9880.0) | (146.5, 5013.0) | (265.5, 9880.0) |  |  |  |
| **IgG human nucleosome** |  |  |  | 0.91 | 0.959 |  |
| Mean (sd) | 763.7 (711.4) | 795.8 (797.2) | 696.9 (490.0) |  |  |  |
| Median (Q1,Q3) | 493.5 (398.2, 787.4) | 490.0 (399.0, 761.5) | 533.5 (390.0, 877.5) |  |  |  |
| Range (min, max) | (134, 4921) | (134, 4921) | (184.5, 2692.5) |  |  |  |
| **IgG human IgG Fc** |  |  |  | **0.03** | 0.281 |  |
| Mean (sd) | 53402.3 (8991.3) | 54725.6 (8391.0) | 50648.4 (9673.7) |  |  |  |
| Median (Q1,Q3) | 54048.5 (45796.6, 63693.5) | 56503.5 (47900.0, 63899.0) | 48260.5 (44059.5, 60203.5) |  |  |  |
| Range (min, max) | (34506, 64488) | (39820.5, 64488.0) | (34506, 64236) |  |  |  |
| **IgG HCEC total Triton** |  |  |  | 0.53 | 0.823 |  |
| Mean (sd) | 1623.3 (4557.0) | 2001.1 (5511.8) | 837.1 (326.2) |  |  |  |
| Median (Q1,Q3) | 769.0 (562.6, 1001.9) | 739.5 (533.5, 998.5) | 776.5 (617.0, 1020.5) |  |  |  |
| Range (min, max) | (222, 36439) | (222, 36439) | (344.5, 1559.0) |  |  |  |
| **IgG TIF1 gamma** |  |  |  | 0.11 | 0.44 |  |
| Mean (sd) | 121.5 (315.6) | 91.5 (257.5) | 184.1 (408.2) |  |  |  |
| Median (Q1,Q3) | 2.8 (-44.2, 114.9) | -6.5 (-51.0, 114.5) | 25.5 (-21.5, 120.5) |  |  |  |
| Range (min, max) | (-123.5, 1751.0) | (-123.5, 1284.0) | (-78.5, 1751.0) |  |  |  |
| **IgG gp210** |  |  |  | 0.61 | 0.85 |  |
| Mean (sd) | 578.9 (968.5) | 536.9 (898.6) | 666.4 (1108.1) |  |  |  |
| Median (Q1,Q3) | 276.2 (92.0, 673.6) | 240 (84, 656) | 304.5 (144.5, 729.0) |  |  |  |
| Range (min, max) | (-134.5, 6158.5) | (-116.5, 6158.5) | (-134.5, 5798.0) |  |  |  |
| **IgG GP2** |  |  |  | 0.15 | 0.48 |  |
| Mean (sd) | 86.4 (414.9) | 105.4 (497.4) | 46.6 (125.5) |  |  |  |
| Median (Q1,Q3) | -3.8 (-32.8, 49.4) | -8.0 (-40.5, 68.0) | 8.0 (-13.5, 49.0) |  |  |  |
| Range (min, max) | (-74.0, 4122.5) | (-74.0, 4122.5) | (-67.0, 606.5) |  |  |  |
| **IgG HSP 90** |  |  |  | 0.66 | 0.852 |  |
| Mean (sd) | 17.5 (75.3) | 22.9 (90.7) | 6.3 (16.4) |  |  |  |
| Median (Q1,Q3) | 3.8 (-11.5, 18.2) | 4.0 (-13.0, 17.5) | 3.0 (-4.5, 18.5) |  |  |  |
| Range (min, max) | (-31.5, 566.0) | (-31.5, 566.0) | (-18.0, 46.5) |  |  |  |
| **IgG HMG CoA** |  |  |  | 0.21 | 0.57 |  |
| Mean (sd) | 832.5 (2055.8) | 636.1 (1196.5) | 1241.2 (3160.5) |  |  |  |
| Median (Q1,Q3) | 268.8 (131.2, 524.0) | 255.5 (123.5, 445.5) | 293.5 (139.5, 639.5) |  |  |  |
| Range (min, max) | (34, 17078) | (34, 8089) | (78, 17078) |  |  |  |
| **IgG Collagen I C7774** |  |  |  | **0.01** | 0.224 |  |
| Mean (sd) | 182.8 (179.7) | 159.8 (174.5) | 230.9 (183.0) |  |  |  |
| Median (Q1,Q3) | 116.5 (50.5, 263.4) | 101.5 (42.0, 239.5) | 196.5 (84.0, 323.0) |  |  |  |
| Range (min, max) | (-6.5, 997.5) | (-6.5, 997.5) | (3.5, 786.0) |  |  |  |
| **IgG dsDNA genomic** |  |  |  | 0.12 | 0.449 |  |
| Mean (sd) | 967.7 (1204.2) | 931.7 (1301.4) | 1042.7 (983.8) |  |  |  |
| Median (Q1,Q3) | 552.8 (231.0, 1212.6) | 505.5 (204.5, 1156.5) | 754.5 (377.5, 1293.5) |  |  |  |
| Range (min, max) | (6.0, 8354.5) | (6.0, 8354.5) | (40, 4179) |  |  |  |
| **IgG PDH** |  |  |  | **0.02** | 0.277 |  |
| Mean (sd) | 138.8 (128.6) | 123.9 (125.5) | 169.6 (131.1) |  |  |  |
| Median (Q1,Q3) | 85.5 (54.1, 171.9) | 79.5 (48.5, 127.0) | 129.0 (66.5, 238.0) |  |  |  |
| Range (min, max) | (13.5, 704.5) | (13.5, 704.5) | (15, 483) |  |  |  |
| **IgG SmD1** |  |  |  | 0.07 | 0.39 |  |
| Mean (sd) | 461.2 (1035.9) | 506.5 (1211.1) | 367.0 (508.5) |  |  |  |
| Median (Q1,Q3) | 82.8 (-4.5, 340.2) | 49.5 (-9.0, 305.0) | 149.5 (36.5, 441.0) |  |  |  |
| Range (min, max) | (-65.5, 6156.5) | (-65.5, 6156.5) | (-38.5, 1688.0) |  |  |  |
| **IgG SmD** |  |  |  | **0.04** | 0.321 |  |
| Mean (sd) | 442.9 (845.6) | 419.5 (922.8) | 491.6 (665.8) |  |  |  |
| Median (Q1,Q3) | 142.0 (5.1, 440.0) | 106.5 (-5.5, 350.0) | 197.5 (85.5, 516.5) |  |  |  |
| Range (min, max) | (-110.0, 5478.5) | (-110.0, 5478.5) | (-62, 2768) |  |  |  |
| **IgG Mi 2** |  |  |  | 0.11 | 0.44 |  |
| Mean (sd) | 445.4 (1126.4) | 340.1 (704.6) | 664.6 (1691.6) |  |  |  |
| Median (Q1,Q3) | 135.5 (57.0, 404.2) | 129.5 (55.5, 324.5) | 208.5 (84.0, 547.0) |  |  |  |
| Range (min, max) | (-72.5, 10247.5) | (-70.0, 5359.5) | (-72.5, 10247.5) |  |  |  |
| **IgG Ro SS A 52 kDa human recombinant** |  |  |  | 0.25 | 0.633 |  |
| Mean (sd) | 2129.5 (8645.3) | 2625.4 (10242.6) | 1097.6 (3391.8) |  |  |  |
| Median (Q1,Q3) | 27.5 (-4.0, 195.1) | 23.0 (-10.5, 188.0) | 41.0 (12.5, 222.0) |  |  |  |
| Range (min, max) | (-45.5, 64352.0) | (-45.5, 64352.0) | (-26.0, 15845.5) |  |  |  |
| **IgG CENP A** |  |  |  | 0.06 | 0.366 |  |
| Mean (sd) | 238.9 (715.0) | 149.7 (285.6) | 424.6 (1174.8) |  |  |  |
| Median (Q1,Q3) | 89.8 (42.0, 222.4) | 77.5 (40.5, 179.0) | 113.5 (65.5, 308.0) |  |  |  |
| Range (min, max) | (-273, 6956) | (-273.0, 2297.5) | (-13, 6956) |  |  |  |
| **IgG Carbonic Anhydrase** |  |  |  | 0.54 | 0.823 |  |
| Mean (sd) | 1548.3 (1131.0) | 1507.5 (1104.2) | 1633.3 (1196.0) |  |  |  |
| Median (Q1,Q3) | 1300.2 (901.2, 1815.8) | 1242.0 (779.5, 1842.5) | 1373.0 (1001.5, 1616.5) |  |  |  |
| Range (min, max) | (104.5, 6690.5) | (104.5, 6690.5) | (327, 6654) |  |  |  |
| **IgG Bovine Histone H2b F2b** |  |  |  | **<0.001** | 0.077 |  |
| Mean (sd) | 388.0 (608.9) | 302.5 (503.3) | 566.1 (762.1) |  |  |  |
| Median (Q1,Q3) | 202.2 (117.1, 393.0) | 173.0 (104.5, 264.0) | 350.5 (187.5, 611.0) |  |  |  |
| Range (min, max) | (37.5, 3935.0) | (37.5, 3748.0) | (55.5, 3935.0) |  |  |  |
| **IgG Recombinant Histone H2A hu** |  |  |  | 0.05 | 0.359 |  |
| Mean (sd) | 764.6 (714.4) | 674.8 (639.8) | 951.4 (827.4) |  |  |  |
| Median (Q1,Q3) | 524.0 (318.4, 933.8) | 410.5 (303.0, 819.0) | 587.5 (370.0, 1064.5) |  |  |  |
| Range (min, max) | (34, 3277) | (34, 3277) | (158.5, 2874.0) |  |  |  |
| **IgG Human IgM** |  |  |  | 0.51 | 0.813 |  |
| Mean (sd) | 250.1 (389.9) | 276.6 (456.6) | 194.9 (180.0) |  |  |  |
| Median (Q1,Q3) | 161.8 (58.9, 382.2) | 267.5 (52.5, 383.5) | 82.5 (63.5, 381.5) |  |  |  |
| Range (min, max) | (-5.5, 3969.5) | (-5.5, 3969.5) | (25.5, 575.0) |  |  |  |
| **IgG HCEC total SDS** |  |  |  | 0.11 | 0.44 |  |
| Mean (sd) | 748.6 (766.9) | 745.1 (880.2) | 755.8 (458.5) |  |  |  |
| Median (Q1,Q3) | 574.5 (362.2, 849.4) | 545 (339, 802) | 652 (410, 981) |  |  |  |
| Range (min, max) | (159, 6719) | (159, 6719) | (213.5, 2592.0) |  |  |  |
| **IgG Asparaginyl tRNA Synthetase** |  |  |  | 0.48 | 0.795 |  |
| Mean (sd) | 335.3 (956.4) | 416.3 (1139.8) | 166.6 (292.2) |  |  |  |
| Median (Q1,Q3) | 83.2 (-7.5, 299.6) | 100.5 (-16.5, 414.0) | 52.5 (-6.0, 271.0) |  |  |  |
| Range (min, max) | (-95.0, 7704.5) | (-95.0, 7704.5) | (-45.5, 1535.0) |  |  |  |
| **IgG LKM 1 hp** |  |  |  | 0.38 | 0.717 |  |
| Mean (sd) | 74.0 (132.3) | 64.3 (130.3) | 94.3 (135.9) |  |  |  |
| Median (Q1,Q3) | 30.5 (10.1, 68.9) | 27.5 (10.5, 66.5) | 35.0 (10.0, 75.5) |  |  |  |
| Range (min, max) | (-25.5, 998.0) | (-25.5, 998.0) | (-16.5, 461.5) |  |  |  |
| **IgG tTG baculovirus** |  |  |  | 0.24 | 0.614 |  |
| Mean (sd) | 74.5 (344.6) | 76.2 (371.5) | 70.9 (285.5) |  |  |  |
| Median (Q1,Q3) | -31.5 (-65.4, 56.0) | -37.0 (-69.5, 57.0) | -16 (-49, 53) |  |  |  |
| Range (min, max) | (-111, 2829) | (-111, 2829) | (-99.5, 1509.0) |  |  |  |
| **IgG Vimentin** |  |  |  | 0.09 | 0.414 |  |
| Mean (sd) | -42.6 (19.0) | -43.8 (20.6) | -40.2 (15.2) |  |  |  |
| Median (Q1,Q3) | -44.0 (-56.1, -33.1) | -46.0 (-59.0, -36.5) | -40.0 (-49.5, -30.0) |  |  |  |
| Range (min, max) | (-76.0, 47.5) | (-76.0, 47.5) | (-72.5, -6.0) |  |  |  |
| **IgG Alpha elastin** |  |  |  | 0.35 | 0.694 |  |
| Mean (sd) | 89.6 (276.4) | 98.8 (315.8) | 70.5 (169.2) |  |  |  |
| Median (Q1,Q3) | -0.2 (-21.1, 65.8) | -2.0 (-25.0, 85.5) | 4.5 (-15.5, 53.5) |  |  |  |
| Range (min, max) | (-54.5, 2013.0) | (-54.5, 2013.0) | (-52.5, 795.0) |  |  |  |
| **IgG Troponin T** |  |  |  | 0.70 | 0.877 |  |
| Mean (sd) | 1030.5 (1905.8) | 1011.3 (1722.3) | 1070.4 (2266.2) |  |  |  |
| Median (Q1,Q3) | 477.0 (282.2, 942.8) | 514.0 (271.5, 1053.5) | 407.0 (304.5, 709.0) |  |  |  |
| Range (min, max) | (85, 13221) | (85.0, 12558.5) | (104, 13221) |  |  |  |
| **IgG Collagen IV C5533** |  |  |  | 0.07 | 0.366 |  |
| Mean (sd) | 21.8 (48.5) | 17.2 (43.2) | 31.2 (57.6) |  |  |  |
| Median (Q1,Q3) | 10.5 (-7.4, 37.9) | 7.0 (-11.5, 35.5) | 18.5 (4.5, 42.5) |  |  |  |
| Range (min, max) | (-49.5, 314.0) | (-49.5, 169.0) | (-34, 314) |  |  |  |
| **IgG GBM diss** |  |  |  | 0.21 | 0.581 |  |
| Mean (sd) | -33.3 (90.8) | -29.7 (90.7) | -40.9 (91.7) |  |  |  |
| Median (Q1,Q3) | -34.5 (-70.0, 1.0) | -28.5 (-66.0, 1.5) | -52.0 (-85.0, -2.5) |  |  |  |
| Range (min, max) | (-351.5, 390.5) | (-351.5, 298.5) | (-185.0, 390.5) |  |  |  |
| **IgG Cardiolipin C1649** |  |  |  | 0.83 | 0.936 |  |
| Mean (sd) | 103.0 (200.7) | 97.0 (176.8) | 115.6 (245.3) |  |  |  |
| Median (Q1,Q3) | 37.0 (0.8, 116.0) | 40.5 (2.5, 116.0) | 36.0 (0.5, 116.0) |  |  |  |
| Range (min, max) | (-60, 1127) | (-60.0, 1095.5) | (-53.5, 1127.0) |  |  |  |
| **IgG Collagen VI C7521** |  |  |  | 0.58 | 0.839 |  |
| Mean (sd) | 2.9 (42.1) | 4.1 (47.7) | 0.4 (27.4) |  |  |  |
| Median (Q1,Q3) | -5.5 (-17.4, 7.6) | -8.0 (-19.0, 10.5) | -4.5 (-13.0, 4.5) |  |  |  |
| Range (min, max) | (-53, 241) | (-51, 241) | (-53.0, 90.5) |  |  |  |
| **IgG PM Scl 100** |  |  |  | 0.15 | 0.476 |  |
| Mean (sd) | 2202.4 (6105.8) | 2226.3 (6815.8) | 2152.8 (4352.6) |  |  |  |
| Median (Q1,Q3) | 697.5 (408.5, 1602.2) | 641.5 (395.5, 1275.5) | 832.5 (485.0, 1943.5) |  |  |  |
| Range (min, max) | (95.5, 46620.5) | (95.5, 46620.5) | (233.5, 26230.5) |  |  |  |
| **IgG snRNP 68** |  |  |  | 0.07 | 0.366 |  |
| Mean (sd) | 254.3 (766.7) | 271.0 (921.4) | 219.5 (226.0) |  |  |  |
| Median (Q1,Q3) | 99.8 (44.6, 239.6) | 75.5 (36.5, 202.0) | 153.0 (67.5, 262.5) |  |  |  |
| Range (min, max) | (-28, 8019) | (-28, 8019) | (1.5, 963.0) |  |  |  |
| **IgG CENP B** |  |  |  | **0.003** | 0.182 |  |
| Mean (sd) | 785.2 (1844.6) | 574.7 (1500.9) | 1223.3 (2373.3) |  |  |  |
| Median (Q1,Q3) | 240.2 (65.5, 643.4) | 172.0 (24.5, 492.0) | 342.0 (197.0, 1947.5) |  |  |  |
| Range (min, max) | (-121, 13912) | (-121.0, 10900.5) | (-71, 13912) |  |  |  |
| **IgG Human IgE** |  |  |  | 0.66 | 0.852 |  |
| Mean (sd) | 785.2 (486.2) | 814.9 (552.0) | 723.4 (304.8) |  |  |  |
| Median (Q1,Q3) | 657.5 (501.5, 933.4) | 646.0 (501.5, 954.0) | 681 (490, 930) |  |  |  |
| Range (min, max) | (249, 4061) | (249, 4061) | (337.0, 1437.5) |  |  |  |
| **IgG La SS B Antigens Immunovision** |  |  |  | 0.95 | 0.978 |  |
| Mean (sd) | 1047.7 (1904.8) | 1201.9 (2163.0) | 727.0 (1163.9) |  |  |  |
| Median (Q1,Q3) | 414.2 (216.4, 978.6) | 394.5 (194.0, 1054.5) | 438 (245, 792) |  |  |  |
| Range (min, max) | (66.0, 9520.5) | (66.5, 9520.5) | (66.0, 7263.5) |  |  |  |
| IgG Histone H4 1 103 aa |  |  |  | **0.02** | 0.252 |  |
| Mean (sd) | 719.8 (841.5) | 571.9 (498.4) | 1027.6 (1246.7) |  |  |  |
| Median (Q1,Q3) | 461.2 (295.1, 877.0) | 426.0 (235.5, 790.0) | 638.5 (372.5, 1014.0) |  |  |  |
| Range (min, max) | (32.5, 6585.5) | (32.5, 3036.5) | (133.0, 6585.5) |  |  |  |
| **IgG whole mouse IgG** |  |  |  | 0.45 | 0.781 |  |
| Mean (sd) | 354.3 (549.7) | 313.5 (374.9) | 439.0 (800.4) |  |  |  |
| Median (Q1,Q3) | 176.0 (92.1, 395.8) | 169.5 (88.5, 338.5) | 212 (100, 473) |  |  |  |
| Range (min, max) | (22.0, 4794.5) | (22, 2338) | (41.0, 4794.5) |  |  |  |
| **IgG Desmin** |  |  |  | 0.26 | 0.645 |  |
| Mean (sd) | 127.7 (503.3) | 40.2 (175.8) | 309.6 (824.3) |  |  |  |
| Median (Q1,Q3) | -14.5 (-63.5, 133.6) | -14.0 (-60.5, 76.0) | -17.0 (-64.5, 313.0) |  |  |  |
| Range (min, max) | (-156.0, 3543.5) | (-156.0, 772.5) | (-106.0, 3543.5) |  |  |  |
| **IgG beta 2 GPI non recombinant Human** |  |  |  | **0.007** | 0.207 |  |
| Mean (sd) | 4.3 (37.0) | -1.5 (36.4) | 16.4 (35.9) |  |  |  |
| Median (Q1,Q3) | -1.0 (-15.0, 22.9) | -5.0 (-22.5, 14.0) | 14.0 (-7.5, 35.5) |  |  |  |
| Range (min, max) | (-70, 138) | (-70, 138) | (-50.0, 110.5) |  |  |  |
| **IgG Nucleosome non recombinant bovine** |  |  |  | **0.006** | 0.207 |  |
| Mean (sd) | 300.5 (414.4) | 242.0 (366.9) | 422.3 (482.0) |  |  |  |
| Median (Q1,Q3) | 160.5 (64.0, 339.8) | 119.5 (43.0, 289.5) | 231.0 (109.5, 393.5) |  |  |  |
| Range (min, max) | (-23.5, 2406.0) | (-23.5, 2406.0) | (-14.5, 1927.5) |  |  |  |
| **IgG BCOADC E2** |  |  |  | **0.04** | 0.314 |  |
| Mean (sd) | 260.5 (543.9) | 255.3 (623.7) | 271.3 (327.4) |  |  |  |
| Median (Q1,Q3) | 99.5 (16.6, 275.2) | 77.0 (12.0, 226.5) | 162.0 (48.0, 416.5) |  |  |  |
| Range (min, max) | (-42.0, 4081.5) | (-42.0, 4081.5) | (-27.5, 1445.0) |  |  |  |
| **IgG human fgl2** |  |  |  | 0.19 | 0.532 |  |
| Mean (sd) | 236.5 (129.2) | 224.2 (105.1) | 262.1 (167.6) |  |  |  |
| Median (Q1,Q3) | 201.2 (172.0, 245.0) | 197.5 (161.5, 241.5) | 211.5 (180.0, 261.0) |  |  |  |
| Range (min, max) | (77.5, 972.5) | (77.5, 735.0) | (119.5, 972.5) |  |  |  |
| **IgG B1AR** |  |  |  | 0.27 | 0.645 |  |
| Mean (sd) | 34.4 (196.7) | 23.6 (165.1) | 56.9 (251.2) |  |  |  |
| Median (Q1,Q3) | -13.5 (-27.4, 3.4) | -15.5 (-29.0, 0.0) | -10.5 (-23.5, 5.0) |  |  |  |
| Range (min, max) | (-56, 1362) | (-51.0, 1243.5) | (-56, 1362) |  |  |  |
| **IgG Grp78 BiP** |  |  |  | 0.17 | 0.511 |  |
| Mean (sd) | 1044.5 (2227.2) | 1132.0 (2673.6) | 862.3 (655.1) |  |  |  |
| Median (Q1,Q3) | 605.2 (278.6, 956.5) | 493.5 (286.5, 850.0) | 771.5 (271.0, 1050.5) |  |  |  |
| Range (min, max) | (19.5, 22638.0) | (19.5, 22638.0) | (85.5, 2917.0) |  |  |  |
| **IgG HSP 40** |  |  |  | 0.86 | 0.947 |  |
| Mean (sd) | 397.0 (1756.5) | 281.1 (512.6) | 638.3 (3006.8) |  |  |  |
| Median (Q1,Q3) | 78.0 (11.2, 261.8) | 53.0 (10.0, 276.5) | 99.5 (22.0, 219.0) |  |  |  |
| Range (min, max) | (-39.5, 18404.0) | (-39.5, 2716.0) | (-23.5, 18404.0) |  |  |  |
| **IgG Enolase** |  |  |  | 0.27 | 0.645 |  |
| Mean (sd) | 202.7 (450.9) | 176.3 (413.0) | 257.8 (523.1) |  |  |  |
| Median (Q1,Q3) | 73.8 (46.8, 146.8) | 72.0 (45.5, 143.0) | 88.5 (54.5, 174.5) |  |  |  |
| Range (min, max) | (9.0, 3289.5) | (9.0, 3289.5) | (18.0, 2905.5) |  |  |  |
| **IgG ssDNA** |  |  |  | 0.26 | 0.645 |  |
| Mean (sd) | 1898.9 (2160.4) | 1892.8 (2379.1) | 1911.6 (1643.5) |  |  |  |
| Median (Q1,Q3) | 1146.0 (656.9, 2160.5) | 1061 (638, 1893) | 1374 (772, 2407) |  |  |  |
| Range (min, max) | (120, 13610) | (120, 13610) | (180, 7348) |  |  |  |
| **IgG Fib I** |  |  |  | 0.81 | 0.919 |  |
| Mean (sd) | 812.3 (1739.2) | 637.2 (288.4) | 1176.7 (3019.5) |  |  |  |
| Median (Q1,Q3) | 594.5 (427.5, 741.6) | 612.0 (425.5, 743.5) | 577.0 (440.5, 732.0) |  |  |  |
| Range (min, max) | (208, 18832) | (208.0, 1557.5) | (213.5, 18832.0) |  |  |  |
| **IgG Ribo P0** |  |  |  | **<0.001** | 0.077 |  |
| Mean (sd) | 708.8 (5006.1) | 876.9 (6081.7) | 359.0 (626.1) |  |  |  |
| Median (Q1,Q3) | 105.2 (58.1, 253.9) | 78.5 (44.5, 203.0) | 203.5 (102.5, 347.0) |  |  |  |
| Range (min, max) | (-18.5, 53438.0) | (-18.5, 53438.0) | (-1.0, 3803.5) |  |  |  |
| **IgG Nucleolin** |  |  |  | **0.02** | 0.272 |  |
| Mean (sd) | 244.3 (192.5) | 226.5 (195.9) | 281.2 (182.1) |  |  |  |
| Median (Q1,Q3) | 189.5 (113.2, 310.8) | 163.5 (108.0, 288.5) | 258.5 (164.0, 329.5) |  |  |  |
| Range (min, max) | (32, 1007) | (32, 1007) | (61.5, 965.0) |  |  |  |
| **IgG SmD3** |  |  |  | **0.009** | 0.216 |  |
| Mean (sd) | 118.6 (266.7) | 97.4 (260.3) | 162.6 (278.0) |  |  |  |
| Median (Q1,Q3) | 34.5 (1.1, 131.0) | 23.5 (-9.0, 99.5) | 75.5 (27.5, 163.0) |  |  |  |
| Range (min, max) | (-86.5, 1607.5) | (-86.5, 1607.5) | (-73.5, 1339.5) |  |  |  |
| **IgM SP D** |  |  |  | 0.37 | 0.716 |  |
| Mean (sd) | 640.1 (610.1) | 611.7 (574.3) | 699.3 (683.3) |  |  |  |
| Median (Q1,Q3) | 463.8 (253.5, 686.9) | 491 (217, 687) | 461.0 (346.0, 665.5) |  |  |  |
| Range (min, max) | (69.5, 3513.5) | (69.5, 3007.0) | (175.5, 3513.5) |  |  |  |
| **IgM Bovin Histone H4 and H2A** |  |  |  | 0.10 | 0.438 |  |
| Mean (sd) | 775.4 (1130.8) | 597.6 (651.5) | 1145.2 (1705.8) |  |  |  |
| Median (Q1,Q3) | 411.5 (192.9, 887.4) | 385.5 (194.0, 751.0) | 605.5 (192.5, 1258.0) |  |  |  |
| Range (min, max) | (35, 8492) | (35.0, 3172.5) | (63.5, 8492.0) |  |  |  |
| **IgM Human core histones** |  |  |  | 0.45 | 0.781 |  |
| Mean (sd) | 320.8 (425.6) | 284.6 (343.6) | 396.2 (557.5) |  |  |  |
| Median (Q1,Q3) | 191.2 (80.6, 389.1) | 145 (81, 377) | 240 (74, 390) |  |  |  |
| Range (min, max) | (17.5, 2722.5) | (17.5, 1934.5) | (32.0, 2722.5) |  |  |  |
| **IgM Human IgG** |  |  |  | 0.11 | 0.44 |  |
| Mean (sd) | 290.7 (535.1) | 250.2 (513.0) | 374.9 (576.6) |  |  |  |
| Median (Q1,Q3) | 121.2 (54.5, 286.6) | 114.5 (53.0, 200.5) | 166.5 (81.5, 439.5) |  |  |  |
| Range (min, max) | (13.5, 3815.0) | (13.5, 3815.0) | (17.5, 3114.5) |  |  |  |
| **IgM HCEC cytop** |  |  |  | 0.73 | 0.889 |  |
| Mean (sd) | 348.9 (207.1) | 320.0 (147.6) | 409.0 (288.4) |  |  |  |
| Median (Q1,Q3) | 296.2 (227.0, 390.1) | 301.0 (226.5, 380.5) | 279.5 (228.5, 492.0) |  |  |  |
| Range (min, max) | (120.5, 1244.5) | (120.5, 1121.0) | (128.0, 1244.5) |  |  |  |
| **IgM MDA5** |  |  |  | 0.88 | 0.959 |  |
| Mean (sd) | 87.5 (105.5) | 87.8 (103.8) | 86.8 (110.5) |  |  |  |
| Median (Q1,Q3) | 49.2 (18.1, 108.6) | 49.5 (18.0, 112.5) | 40.0 (23.5, 106.5) |  |  |  |
| Range (min, max) | (-5.5, 554.5) | (-1.5, 554.5) | (-5.5, 504.5) |  |  |  |
| **IgM Gliadin** |  |  |  | 0.61 | 0.85 |  |
| Mean (sd) | 721.1 (699.4) | 716.3 (660.0) | 731.1 (784.7) |  |  |  |
| Median (Q1,Q3) | 463.5 (232.6, 1064.0) | 519.0 (236.0, 1091.5) | 401 (206, 841) |  |  |  |
| Range (min, max) | (44.0, 3262.5) | (50.5, 3262.5) | (44, 3251) |  |  |  |
| **IgM tTG E coli** |  |  |  | 0.39 | 0.723 |  |
| Mean (sd) | 49.7 (83.0) | 45.1 (75.6) | 59.3 (96.9) |  |  |  |
| Median (Q1,Q3) | 24.2 (11.1, 55.4) | 23.5 (11.5, 51.0) | 25.5 (11.0, 55.5) |  |  |  |
| Range (min, max) | (-5, 571) | (-5, 571) | (-0.5, 546.0) |  |  |  |
| **IgM Myosin Bind Protein C** |  |  |  | 0.79 | 0.911 |  |
| Mean (sd) | 303.0 (347.4) | 306.7 (373.8) | 295.3 (289.5) |  |  |  |
| Median (Q1,Q3) | 176.5 (80.1, 366.1) | 176.5 (79.0, 357.0) | 182.5 (88.5, 399.0) |  |  |  |
| Range (min, max) | (15.5, 1886.0) | (23, 1886) | (15.5, 1191.0) |  |  |  |
| **IgM Tropoelastin** |  |  |  | **0.02** | 0.27 |  |
| Mean (sd) | 1002.6 (783.9) | 1085.1 (748.1) | 830.9 (838.2) |  |  |  |
| Median (Q1,Q3) | 813.0 (402.1, 1313.0) | 895 (477, 1440) | 604.5 (289.5, 1146.0) |  |  |  |
| Range (min, max) | (144.0, 4541.5) | (144, 3519) | (171.0, 4541.5) |  |  |  |
| **IgM Insulin** |  |  |  | 0.35 | 0.701 |  |
| Mean (sd) | 87.9 (258.5) | 79.5 (183.5) | 105.3 (371.7) |  |  |  |
| Median (Q1,Q3) | 18.0 (5.2, 54.5) | 24.5 (6.0, 59.0) | 13.0 (5.0, 51.5) |  |  |  |
| Range (min, max) | (-28.5, 2264.5) | (-28.5, 1199.0) | (-1.0, 2264.5) |  |  |  |
| **IgM Tropomyosin** |  |  |  | 0.51 | 0.813 |  |
| Mean (sd) | 866.1 (1196.8) | 706.3 (664.1) | 1198.6 (1843.1) |  |  |  |
| Median (Q1,Q3) | 496.5 (226.1, 1030.1) | 513.5 (224.0, 926.5) | 432.5 (245.0, 1342.5) |  |  |  |
| Range (min, max) | (52.0, 9482.5) | (52.0, 2990.5) | (85.5, 9482.5) |  |  |  |
| **IgM Myosin M1636** |  |  |  | 0.39 | 0.723 |  |
| Mean (sd) | 24.8 (57.6) | 22.3 (40.7) | 30.1 (82.9) |  |  |  |
| Median (Q1,Q3) | 8.0 (2.0, 22.5) | 9.5 (2.0, 23.5) | 7.5 (1.5, 21.0) |  |  |  |
| Range (min, max) | (-3, 434) | (-3, 288) | (-2.5, 434.0) |  |  |  |
| **IgM Jo 1** |  |  |  | 0.35 | 0.701 |  |
| Mean (sd) | 161.4 (204.3) | 132.4 (142.0) | 221.8 (287.8) |  |  |  |
| Median (Q1,Q3) | 86.2 (34.0, 216.5) | 85.0 (36.5, 196.0) | 91.0 (34.0, 254.5) |  |  |  |
| Range (min, max) | (6, 1254) | (6, 859) | (17, 1254) |  |  |  |
| **IgM Ribo P1** |  |  |  | 0.91 | 0.961 |  |
| Mean (sd) | 256.3 (134.0) | 245.3 (104.6) | 279.1 (180.1) |  |  |  |
| Median (Q1,Q3) | 229.8 (183.6, 295.5) | 230.5 (185.0, 294.0) | 226.0 (177.5, 297.0) |  |  |  |
| Range (min, max) | (94.5, 935.5) | (94.5, 681.0) | (108.5, 935.5) |  |  |  |
| **IgM MPO** |  |  |  | 0.22 | 0.597 |  |
| Mean (sd) | 423.5 (166.0) | 434.1 (158.3) | 401.2 (181.3) |  |  |  |
| Median (Q1,Q3) | 386.0 (307.5, 524.5) | 398.5 (318.5, 531.0) | 359.0 (301.0, 497.5) |  |  |  |
| Range (min, max) | (10.5, 1035.5) | (166.0, 1035.5) | (10.5, 951.5) |  |  |  |
| **IgM Scl 70 Full** |  |  |  | 0.54 | 0.823 |  |
| Mean (sd) | 280.1 (387.4) | 265.3 (411.6) | 311.0 (334.4) |  |  |  |
| Median (Q1,Q3) | 187.0 (104.4, 308.9) | 181.5 (109.0, 266.0) | 191 (81, 421) |  |  |  |
| Range (min, max) | (13.5, 3500.5) | (13.5, 3500.5) | (37.5, 1680.5) |  |  |  |
| **IgM Scl 70 trunc** |  |  |  | 0.77 | 0.904 |  |
| Mean (sd) | 309.7 (282.0) | 292.6 (262.9) | 345.5 (319.0) |  |  |  |
| Median (Q1,Q3) | 234.2 (121.6, 408.9) | 237.5 (122.0, 391.5) | 229.0 (115.5, 499.0) |  |  |  |
| Range (min, max) | (8.5, 1933.0) | (8.5, 1933.0) | (39.5, 1453.0) |  |  |  |
| **IgM SPLUNC2** |  |  |  | 0.30 | 0.669 |  |
| Mean (sd) | 95.1 (89.9) | 103.5 (102.9) | 77.6 (50.2) |  |  |  |
| Median (Q1,Q3) | 75.8 (42.5, 115.2) | 78.5 (44.5, 126.0) | 70.5 (39.0, 100.0) |  |  |  |
| Range (min, max) | (0.5, 722.5) | (12.0, 722.5) | (0.5, 254.5) |  |  |  |
| **IgM Sm Antigens** |  |  |  | 0.71 | 0.879 |  |
| Mean (sd) | 252.0 (239.8) | 259.5 (245.1) | 236.5 (231.1) |  |  |  |
| Median (Q1,Q3) | 175.8 (105.9, 293.4) | 193.5 (107.0, 293.0) | 174.5 (105.5, 318.5) |  |  |  |
| Range (min, max) | (57, 1541) | (58, 1541) | (57.0, 1358.5) |  |  |  |
| IgM Histone H3 1 136 aa |  |  |  | 0.97 | 0.981 |  |
| Mean (sd) | 387.2 (443.6) | 382.9 (459.0) | 396.2 (415.5) |  |  |  |
| Median (Q1,Q3) | 237.5 (144.4, 469.5) | 260.0 (143.5, 470.5) | 197.5 (147.0, 466.5) |  |  |  |
| Range (min, max) | (39.5, 3359.5) | (39.5, 3359.5) | (44.5, 1941.0) |  |  |  |
| **IgM mouse IgM** |  |  |  | 0.97 | 0.983 |  |
| Mean (sd) | 3147.7 (1574.5) | 3190.7 (1635.9) | 3058.0 (1455.8) |  |  |  |
| Median (Q1,Q3) | 3018.0 (1776.1, 3974.9) | 2982.5 (1670.5, 5066.0) | 3069.5 (2067.0, 3354.5) |  |  |  |
| Range (min, max) | (558.5, 6301.5) | (1042, 6119) | (558.5, 6301.5) |  |  |  |
| **IgM Beta galactosidase 2B** |  |  |  | 0.31 | 0.677 |  |
| Mean (sd) | 440.2 (504.2) | 399.1 (429.8) | 525.8 (630.0) |  |  |  |
| Median (Q1,Q3) | 263.2 (134.2, 544.8) | 262.5 (116.0, 479.5) | 264.0 (174.5, 679.5) |  |  |  |
| Range (min, max) | (36.5, 3378.5) | (36.5, 2634.5) | (54.5, 3378.5) |  |  |  |
| **IgM DNA Topoisomerase I Scl 70 non recombinant bovine** |  |  |  | 0.57 | 0.839 |  |
| Mean (sd) | 140.1 (131.8) | 133.1 (131.1) | 154.5 (133.8) |  |  |  |
| Median (Q1,Q3) | 91.8 (53.8, 188.4) | 94.0 (53.5, 171.5) | 82.0 (54.5, 235.0) |  |  |  |
| Range (min, max) | (15.5, 952.0) | (15.5, 952.0) | (26.0, 510.5) |  |  |  |
| **IgM Sm non recombinant bovine** |  |  |  | 0.65 | 0.852 |  |
| Mean (sd) | 549.7 (453.8) | 525.1 (327.8) | 600.7 (644.6) |  |  |  |
| Median (Q1,Q3) | 432.5 (316.8, 644.9) | 429 (331, 646) | 436 (283, 631) |  |  |  |
| Range (min, max) | (120.5, 3647.0) | (120.5, 2433.5) | (145.5, 3647.0) |  |  |  |
| **IgM OGDC E2** |  |  |  | 0.56 | 0.835 |  |
| Mean (sd) | 197.8 (285.9) | 162.8 (198.7) | 270.7 (406.3) |  |  |  |
| Median (Q1,Q3) | 111.2 (40.6, 203.2) | 111.5 (40.5, 190.0) | 106.5 (52.5, 287.0) |  |  |  |
| Range (min, max) | (5.5, 1728.0) | (5.5, 1111.0) | (16, 1728) |  |  |  |
| **IgM mouse fgl2** |  |  |  | 0.99 | 0.996 |  |
| Mean (sd) | 96.4 (95.1) | 92.5 (90.4) | 104.5 (105.1) |  |  |  |
| Median (Q1,Q3) | 66.0 (36.2, 100.4) | 70.0 (37.5, 97.5) | 58.5 (34.0, 121.0) |  |  |  |
| Range (min, max) | (0, 471) | (3.5, 471.0) | (0, 388) |  |  |  |
| **IgM HSP 70** |  |  |  | 0.73 | 0.889 |  |
| Mean (sd) | 259.2 (333.8) | 252.2 (339.6) | 274.0 (325.5) |  |  |  |
| Median (Q1,Q3) | 147.8 (66.2, 329.8) | 142.5 (67.0, 318.5) | 160.0 (64.5, 335.5) |  |  |  |
| Range (min, max) | (3.5, 2389.0) | (11, 2389) | (3.5, 1415.0) |  |  |  |
| **IgM HSP 60** |  |  |  | 0.19 | 0.546 |  |
| Mean (sd) | 276.1 (448.7) | 213.7 (318.0) | 406.0 (626.7) |  |  |  |
| Median (Q1,Q3) | 114.0 (48.5, 250.9) | 110.0 (46.5, 205.5) | 117.0 (57.0, 425.5) |  |  |  |
| Range (min, max) | (-27.5, 2457.0) | (8.5, 1679.0) | (-27.5, 2457.0) |  |  |  |
| **IgM Collagen III C4407** |  |  |  | 0.68 | 0.859 |  |
| Mean (sd) | 91.3 (94.6) | 89.8 (96.3) | 94.5 (92.3) |  |  |  |
| Median (Q1,Q3) | 68.5 (28.8, 119.6) | 68.5 (27.5, 120.0) | 70.5 (33.0, 118.5) |  |  |  |
| Range (min, max) | (-184.0, 488.5) | (-184.0, 487.5) | (13.0, 488.5) |  |  |  |
| **IgM Heparin** |  |  |  | 0.09 | 0.433 |  |
| Mean (sd) | 12.6 (21.8) | 15.1 (22.4) | 7.3 (19.8) |  |  |  |
| Median (Q1,Q3) | 7.8 (2.6, 17.4) | 9.0 (4.5, 17.5) | 4.5 (0.5, 13.0) |  |  |  |
| Range (min, max) | (-66.5, 127.0) | (-15.5, 127.0) | (-66.5, 61.5) |  |  |  |
| **IgM Ebna peptide** |  |  |  | 0.60 | 0.845 |  |
| Mean (sd) | 2.2 (6.6) | 2.4 (7.0) | 1.7 (5.8) |  |  |  |
| Median (Q1,Q3) | 0.5 (-2.4, 4.0) | 0.5 (-2.5, 4.5) | 0.5 (-2.0, 2.5) |  |  |  |
| Range (min, max) | (-8.5, 31.0) | (-8.5, 31.0) | (-5.0, 19.5) |  |  |  |
| **IgM Fib I S** |  |  |  | 0.31 | 0.677 |  |
| Mean (sd) | 264.8 (508.7) | 263.4 (591.4) | 267.8 (271.6) |  |  |  |
| Median (Q1,Q3) | 132.8 (73.0, 258.5) | 125.5 (69.0, 241.5) | 149.5 (88.0, 327.5) |  |  |  |
| Range (min, max) | (18.0, 5088.5) | (25.0, 5088.5) | (18.0, 1010.5) |  |  |  |
| **IgM PL 12** |  |  |  | 0.32 | 0.682 |  |
| Mean (sd) | 680.7 (1298.2) | 675.2 (1456.6) | 692.3 (900.2) |  |  |  |
| Median (Q1,Q3) | 431.2 (202.4, 721.8) | 421 (191, 716) | 480.5 (214.0, 914.5) |  |  |  |
| Range (min, max) | (49.5, 12613.0) | (49.5, 12613.0) | (110, 5471) |  |  |  |
| **IgM La SS B Recombinant human diarect** |  |  |  | 0.10 | 0.44 |  |
| Mean (sd) | 263.6 (328.7) | 231.3 (294.4) | 330.9 (386.3) |  |  |  |
| Median (Q1,Q3) | 152.5 (67.1, 316.6) | 130.5 (62.0, 257.5) | 185.5 (88.0, 401.5) |  |  |  |
| Range (min, max) | (4.5, 1975.5) | (4.5, 1663.5) | (20.5, 1975.5) |  |  |  |
| **IgM Ku p70 p80** |  |  |  | 0.65 | 0.852 |  |
| Mean (sd) | 93.2 (163.5) | 76.7 (95.9) | 127.5 (250.3) |  |  |  |
| Median (Q1,Q3) | 52.0 (22.8, 90.2) | 52.5 (21.0, 85.0) | 51.5 (24.5, 104.0) |  |  |  |
| Range (min, max) | (2.5, 1409.0) | (2.5, 604.0) | (6.5, 1409.0) |  |  |  |
| **IgM empty** |  |  |  | 0.50 | 0.813 |  |
| Mean (sd) | -0.7 (1.8) | -0.8 (1.8) | -0.4 (1.7) |  |  |  |
| Median (Q1,Q3) | -0.5 (-1.5, 0.5) | -1.0 (-2.0, 0.5) | -0.5 (-1.0, 0.5) |  |  |  |
| Range (min, max) | (-6.5, 6.0) | (-6.5, 3.0) | (-4.5, 6.0) |  |  |  |
| **IgM Human H1 Chromatin** |  |  |  | 0.43 | 0.764 |  |
| Mean (sd) | 393.7 (475.2) | 353.7 (373.8) | 476.9 (635.1) |  |  |  |
| Median (Q1,Q3) | 244.0 (125.8, 490.2) | 238.5 (128.0, 462.5) | 268.0 (124.5, 512.0) |  |  |  |
| Range (min, max) | (-9, 3475) | (-9, 2471) | (73, 3475) |  |  |  |
| **IgM Human LGALS3 Galectin 3** |  |  |  | 0.92 | 0.965 |  |
| Mean (sd) | -1.2 (3.7) | -1.0 (2.3) | -1.6 (5.5) |  |  |  |
| Median (Q1,Q3) | -0.5 (-1.5, 0.0) | -0.5 (-1.5, 0.0) | -0.5 (-1.5, 0.5) |  |  |  |
| Range (min, max) | (-33, 3) | (-13, 2) | (-33, 3) |  |  |  |
| **IgM Human IgA** |  |  |  | 0.63 | 0.852 |  |
| Mean (sd) | 212.0 (252.8) | 180.0 (110.2) | 278.5 (410.2) |  |  |  |
| Median (Q1,Q3) | 158.5 (85.8, 236.9) | 158.5 (83.5, 225.5) | 158.5 (112.0, 251.0) |  |  |  |
| Range (min, max) | (23.0, 2225.5) | (49.5, 515.0) | (23.0, 2225.5) |  |  |  |
| **IgM kidney endothelial cell lysate** |  |  |  | 0.89 | 0.959 |  |
| Mean (sd) | 445.6 (187.8) | 445.1 (195.6) | 446.5 (172.9) |  |  |  |
| Median (Q1,Q3) | 403.8 (329.5, 512.8) | 411.5 (337.0, 507.0) | 399.5 (327.0, 517.5) |  |  |  |
| Range (min, max) | (218.5, 1374.5) | (218.5, 1374.5) | (248.5, 1012.0) |  |  |  |
| **IgM MYH6** |  |  |  | 0.27 | 0.645 |  |
| Mean (sd) | 495.5 (617.9) | 512.4 (708.1) | 460.2 (371.5) |  |  |  |
| Median (Q1,Q3) | 305.2 (166.4, 576.4) | 281.5 (146.0, 549.0) | 341.0 (212.5, 589.0) |  |  |  |
| Range (min, max) | (42.5, 4423.5) | (42.5, 4423.5) | (60.0, 1750.5) |  |  |  |
| **IgM Ro SS A 60kD recombinant** |  |  |  | 0.34 | 0.689 |  |
| Mean (sd) | 119.8 (225.1) | 100.1 (139.2) | 160.7 (340.1) |  |  |  |
| Median (Q1,Q3) | 54.8 (21.1, 107.5) | 55 (19, 113) | 54.0 (26.5, 106.0) |  |  |  |
| Range (min, max) | (4.5, 1950.5) | (4.5, 889.5) | (8.5, 1950.5) |  |  |  |
| **IgM Sp100** |  |  |  | 0.71 | 0.879 |  |
| Mean (sd) | 245.2 (285.2) | 254.6 (305.9) | 225.7 (239.1) |  |  |  |
| Median (Q1,Q3) | 153.8 (60.1, 346.5) | 164.0 (55.5, 351.5) | 143.0 (77.5, 243.5) |  |  |  |
| Range (min, max) | (-20.5, 2068.0) | (13.5, 2068.0) | (-20.5, 927.0) |  |  |  |
| **IgM B2AR** |  |  |  | 0.08 | 0.408 |  |
| Mean (sd) | 123.9 (143.1) | 131.1 (126.9) | 109.1 (173.0) |  |  |  |
| Median (Q1,Q3) | 67.8 (24.5, 190.9) | 81.5 (31.0, 198.5) | 38.5 (22.0, 128.5) |  |  |  |
| Range (min, max) | (-3.0, 894.5) | (-1.0, 539.5) | (-3.0, 894.5) |  |  |  |
| **IgM AT1** |  |  |  | 0.11 | 0.44 |  |
| Mean (sd) | 34.6 (48.2) | 32.4 (46.0) | 39.0 (52.9) |  |  |  |
| Median (Q1,Q3) | 21.8 (10.8, 36.8) | 19.0 (8.5, 34.0) | 28.5 (16.0, 37.0) |  |  |  |
| Range (min, max) | (-5, 292) | (-5.0, 279.5) | (1.5, 292.0) |  |  |  |
| **IgM ICAM 1** |  |  |  | 0.54 | 0.823 |  |
| Mean (sd) | 15.5 (38.2) | 17.2 (35.2) | 12.0 (44.1) |  |  |  |
| Median (Q1,Q3) | 8.2 (1.5, 16.4) | 9.5 (1.5, 22.0) | 8.0 (1.5, 15.5) |  |  |  |
| Range (min, max) | (-114.5, 240.5) | (-54.0, 240.5) | (-114.5, 234.5) |  |  |  |
| **IgM HSP 27** |  |  |  | 0.86 | 0.947 |  |
| Mean (sd) | 396.6 (607.8) | 377.0 (602.3) | 437.4 (625.3) |  |  |  |
| Median (Q1,Q3) | 211.8 (93.5, 428.5) | 218.5 (103.5, 432.0) | 192.0 (85.5, 409.0) |  |  |  |
| Range (min, max) | (16.5, 4128.0) | (16.5, 4128.0) | (42.5, 2362.0) |  |  |  |
| **IgM Actin Bovine** |  |  |  | 0.95 | 0.976 |  |
| Mean (sd) | 37.3 (58.9) | 35.9 (51.8) | 40.4 (72.2) |  |  |  |
| Median (Q1,Q3) | 17.8 (9.0, 40.2) | 18.0 (9.5, 44.5) | 16.5 (9.0, 31.5) |  |  |  |
| Range (min, max) | (0, 413) | (0.0, 286.5) | (4.5, 413.0) |  |  |  |
| **IgM Actin Rabbit** |  |  |  | 0.77 | 0.903 |  |
| Mean (sd) | 47.8 (104.3) | 42.1 (70.9) | 59.7 (152.7) |  |  |  |
| Median (Q1,Q3) | 16.8 (6.6, 49.6) | 16 (6, 54) | 17.5 (7.0, 40.5) |  |  |  |
| Range (min, max) | (-1, 898) | (-1.0, 459.5) | (0.5, 898.0) |  |  |  |
| **IgM Aggrecan recombinant** |  |  |  | 0.41 | 0.752 |  |
| Mean (sd) | 13.7 (32.5) | 15.2 (36.0) | 10.7 (23.8) |  |  |  |
| Median (Q1,Q3) | 4.5 (-0.5, 12.8) | 5.5 (-0.5, 16.5) | 3.0 (-0.5, 9.0) |  |  |  |
| Range (min, max) | (-4.5, 254.5) | (-4.5, 254.5) | (-4, 103) |  |  |  |
| **IgM PBS** |  |  |  | **0.01** | 0.224 |  |
| Mean (sd) | 15.4 (38.1) | 16.0 (29.3) | 14.2 (52.4) |  |  |  |
| Median (Q1,Q3) | 3.0 (0.0, 16.4) | 6.0 (0.5, 20.5) | 1.0 (-2.0, 6.5) |  |  |  |
| Range (min, max) | (-5, 313) | (-5.0, 193.5) | (-4, 313) |  |  |  |
| **IgM Thyrogobulin** |  |  |  | 0.67 | 0.854 |  |
| Mean (sd) | 87.8 (104.1) | 88.2 (98.6) | 86.8 (116.1) |  |  |  |
| Median (Q1,Q3) | 59.0 (35.0, 91.8) | 60.0 (34.0, 98.5) | 55.5 (39.0, 74.5) |  |  |  |
| Range (min, max) | (16.0, 693.5) | (16.0, 560.5) | (19.5, 693.5) |  |  |  |
| **IgM BPI** |  |  |  | **0.03** | 0.277 |  |
| Mean (sd) | 101.2 (204.9) | 77.5 (106.7) | 150.6 (322.5) |  |  |  |
| Median (Q1,Q3) | 60.0 (35.6, 100.1) | 53.0 (30.5, 85.0) | 83.0 (38.5, 113.5) |  |  |  |
| Range (min, max) | (5.5, 1975.0) | (5.5, 896.5) | (22, 1975) |  |  |  |
| **IgM SCGB1A1** |  |  |  | 0.56 | 0.835 |  |
| Mean (sd) | 450.6 (1062.7) | 350.0 (488.7) | 660.0 (1724.6) |  |  |  |
| Median (Q1,Q3) | 163.2 (94.1, 430.1) | 154.5 (99.0, 366.5) | 264.5 (92.0, 653.5) |  |  |  |
| Range (min, max) | (-169.5, 10539.5) | (23.5, 2842.5) | (-169.5, 10539.5) |  |  |  |
| **IgM Bovin Histone subclass F1** |  |  |  | 0.22 | 0.581 |  |
| Mean (sd) | 975.1 (1182.2) | 897.9 (1141.5) | 1135.8 (1263.6) |  |  |  |
| Median (Q1,Q3) | 553.0 (257.4, 1064.6) | 488.5 (247.0, 978.5) | 734.0 (271.0, 1110.5) |  |  |  |
| Range (min, max) | (47.5, 6171.0) | (47.5, 6171.0) | (73.5, 5493.5) |  |  |  |
| **IgM Histone H2B** |  |  |  | 0.33 | 0.689 |  |
| Mean (sd) | 1124.5 (1301.1) | 1041.9 (1253.9) | 1296.5 (1396.3) |  |  |  |
| Median (Q1,Q3) | 674.2 (320.0, 1370.6) | 644.5 (299.5, 1292.0) | 749.5 (358.5, 1459.5) |  |  |  |
| Range (min, max) | (63.5, 7410.0) | (63.5, 7410.0) | (162, 5682) |  |  |  |
| **IgM human IgG F ab 2** |  |  |  | 0.08 | 0.396 |  |
| Mean (sd) | 199.0 (390.5) | 202.6 (318.4) | 191.5 (514.3) |  |  |  |
| Median (Q1,Q3) | 96.8 (57.1, 160.2) | 102.0 (60.5, 203.5) | 82.5 (42.0, 148.5) |  |  |  |
| Range (min, max) | (3.0, 3174.5) | (10.0, 1939.5) | (3.0, 3174.5) |  |  |  |
| **IgM HCEC memb** |  |  |  | **0.03** | 0.277 |  |
| Mean (sd) | 223.3 (170.6) | 192.3 (130.8) | 287.8 (221.2) |  |  |  |
| Median (Q1,Q3) | 169.2 (118.0, 247.8) | 160.5 (110.5, 227.0) | 198.0 (133.5, 425.5) |  |  |  |
| Range (min, max) | (15.5, 947.5) | (15.5, 693.5) | (93.0, 947.5) |  |  |  |
| **IgM Glycyl tRNA Synthetase EJ** |  |  |  | 0.57 | 0.839 |  |
| Mean (sd) | 107.2 (141.5) | 109.6 (155.1) | 102.1 (109.7) |  |  |  |
| Median (Q1,Q3) | 62.2 (21.6, 144.2) | 60 (18, 148) | 66.5 (38.5, 137.0) |  |  |  |
| Range (min, max) | (-4.0, 1003.5) | (-4.0, 1003.5) | (4, 516) |  |  |  |
| **IgM Nup62** |  |  |  | 0.41 | 0.752 |  |
| Mean (sd) | 148.8 (277.0) | 145.6 (286.1) | 155.5 (260.8) |  |  |  |
| Median (Q1,Q3) | 63.2 (28.9, 150.8) | 56.0 (27.5, 158.5) | 77.5 (36.5, 138.0) |  |  |  |
| Range (min, max) | (7.5, 1788.0) | (7.5, 1788.0) | (8.5, 1472.5) |  |  |  |
| **IgM LCI** |  |  |  | 0.49 | 0.802 |  |
| Mean (sd) | 178.2 (313.5) | 155.0 (197.8) | 226.5 (471.5) |  |  |  |
| Median (Q1,Q3) | 74.8 (37.0, 205.8) | 75 (32, 206) | 73 (45, 188) |  |  |  |
| Range (min, max) | (4.5, 2761.0) | (4.5, 1095.5) | (11.5, 2761.0) |  |  |  |
| **IgM alphaB crystallin** |  |  |  | 0.80 | 0.919 |  |
| Mean (sd) | 953.3 (1471.5) | 927.6 (1508.0) | 1006.7 (1411.1) |  |  |  |
| Median (Q1,Q3) | 439.5 (198.1, 1041.5) | 472.0 (239.0, 1029.5) | 418 (174, 1083) |  |  |  |
| Range (min, max) | (33.0, 10343.5) | (33.0, 10343.5) | (50.5, 6321.5) |  |  |  |
| **IgM Beta 2 Glyc recombinant human** |  |  |  | 0.60 | 0.845 |  |
| Mean (sd) | 194.3 (219.4) | 202.2 (238.2) | 177.8 (175.7) |  |  |  |
| Median (Q1,Q3) | 120.0 (69.8, 232.8) | 124.5 (78.5, 236.0) | 103.0 (66.5, 220.0) |  |  |  |
| Range (min, max) | (28.5, 1334.5) | (37.0, 1334.5) | (28.5, 800.0) |  |  |  |
| **IgM Ox LDL** |  |  |  | 0.17 | 0.513 |  |
| Mean (sd) | 155.7 (268.1) | 129.0 (193.5) | 211.4 (376.6) |  |  |  |
| Median (Q1,Q3) | 61.2 (27.1, 145.0) | 59.5 (25.5, 130.5) | 74.5 (33.5, 180.0) |  |  |  |
| Range (min, max) | (-3, 1706) | (-3, 1065) | (11.5, 1706.0) |  |  |  |
| **IgM Proteoglycan** |  |  |  | 0.76 | 0.903 |  |
| Mean (sd) | 34.4 (47.8) | 35.0 (45.4) | 33.1 (53.2) |  |  |  |
| Median (Q1,Q3) | 18.8 (8.1, 41.2) | 19.0 (8.0, 42.5) | 16 (9, 33) |  |  |  |
| Range (min, max) | (-12.5, 297.5) | (-12.5, 239.0) | (-2.5, 297.5) |  |  |  |
| **IgM LG3** |  |  |  | 0.16 | 0.49 |  |
| Mean (sd) | 302.2 (337.8) | 303.2 (260.5) | 300.2 (463.6) |  |  |  |
| Median (Q1,Q3) | 213.2 (86.1, 418.4) | 233.0 (102.5, 418.5) | 116.5 (63.0, 367.0) |  |  |  |
| Range (min, max) | (8.5, 2645.5) | (8.5, 1221.5) | (14.0, 2645.5) |  |  |  |
| **IgM Ribo P2** |  |  |  | 0.66 | 0.852 |  |
| Mean (sd) | 27.0 (36.6) | 29.1 (40.9) | 22.6 (25.1) |  |  |  |
| Median (Q1,Q3) | 15.0 (8.0, 33.9) | 15.0 (7.0, 39.5) | 14.5 (8.0, 28.5) |  |  |  |
| Range (min, max) | (-3, 292) | (-1.5, 292.0) | (-3, 126) |  |  |  |
| **IgM SmD2** |  |  |  | 0.51 | 0.813 |  |
| Mean (sd) | 52.9 (55.9) | 57.8 (62.2) | 42.7 (38.4) |  |  |  |
| Median (Q1,Q3) | 31.8 (16.5, 67.4) | 33 (16, 74) | 30 (18, 45) |  |  |  |
| Range (min, max) | (2.5, 321.5) | (2.5, 321.5) | (3.0, 175.5) |  |  |  |
| **IgM PR3** |  |  |  | 0.45 | 0.781 |  |
| Mean (sd) | 34.2 (35.2) | 36.1 (38.4) | 30.0 (27.4) |  |  |  |
| Median (Q1,Q3) | 25.2 (13.8, 42.9) | 25.5 (14.5, 43.0) | 17.5 (13.5, 41.0) |  |  |  |
| Range (min, max) | (1.0, 197.5) | (3.5, 197.5) | (1, 136) |  |  |  |
| **IgM snRNP C** |  |  |  | 0.79 | 0.911 |  |
| Mean (sd) | 376.3 (219.5) | 361.4 (176.7) | 407.4 (289.5) |  |  |  |
| Median (Q1,Q3) | 350.5 (223.0, 436.8) | 355.5 (240.0, 430.0) | 300.5 (204.0, 497.0) |  |  |  |
| Range (min, max) | (119.5, 1539.0) | (119.5, 1307.0) | (134, 1539) |  |  |  |
| **IgM snRNP 68 B B** |  |  |  | 0.75 | 0.894 |  |
| Mean (sd) | 1218.4 (600.8) | 1162.5 (364.2) | 1334.7 (912.4) |  |  |  |
| Median (Q1,Q3) | 1158.2 (802.2, 1428.8) | 1187.0 (827.5, 1387.5) | 1026.5 (753.5, 1630.5) |  |  |  |
| Range (min, max) | (482.0, 5218.5) | (482, 2198) | (521.5, 5218.5) |  |  |  |
| **IgM Mucarinic Rceptor 3** |  |  |  | 0.28 | 0.659 |  |
| Mean (sd) | 178.0 (307.5) | 137.6 (140.3) | 262.1 (494.5) |  |  |  |
| Median (Q1,Q3) | 94.8 (30.9, 221.9) | 88.0 (26.5, 225.0) | 100.0 (56.5, 212.5) |  |  |  |
| Range (min, max) | (6.0, 2758.5) | (6.0, 757.5) | (14.5, 2758.5) |  |  |  |
| **IgM Bovine Histone H3** |  |  |  | 0.10 | 0.44 |  |
| Mean (sd) | 2565.9 (2738.3) | 2253.6 (2411.0) | 3215.8 (3259.3) |  |  |  |
| Median (Q1,Q3) | 1528.8 (782.6, 3566.4) | 1202.0 (695.0, 3264.5) | 1969.5 (1014.0, 4836.5) |  |  |  |
| Range (min, max) | (52.5, 14698.5) | (52.5, 13079.0) | (191.5, 14698.5) |  |  |  |
| **IgM Histone H2A H2B dimers** |  |  |  | 0.49 | 0.8 |  |
| Mean (sd) | 809.5 (972.9) | 779.8 (980.8) | 871.4 (966.5) |  |  |  |
| Median (Q1,Q3) | 442.8 (216.9, 969.0) | 436.0 (213.5, 959.0) | 449.5 (247.5, 1206.5) |  |  |  |
| Range (min, max) | (48.5, 5809.0) | (48.5, 5809.0) | (94.5, 4292.0) |  |  |  |
| **IgM mouse IgG F ab 2** |  |  |  | 0.38 | 0.717 |  |
| Mean (sd) | 87.5 (198.4) | 86.6 (217.9) | 89.1 (152.9) |  |  |  |
| Median (Q1,Q3) | 36.5 (13.5, 95.6) | 33.0 (13.5, 90.0) | 44.0 (14.0, 103.5) |  |  |  |
| Range (min, max) | (-2.5, 1812.5) | (5.0, 1812.5) | (-2.5, 908.5) |  |  |  |
| **IgM human intestinal smooth muscle cell** |  |  |  | 0.38 | 0.717 |  |
| Mean (sd) | 357.9 (164.8) | 338.4 (122.6) | 398.5 (225.8) |  |  |  |
| Median (Q1,Q3) | 319.8 (242.5, 407.5) | 319.0 (240.5, 398.5) | 340.5 (259.0, 426.0) |  |  |  |
| Range (min, max) | (140.5, 1202.0) | (140.5, 700.5) | (162.5, 1202.0) |  |  |  |
| **IgM Intrinsic Factor** |  |  |  | 0.45 | 0.781 |  |
| Mean (sd) | 161.5 (211.2) | 163.0 (232.7) | 158.4 (159.9) |  |  |  |
| Median (Q1,Q3) | 97.0 (43.5, 201.8) | 96.5 (41.0, 199.5) | 102.0 (53.0, 203.5) |  |  |  |
| Range (min, max) | (8.5, 1689.5) | (8.5, 1689.5) | (26.5, 679.0) |  |  |  |
| **IgM RNP Sm non recombinant bovine** |  |  |  | 0.90 | 0.959 |  |
| Mean (sd) | 329.6 (289.9) | 302.4 (227.0) | 386.3 (387.4) |  |  |  |
| Median (Q1,Q3) | 228.2 (173.6, 352.0) | 230.5 (185.0, 339.5) | 221.0 (162.5, 399.0) |  |  |  |
| Range (min, max) | (90, 1680) | (90.0, 1534.5) | (102.5, 1680.0) |  |  |  |
| **IgM PDC E2** |  |  |  | 0.11 | 0.44 |  |
| Mean (sd) | 201.9 (289.4) | 169.7 (235.3) | 268.8 (373.2) |  |  |  |
| Median (Q1,Q3) | 99.8 (47.5, 208.9) | 99 (31, 191) | 124.5 (55.5, 330.5) |  |  |  |
| Range (min, max) | (3.0, 1785.5) | (3, 1172) | (18.0, 1785.5) |  |  |  |
| **IgM dsDNA plasmid** |  |  |  | 0.60 | 0.845 |  |
| Mean (sd) | 612.0 (729.3) | 571.8 (628.5) | 695.7 (908.2) |  |  |  |
| Median (Q1,Q3) | 376.0 (218.2, 721.5) | 371.5 (216.5, 750.0) | 473.5 (261.0, 640.0) |  |  |  |
| Range (min, max) | (35, 5071) | (45, 4018) | (35, 5071) |  |  |  |
| **IgM Measles** |  |  |  | 0.11 | 0.44 |  |
| Mean (sd) | 169.9 (220.4) | 133.5 (134.6) | 245.8 (324.8) |  |  |  |
| Median (Q1,Q3) | 93.8 (41.8, 204.1) | 86.5 (34.0, 193.5) | 139.0 (43.0, 256.5) |  |  |  |
| Range (min, max) | (6.5, 1360.0) | (6.5, 706.5) | (21, 1360) |  |  |  |
| **IgM Laminin** |  |  |  | 0.89 | 0.959 |  |
| Mean (sd) | 17.8 (19.8) | 18.9 (21.1) | 15.6 (16.9) |  |  |  |
| Median (Q1,Q3) | 12.2 (5.5, 22.5) | 12.0 (5.5, 23.0) | 12.5 (6.5, 22.5) |  |  |  |
| Range (min, max) | (-21.5, 100.0) | (-10.5, 100.0) | (-21.5, 82.5) |  |  |  |
| **IgM Collagen V C3657** |  |  |  | 0.93 | 0.965 |  |
| Mean (sd) | 137.3 (165.6) | 119.1 (99.9) | 175.1 (250.6) |  |  |  |
| Median (Q1,Q3) | 85.8 (50.9, 160.2) | 87.5 (50.0, 163.5) | 83.5 (53.0, 154.0) |  |  |  |
| Range (min, max) | (-18.5, 1164.0) | (-18.5, 531.5) | (-3.5, 1164.0) |  |  |  |
| **IgM Porcine Myosin Heart** |  |  |  | 0.06 | 0.366 |  |
| Mean (sd) | 386.2 (982.6) | 382.1 (1151.2) | 394.9 (482.1) |  |  |  |
| Median (Q1,Q3) | 187.2 (85.0, 377.2) | 177.5 (88.5, 273.0) | 275.0 (76.5, 463.5) |  |  |  |
| Range (min, max) | (23, 9158) | (23, 9158) | (41, 2351) |  |  |  |
| **IgM TUBA1B protein** |  |  |  | 0.58 | 0.84 |  |
| Mean (sd) | 4.1 (10.2) | 4.5 (11.5) | 3.2 (6.7) |  |  |  |
| Median (Q1,Q3) | 1.0 (-1.5, 7.1) | 0.0 (-2.0, 7.5) | 1.5 (-1.0, 3.5) |  |  |  |
| Range (min, max) | (-7.0, 65.5) | (-7.0, 65.5) | (-5.5, 22.0) |  |  |  |
| **IgM Aldolase Type X** |  |  |  | 1.00 | 1 |  |
| Mean (sd) | 2.4 (13.4) | 3.4 (15.5) | 0.5 (7.1) |  |  |  |
| Median (Q1,Q3) | -1.8 (-4.5, 2.9) | -1.5 (-4.5, 4.0) | -2.0 (-4.5, 2.5) |  |  |  |
| Range (min, max) | (-8.5, 75.0) | (-8.5, 75.0) | (-8, 23) |  |  |  |
| **IgM TPO** |  |  |  | 0.43 | 0.759 |  |
| Mean (sd) | 317.3 (438.1) | 306.2 (419.5) | 340.4 (479.7) |  |  |  |
| Median (Q1,Q3) | 162.8 (84.0, 328.6) | 157 (78, 309) | 178.0 (94.0, 341.5) |  |  |  |
| Range (min, max) | (20.5, 2731.5) | (20.5, 2245.0) | (52.0, 2731.5) |  |  |  |
| **IgM SRP54** |  |  |  | 0.44 | 0.775 |  |
| Mean (sd) | 506.4 (485.6) | 473.2 (420.9) | 575.4 (599.2) |  |  |  |
| Median (Q1,Q3) | 347.2 (234.9, 586.9) | 376 (199, 561) | 325.5 (264.5, 626.5) |  |  |  |
| Range (min, max) | (74.0, 3331.5) | (74, 2807) | (81.0, 3331.5) |  |  |  |
| **IgM PL 7** |  |  |  | 0.72 | 0.879 |  |
| Mean (sd) | 242.1 (244.1) | 232.7 (205.6) | 261.7 (311.9) |  |  |  |
| Median (Q1,Q3) | 168.2 (74.0, 317.4) | 181.5 (80.0, 319.0) | 154.0 (63.0, 250.5) |  |  |  |
| Range (min, max) | (16.0, 1430.5) | (16.0, 1051.5) | (20.0, 1430.5) |  |  |  |
| **IgM human albumin** |  |  |  | 0.95 | 0.976 |  |
| Mean (sd) | 118.7 (183.0) | 121.0 (199.5) | 113.8 (145.3) |  |  |  |
| Median (Q1,Q3) | 60.8 (31.8, 124.6) | 57.5 (34.0, 116.5) | 72 (30, 127) |  |  |  |
| Range (min, max) | (-17.5, 1402.0) | (5, 1402) | (-17.5, 695.0) |  |  |  |
| **IgM C1q purified non recombinant** |  |  |  | 0.70 | 0.877 |  |
| Mean (sd) | 242.1 (255.5) | 221.3 (165.3) | 285.5 (380.0) |  |  |  |
| Median (Q1,Q3) | 183.2 (110.1, 263.5) | 175.0 (106.5, 261.5) | 183.5 (123.5, 269.0) |  |  |  |
| Range (min, max) | (5.5, 1868.5) | (5.5, 901.0) | (47.0, 1868.5) |  |  |  |
| **IgM Human LEDGF** |  |  |  | 0.13 | 0.449 |  |
| Mean (sd) | 646.3 (813.7) | 577.8 (810.9) | 788.7 (811.9) |  |  |  |
| Median (Q1,Q3) | 397.2 (245.5, 705.2) | 393.0 (216.5, 604.5) | 467.5 (261.0, 1017.5) |  |  |  |
| Range (min, max) | (82.5, 5194.0) | (82.5, 5194.0) | (115.5, 4148.0) |  |  |  |
| **IgM mouse IgG Fc** |  |  |  | 0.34 | 0.689 |  |
| Mean (sd) | 116.4 (106.4) | 119.8 (101.2) | 109.2 (117.5) |  |  |  |
| Median (Q1,Q3) | 83.2 (44.6, 152.5) | 95 (46, 161) | 74.5 (42.0, 127.5) |  |  |  |
| Range (min, max) | (11.5, 632.5) | (11.5, 474.5) | (14.0, 632.5) |  |  |  |
| **IgM Troponin I** |  |  |  | 0.68 | 0.859 |  |
| Mean (sd) | 254.3 (242.5) | 246.0 (222.5) | 271.8 (282.0) |  |  |  |
| Median (Q1,Q3) | 178.8 (90.5, 340.1) | 178.5 (90.5, 337.5) | 182.0 (90.5, 341.0) |  |  |  |
| Range (min, max) | (37, 1287) | (37, 945) | (55, 1287) |  |  |  |
| **IgM beta 2 GPI non recombinant Bovine** |  |  |  | 0.48 | 0.794 |  |
| Mean (sd) | 66.2 (48.0) | 65.5 (38.7) | 67.6 (63.7) |  |  |  |
| Median (Q1,Q3) | 55.5 (35.1, 85.8) | 58.0 (40.0, 86.5) | 51.5 (29.0, 79.5) |  |  |  |
| Range (min, max) | (-50.5, 294.5) | (2.5, 176.5) | (-50.5, 294.5) |  |  |  |
| **IgM Ro SS A 60 kD non recombinant bovine** |  |  |  | 0.46 | 0.781 |  |
| Mean (sd) | 66.3 (95.1) | 61.5 (76.2) | 76.3 (126.4) |  |  |  |
| Median (Q1,Q3) | 32.8 (16.2, 78.5) | 33.0 (15.0, 78.5) | 32.5 (20.0, 78.5) |  |  |  |
| Range (min, max) | (-2.5, 706.5) | (-2.5, 445.0) | (8.5, 706.5) |  |  |  |
| **IgM M2** |  |  |  | 0.33 | 0.689 |  |
| Mean (sd) | 283.7 (326.4) | 248.5 (285.1) | 357.0 (393.1) |  |  |  |
| Median (Q1,Q3) | 175.0 (87.1, 305.5) | 175 (84, 271) | 175 (95, 501) |  |  |  |
| Range (min, max) | (12, 1494) | (12, 1494) | (26, 1427) |  |  |  |
| **IgM M2AR** |  |  |  | 0.81 | 0.919 |  |
| Mean (sd) | 11.5 (40.2) | 8.5 (9.5) | 17.8 (69.4) |  |  |  |
| Median (Q1,Q3) | 5.5 (2.5, 11.5) | 5.5 (2.5, 12.5) | 5.5 (3.0, 11.0) |  |  |  |
| Range (min, max) | (-2.0, 427.5) | (-2.0, 49.5) | (-0.5, 427.5) |  |  |  |
| **IgM A1AR** |  |  |  | 0.07 | 0.389 |  |
| Mean (sd) | 21.2 (44.0) | 26.3 (51.5) | 10.8 (18.2) |  |  |  |
| Median (Q1,Q3) | 7.8 (1.0, 23.4) | 9.0 (1.5, 26.0) | 6.0 (-0.5, 12.0) |  |  |  |
| Range (min, max) | (-4.0, 286.5) | (-3.0, 286.5) | (-4, 91) |  |  |  |
| **IgM Troponin C** |  |  |  | 0.13 | 0.449 |  |
| Mean (sd) | 208.6 (290.0) | 225.1 (292.6) | 174.2 (285.2) |  |  |  |
| Median (Q1,Q3) | 130.5 (51.8, 235.4) | 137 (58, 258) | 111 (44, 177) |  |  |  |
| Range (min, max) | (-159.0, 2027.5) | (9.5, 2027.5) | (-159.0, 1573.5) |  |  |  |
| **IgM HSP 47** |  |  |  | 0.13 | 0.449 |  |
| Mean (sd) | 127.4 (120.9) | 130.5 (108.2) | 120.8 (145.4) |  |  |  |
| Median (Q1,Q3) | 107.5 (38.0, 148.9) | 114.0 (49.5, 179.0) | 77.0 (26.5, 145.5) |  |  |  |
| Range (min, max) | (9.0, 683.5) | (10, 655) | (9.0, 683.5) |  |  |  |
| **IgM alpha Actinin** |  |  |  | 0.26 | 0.642 |  |
| Mean (sd) | 10.1 (16.1) | 8.2 (8.8) | 14.1 (25.1) |  |  |  |
| Median (Q1,Q3) | 5.8 (3.0, 10.9) | 5.5 (2.5, 11.0) | 6.5 (3.5, 10.5) |  |  |  |
| Range (min, max) | (-2.5, 137.0) | (-2.5, 40.5) | (1.5, 137.0) |  |  |  |
| **IgM Alpha KGDH** |  |  |  | 0.41 | 0.746 |  |
| Mean (sd) | 34.5 (32.8) | 33.9 (32.8) | 35.7 (33.0) |  |  |  |
| Median (Q1,Q3) | 24.2 (12.5, 49.8) | 24.0 (11.5, 50.5) | 26.5 (16.5, 47.5) |  |  |  |
| Range (min, max) | (-38.0, 164.5) | (-5.5, 164.5) | (-38, 119) |  |  |  |
| **IgM Fib IV** |  |  |  | 0.48 | 0.795 |  |
| Mean (sd) | 40.3 (61.4) | 44.4 (70.8) | 32.0 (33.8) |  |  |  |
| Median (Q1,Q3) | 24.0 (8.1, 51.4) | 25.5 (8.5, 51.5) | 21.5 (7.0, 49.0) |  |  |  |
| Range (min, max) | (-3, 511) | (-1, 511) | (-3, 133) |  |  |  |
| **IgM PCNA** |  |  |  | 0.80 | 0.919 |  |
| Mean (sd) | 186.2 (537.2) | 155.6 (286.1) | 249.8 (852.6) |  |  |  |
| Median (Q1,Q3) | 74.0 (27.8, 145.8) | 74.0 (26.0, 142.5) | 74.0 (28.5, 147.5) |  |  |  |
| Range (min, max) | (-20, 5230) | (-2, 1864) | (-20, 5230) |  |  |  |
| **IgM snRNP A** |  |  |  | 0.93 | 0.965 |  |
| Mean (sd) | 540.8 (484.1) | 498.4 (361.6) | 629.0 (669.2) |  |  |  |
| Median (Q1,Q3) | 398.8 (217.2, 649.4) | 415.5 (244.0, 642.5) | 328.0 (210.0, 736.5) |  |  |  |
| Range (min, max) | (105.0, 2682.5) | (106, 1953) | (105.0, 2682.5) |  |  |  |
| **IgM PM Scl 75** |  |  |  | **0.05** | 0.337 |  |
| Mean (sd) | 318.3 (470.7) | 261.4 (418.7) | 436.7 (551.3) |  |  |  |
| Median (Q1,Q3) | 152.8 (68.6, 325.2) | 138.0 (61.5, 274.5) | 210.5 (87.5, 589.0) |  |  |  |
| Range (min, max) | (-2.5, 2683.0) | (-2.5, 2683.0) | (13, 2145) |  |  |  |
| **IgM human C1q Abcam** |  |  |  | 0.16 | 0.492 |  |
| Mean (sd) | 8443.6 (1974.5) | 8250.9 (1874.3) | 8844.7 (2139.2) |  |  |  |
| Median (Q1,Q3) | 8299.2 (7215.4, 9404.1) | 8200.0 (7219.5, 9212.0) | 8863.0 (7091.5, 9594.5) |  |  |  |
| Range (min, max) | (4797.5, 15362.0) | (4797.5, 14682.0) | (5197, 15362) |  |  |  |
| **IgM whole histones** |  |  |  | **0.03** | 0.282 |  |
| Mean (sd) | 907.2 (1279.6) | 708.0 (803.4) | 1321.5 (1875.0) |  |  |  |
| Median (Q1,Q3) | 478.8 (243.5, 1078.4) | 395.0 (240.5, 954.5) | 881.5 (304.0, 1323.5) |  |  |  |
| Range (min, max) | (29.0, 10179.5) | (29, 4743) | (120.5, 10179.5) |  |  |  |
| **IgM human nucleosome** |  |  |  | 0.17 | 0.511 |  |
| Mean (sd) | 129.8 (177.3) | 104.6 (108.3) | 182.3 (264.0) |  |  |  |
| Median (Q1,Q3) | 67.0 (34.5, 158.5) | 63.5 (34.5, 132.0) | 105 (41, 165) |  |  |  |
| Range (min, max) | (9.5, 1265.0) | (9.5, 640.5) | (23.5, 1265.0) |  |  |  |
| **IgM human IgG Fc** |  |  |  | 0.37 | 0.716 |  |
| Mean (sd) | 238.1 (424.8) | 238.3 (481.4) | 237.6 (277.6) |  |  |  |
| Median (Q1,Q3) | 120.2 (47.1, 243.0) | 110.5 (49.0, 219.0) | 141.0 (41.0, 275.5) |  |  |  |
| Range (min, max) | (-15.0, 3669.5) | (11.0, 3669.5) | (-15.0, 1033.5) |  |  |  |
| **IgM HCEC total Triton** |  |  |  | 0.31 | 0.677 |  |
| Mean (sd) | 1225.5 (571.5) | 1235.5 (505.8) | 1204.7 (695.9) |  |  |  |
| Median (Q1,Q3) | 1160.8 (756.2, 1572.6) | 1179.5 (835.0, 1595.5) | 1103.5 (703.0, 1456.5) |  |  |  |
| Range (min, max) | (323.5, 3347.0) | (323.5, 2890.0) | (434, 3347) |  |  |  |
| **IgM TIF1 gamma** |  |  |  | 0.26 | 0.645 |  |
| Mean (sd) | 338.4 (374.7) | 287.6 (234.5) | 444.2 (554.6) |  |  |  |
| Median (Q1,Q3) | 249.0 (164.8, 351.1) | 235.5 (164.5, 347.5) | 275.5 (176.0, 355.0) |  |  |  |
| Range (min, max) | (48.5, 2739.5) | (48.5, 1854.5) | (92.5, 2739.5) |  |  |  |
| **IgM gp210** |  |  |  | 0.58 | 0.84 |  |
| Mean (sd) | 1583.7 (1281.4) | 1527.4 (1184.0) | 1700.7 (1474.4) |  |  |  |
| Median (Q1,Q3) | 1210.5 (817.5, 1787.9) | 1225.0 (887.0, 1644.5) | 1020.5 (766.0, 1817.0) |  |  |  |
| Range (min, max) | (273.0, 7010.5) | (273.0, 7010.5) | (387.5, 5876.0) |  |  |  |
| **IgM GP2** |  |  |  | 0.74 | 0.894 |  |
| Mean (sd) | 57.4 (87.0) | 57.7 (84.7) | 56.8 (92.8) |  |  |  |
| Median (Q1,Q3) | 26.8 (12.6, 61.0) | 27.5 (13.0, 58.0) | 22.0 (12.5, 62.0) |  |  |  |
| Range (min, max) | (-27, 507) | (-13.0, 464.5) | (-27, 507) |  |  |  |
| **IgM HSP 90** |  |  |  | 0.71 | 0.879 |  |
| Mean (sd) | 44.3 (77.0) | 49.5 (85.4) | 33.4 (55.1) |  |  |  |
| Median (Q1,Q3) | 19.5 (6.0, 45.4) | 19 (6, 57) | 23.0 (6.0, 37.5) |  |  |  |
| Range (min, max) | (-57.5, 525.5) | (-3.5, 525.5) | (-57.5, 267.0) |  |  |  |
| **IgM HMG CoA** |  |  |  | 0.60 | 0.845 |  |
| Mean (sd) | 375.5 (475.1) | 387.3 (527.7) | 351.0 (346.1) |  |  |  |
| Median (Q1,Q3) | 222.8 (119.2, 405.1) | 217.5 (93.5, 415.5) | 234.0 (158.5, 352.0) |  |  |  |
| Range (min, max) | (27, 2979) | (27, 2979) | (43, 1370) |  |  |  |
| **IgM Collagen I C7774** |  |  |  | 0.83 | 0.936 |  |
| Mean (sd) | 99.7 (112.9) | 92.2 (84.2) | 115.4 (157.0) |  |  |  |
| Median (Q1,Q3) | 70.8 (38.9, 130.1) | 71.0 (41.0, 121.5) | 70.5 (37.5, 147.0) |  |  |  |
| Range (min, max) | (-338, 611) | (-125, 367) | (-338, 611) |  |  |  |
| **IgM dsDNA genomic** |  |  |  | 0.15 | 0.484 |  |
| Mean (sd) | 1391.9 (1722.9) | 1256.7 (1571.1) | 1673.3 (1996.6) |  |  |  |
| Median (Q1,Q3) | 865.8 (492.2, 1533.0) | 827 (461, 1451) | 984.5 (536.5, 1745.0) |  |  |  |
| Range (min, max) | (93.5, 11407.5) | (93.5, 11407.5) | (167.5, 10381.5) |  |  |  |
| **IgM PDH** |  |  |  | 0.15 | 0.484 |  |
| Mean (sd) | 109.1 (98.9) | 99.9 (91.6) | 128.3 (111.7) |  |  |  |
| Median (Q1,Q3) | 79.0 (53.5, 118.0) | 72.0 (53.5, 108.0) | 89.0 (53.5, 146.0) |  |  |  |
| Range (min, max) | (19.0, 585.5) | (19.0, 585.5) | (20.5, 452.5) |  |  |  |
| **IgM SmD1** |  |  |  | 0.55 | 0.835 |  |
| Mean (sd) | 266.2 (309.1) | 229.0 (163.5) | 343.6 (484.0) |  |  |  |
| Median (Q1,Q3) | 206.5 (127.6, 299.0) | 208.0 (132.5, 286.5) | 196.5 (107.5, 341.5) |  |  |  |
| Range (min, max) | (37.5, 2717.5) | (37.5, 991.0) | (58.0, 2717.5) |  |  |  |
| **IgM SmD** |  |  |  | 0.90 | 0.959 |  |
| Mean (sd) | 367.8 (321.1) | 331.5 (168.3) | 443.2 (505.1) |  |  |  |
| Median (Q1,Q3) | 301.5 (214.0, 424.4) | 317.5 (214.0, 411.0) | 301.0 (189.5, 462.0) |  |  |  |
| Range (min, max) | (71, 2958) | (71, 829) | (88, 2958) |  |  |  |
| **IgM Mi 2** |  |  |  | 0.17 | 0.512 |  |
| Mean (sd) | 106.7 (116.1) | 97.0 (106.0) | 126.9 (134.1) |  |  |  |
| Median (Q1,Q3) | 67.0 (33.2, 124.2) | 57.5 (31.0, 110.0) | 78.0 (37.5, 173.0) |  |  |  |
| Range (min, max) | (5.5, 590.0) | (5.5, 516.0) | (12, 590) |  |  |  |
| **IgM Ro SS A 52 kDa human recombinant** |  |  |  | 0.31 | 0.677 |  |
| Mean (sd) | 245.7 (764.5) | 276.2 (890.0) | 182.3 (394.9) |  |  |  |
| Median (Q1,Q3) | 42.2 (18.4, 178.5) | 30.5 (18.0, 163.0) | 92.0 (28.5, 220.5) |  |  |  |
| Range (min, max) | (4.5, 7191.0) | (4.5, 7191.0) | (4.5, 2396.5) |  |  |  |
| **IgM CENP A** |  |  |  | 0.77 | 0.904 |  |
| Mean (sd) | 314.8 (186.6) | 306.6 (162.1) | 331.8 (231.0) |  |  |  |
| Median (Q1,Q3) | 282.0 (208.6, 374.6) | 287 (211, 365) | 242.0 (208.0, 376.5) |  |  |  |
| Range (min, max) | (101, 1276) | (101, 1276) | (103.5, 1271.0) |  |  |  |
| **IgM Carbonic Anhydrase** |  |  |  | 0.52 | 0.82 |  |
| Mean (sd) | 647.3 (219.1) | 651.5 (213.5) | 638.6 (233.0) |  |  |  |
| Median (Q1,Q3) | 616.8 (491.8, 786.8) | 639.5 (534.0, 783.0) | 579.0 (450.0, 810.5) |  |  |  |
| Range (min, max) | (281, 1321) | (281, 1321) | (347.0, 1143.5) |  |  |  |
| **IgM Bovine Histone H2b F2b** |  |  |  | 0.09 | 0.416 |  |
| Mean (sd) | 1079.1 (1683.7) | 834.2 (1008.7) | 1588.7 (2521.0) |  |  |  |
| Median (Q1,Q3) | 451.0 (231.6, 1256.2) | 418.5 (213.5, 1092.5) | 701.5 (301.5, 1828.0) |  |  |  |
| Range (min, max) | (45.5, 13188.5) | (45.5, 5615.0) | (93.5, 13188.5) |  |  |  |
| **IgM Recombinant Histone H2A hu** |  |  |  | 0.75 | 0.894 |  |
| Mean (sd) | 830.5 (876.3) | 819.6 (911.9) | 853.3 (808.7) |  |  |  |
| Median (Q1,Q3) | 508.8 (318.2, 1016.5) | 524.5 (323.5, 960.0) | 463.0 (292.0, 1174.5) |  |  |  |
| Range (min, max) | (93, 5995) | (93, 5995) | (147.5, 3783.0) |  |  |  |
| **IgM Human IgM** |  |  |  | 0.98 | 0.987 |  |
| Mean (sd) | 26022.1 (10440.0) | 26392.0 (10926.4) | 25252.4 (9443.6) |  |  |  |
| Median (Q1,Q3) | 24363.0 (16724.6, 31738.6) | 23839.5 (16723.0, 38847.0) | 24979.0 (18299.5, 29893.0) |  |  |  |
| Range (min, max) | (7462.5, 45734.5) | (12543.0, 45734.5) | (7462.5, 44538.5) |  |  |  |
| **IgM HCEC total SDS** |  |  |  | 0.66 | 0.852 |  |
| Mean (sd) | 1630.8 (938.6) | 1570.3 (825.5) | 1756.7 (1141.3) |  |  |  |
| Median (Q1,Q3) | 1467.0 (1097.9, 1830.1) | 1466.0 (1109.5, 1809.5) | 1523.5 (1087.0, 1984.0) |  |  |  |
| Range (min, max) | (525.0, 5951.5) | (663, 5723) | (525.0, 5951.5) |  |  |  |
| **IgM Asparaginyl tRNA Synthetase** |  |  |  | 0.58 | 0.839 |  |
| Mean (sd) | 233.1 (570.8) | 226.9 (659.0) | 246.0 (324.9) |  |  |  |
| Median (Q1,Q3) | 101.2 (49.1, 218.2) | 101.5 (49.0, 202.5) | 100.5 (57.5, 258.5) |  |  |  |
| Range (min, max) | (6, 5769) | (6.5, 5769.0) | (6.0, 1384.5) |  |  |  |
| **IgM LKM 1 hp** |  |  |  | 0.23 | 0.614 |  |
| Mean (sd) | 130.1 (138.9) | 123.4 (143.7) | 144.0 (129.3) |  |  |  |
| Median (Q1,Q3) | 101.5 (50.5, 163.6) | 86.5 (47.5, 142.0) | 103.5 (68.5, 181.5) |  |  |  |
| Range (min, max) | (9.0, 1160.5) | (9.0, 1160.5) | (20.0, 697.5) |  |  |  |
| **IgM tTG baculovirus** |  |  |  | 0.13 | 0.449 |  |
| Mean (sd) | 88.2 (162.2) | 66.6 (107.0) | 133.1 (235.3) |  |  |  |
| Median (Q1,Q3) | 31.5 (9.5, 102.4) | 30.5 (6.5, 79.0) | 43.5 (15.0, 133.5) |  |  |  |
| Range (min, max) | (-7.0, 1118.5) | (-7, 727) | (0.0, 1118.5) |  |  |  |
| **IgM Vimentin** |  |  |  | 0.53 | 0.823 |  |
| Mean (sd) | 8.0 (17.2) | 7.5 (12.0) | 8.9 (25.0) |  |  |  |
| Median (Q1,Q3) | 3.5 (0.0, 9.5) | 4.5 (0.0, 10.0) | 3.0 (0.0, 8.5) |  |  |  |
| Range (min, max) | (-7.5, 149.0) | (-7.5, 57.5) | (-7.5, 149.0) |  |  |  |
| **IgM Alpha elastin** |  |  |  | 0.90 | 0.959 |  |
| Mean (sd) | 214.2 (734.6) | 239.3 (858.7) | 161.9 (364.6) |  |  |  |
| Median (Q1,Q3) | 58.2 (25.9, 160.4) | 57.5 (25.0, 158.5) | 74 (32, 161) |  |  |  |
| Range (min, max) | (3.5, 7418.0) | (3.5, 7418.0) | (4.5, 2225.0) |  |  |  |
| **IgM Troponin T** |  |  |  | 0.91 | 0.959 |  |
| Mean (sd) | 270.4 (223.5) | 273.8 (246.8) | 263.3 (167.9) |  |  |  |
| Median (Q1,Q3) | 215.5 (137.2, 296.0) | 217 (136, 293) | 206.0 (152.5, 360.0) |  |  |  |
| Range (min, max) | (68.0, 1501.5) | (68.0, 1501.5) | (89.5, 733.5) |  |  |  |
| **IgM Collagen IV C5533** |  |  |  | 0.65 | 0.852 |  |
| Mean (sd) | 79.9 (84.3) | 78.4 (80.4) | 83.2 (92.9) |  |  |  |
| Median (Q1,Q3) | 56.2 (26.6, 105.5) | 56.5 (23.5, 111.5) | 56.0 (31.5, 104.0) |  |  |  |
| Range (min, max) | (-87.0, 472.5) | (-87, 362) | (-53.0, 472.5) |  |  |  |
| **IgM GBM diss** |  |  |  | 0.46 | 0.781 |  |
| Mean (sd) | 95.9 (84.0) | 92.6 (83.6) | 102.7 (85.5) |  |  |  |
| Median (Q1,Q3) | 71.2 (37.6, 123.5) | 68.0 (37.5, 112.0) | 72.5 (38.0, 130.5) |  |  |  |
| Range (min, max) | (3.0, 435.5) | (3.0, 435.5) | (12.5, 318.5) |  |  |  |
| **IgM Cardiolipin C1649** |  |  |  | 0.88 | 0.957 |  |
| Mean (sd) | 69.1 (125.8) | 78.3 (145.4) | 50.1 (66.8) |  |  |  |
| Median (Q1,Q3) | 29.0 (9.5, 58.4) | 27 (8, 60) | 32.0 (12.0, 55.5) |  |  |  |
| Range (min, max) | (-12.0, 729.5) | (-12.0, 729.5) | (2.5, 331.0) |  |  |  |
| **IgM Collagen VI C7521** |  |  |  | 0.66 | 0.852 |  |
| Mean (sd) | 78.2 (88.9) | 81.1 (100.9) | 72.2 (56.9) |  |  |  |
| Median (Q1,Q3) | 55.0 (23.6, 112.4) | 49.5 (23.0, 112.5) | 71.0 (28.5, 108.0) |  |  |  |
| Range (min, max) | (-170.5, 580.0) | (-170.5, 580.0) | (-85.5, 196.5) |  |  |  |
| **IgM PM Scl 100** |  |  |  | 0.29 | 0.659 |  |
| Mean (sd) | 812.1 (720.5) | 692.4 (490.6) | 1061.0 (1013.4) |  |  |  |
| Median (Q1,Q3) | 623.8 (461.4, 864.1) | 644.0 (454.0, 817.5) | 591.5 (482.0, 1064.0) |  |  |  |
| Range (min, max) | (254.0, 5217.5) | (254.0, 4353.5) | (283.0, 5217.5) |  |  |  |
| **IgM snRNP 68** |  |  |  | 0.16 | 0.492 |  |
| Mean (sd) | 156.4 (245.4) | 124.8 (132.9) | 222.3 (381.0) |  |  |  |
| Median (Q1,Q3) | 88.0 (50.1, 171.6) | 80.0 (46.5, 159.5) | 104.0 (54.0, 176.5) |  |  |  |
| Range (min, max) | (7.5, 2127.0) | (7.5, 743.5) | (22.5, 2127.0) |  |  |  |
| **IgM CENP B** |  |  |  | 0.37 | 0.717 |  |
| Mean (sd) | 531.7 (803.7) | 453.6 (597.7) | 694.3 (1110.5) |  |  |  |
| Median (Q1,Q3) | 257.2 (122.0, 626.8) | 260.5 (121.5, 587.5) | 254.0 (187.5, 714.5) |  |  |  |
| Range (min, max) | (-40, 5637) | (-40.0, 4340.5) | (27, 5637) |  |  |  |
| **IgM Human IgE** |  |  |  | 0.98 | 0.987 |  |
| Mean (sd) | 340.1 (298.3) | 333.5 (239.2) | 353.8 (397.7) |  |  |  |
| Median (Q1,Q3) | 264.2 (169.8, 407.2) | 262.0 (169.5, 427.0) | 266.5 (181.5, 379.0) |  |  |  |
| Range (min, max) | (74.5, 2487.5) | (74.5, 1121.0) | (123.5, 2487.5) |  |  |  |
| **IgM La SS B Antigens Immunovision** |  |  |  | **0.04** | 0.314 |  |
| Mean (sd) | 226.4 (264.7) | 197.0 (244.0) | 287.7 (297.5) |  |  |  |
| Median (Q1,Q3) | 145.8 (69.9, 257.5) | 137.5 (64.5, 205.0) | 173 (93, 316) |  |  |  |
| Range (min, max) | (14.5, 1737.0) | (14.5, 1737.0) | (40.5, 1162.5) |  |  |  |
| IgM Histone H4 1 103 aa |  |  |  | 0.20 | 0.548 |  |
| Mean (sd) | 244.1 (360.8) | 201.8 (257.9) | 332.1 (506.4) |  |  |  |
| Median (Q1,Q3) | 127.5 (67.6, 229.4) | 125.0 (62.0, 215.5) | 142.0 (78.5, 270.5) |  |  |  |
| Range (min, max) | (18, 2334) | (18.0, 1528.5) | (27, 2334) |  |  |  |
| **IgM whole mouse IgG** |  |  |  | 0.29 | 0.659 |  |
| Mean (sd) | 164.1 (250.0) | 150.7 (207.4) | 191.9 (322.8) |  |  |  |
| Median (Q1,Q3) | 99.5 (56.1, 175.2) | 98.5 (48.5, 174.5) | 100.5 (69.0, 175.5) |  |  |  |
| Range (min, max) | (18, 1983) | (18, 1609) | (22, 1983) |  |  |  |
| **IgM Desmin** |  |  |  | 0.18 | 0.532 |  |
| Mean (sd) | 323.6 (286.6) | 310.5 (206.7) | 350.9 (408.1) |  |  |  |
| Median (Q1,Q3) | 258.8 (172.4, 353.8) | 272.5 (190.5, 378.5) | 209.5 (160.5, 333.0) |  |  |  |
| Range (min, max) | (31, 1883) | (55.0, 1313.5) | (31, 1883) |  |  |  |
| **IgM beta 2 GPI non recombinant Human** |  |  |  | 0.58 | 0.839 |  |
| Mean (sd) | 90.0 (103.1) | 94.4 (117.4) | 80.7 (64.2) |  |  |  |
| Median (Q1,Q3) | 68.2 (47.6, 105.5) | 72.5 (46.5, 107.5) | 64.5 (48.5, 85.0) |  |  |  |
| Range (min, max) | (-37, 987) | (10, 987) | (-37, 347) |  |  |  |
| **IgM Nucleosome non recombinant bovine** |  |  |  | 0.64 | 0.852 |  |
| Mean (sd) | 429.5 (479.1) | 399.1 (426.0) | 492.9 (575.5) |  |  |  |
| Median (Q1,Q3) | 256.8 (151.0, 511.4) | 247.0 (154.0, 434.5) | 270.0 (125.0, 521.5) |  |  |  |
| Range (min, max) | (35.5, 2438.5) | (35.5, 2346.0) | (50.0, 2438.5) |  |  |  |
| **IgM BCOADC E2** |  |  |  | 0.75 | 0.894 |  |
| Mean (sd) | 326.5 (393.9) | 341.9 (450.1) | 294.3 (240.2) |  |  |  |
| Median (Q1,Q3) | 212.0 (109.4, 332.9) | 210.0 (108.5, 327.0) | 214 (120, 417) |  |  |  |
| Range (min, max) | (32, 2691) | (32, 2691) | (45.5, 984.5) |  |  |  |
| **IgM human fgl2** |  |  |  | 0.56 | 0.835 |  |
| Mean (sd) | 69.2 (62.3) | 72.3 (63.3) | 62.6 (60.6) |  |  |  |
| Median (Q1,Q3) | 54.0 (34.0, 84.2) | 57.5 (34.5, 85.0) | 45.5 (32.0, 82.0) |  |  |  |
| Range (min, max) | (-98.0, 400.5) | (-0.5, 400.5) | (-98, 289) |  |  |  |
| **IgM B1AR** |  |  |  | 0.29 | 0.661 |  |
| Mean (sd) | 197.5 (327.5) | 231.3 (383.5) | 127.4 (136.3) |  |  |  |
| Median (Q1,Q3) | 93.0 (36.0, 198.2) | 112.0 (38.5, 212.5) | 84.0 (35.5, 160.5) |  |  |  |
| Range (min, max) | (3.5, 2572.0) | (3.5, 2572.0) | (7.5, 566.5) |  |  |  |
| **IgM Grp78 BiP** |  |  |  | 0.46 | 0.781 |  |
| Mean (sd) | 258.8 (499.4) | 211.2 (241.3) | 357.8 (803.2) |  |  |  |
| Median (Q1,Q3) | 133.5 (75.1, 239.0) | 131.0 (77.5, 211.0) | 144.5 (63.5, 288.5) |  |  |  |
| Range (min, max) | (-108, 4890) | (17.0, 1201.5) | (-108, 4890) |  |  |  |
| **IgM HSP 40** |  |  |  | 0.52 | 0.818 |  |
| Mean (sd) | 320.3 (421.5) | 317.6 (384.1) | 326.0 (496.2) |  |  |  |
| Median (Q1,Q3) | 185.8 (102.2, 342.6) | 203 (120, 340) | 157.5 (88.0, 343.5) |  |  |  |
| Range (min, max) | (26, 2676) | (31.5, 1985.0) | (26, 2676) |  |  |  |
| **IgM Enolase** |  |  |  | **0.03** | 0.277 |  |
| Mean (sd) | 109.3 (229.8) | 90.5 (219.7) | 148.6 (248.0) |  |  |  |
| Median (Q1,Q3) | 53.5 (27.6, 92.2) | 49.0 (26.0, 85.5) | 61.5 (39.0, 156.5) |  |  |  |
| Range (min, max) | (6, 1873) | (6, 1873) | (11.0, 1427.5) |  |  |  |
| **IgM ssDNA** |  |  |  | 0.33 | 0.689 |  |
| Mean (sd) | 2135.9 (2342.0) | 1968.8 (2164.4) | 2483.6 (2672.7) |  |  |  |
| Median (Q1,Q3) | 1391.2 (753.4, 2718.1) | 1339.5 (734.5, 2621.5) | 1433.5 (836.5, 3138.0) |  |  |  |
| Range (min, max) | (122.0, 15100.5) | (122.0, 15100.5) | (285.5, 13367.5) |  |  |  |
| **IgM Fib I** |  |  |  | 0.30 | 0.67 |  |
| Mean (sd) | 557.4 (1513.5) | 558.6 (1787.2) | 554.9 (668.8) |  |  |  |
| Median (Q1,Q3) | 271.2 (167.2, 414.9) | 259.5 (165.5, 369.0) | 285.5 (190.0, 564.5) |  |  |  |
| Range (min, max) | (70, 15731) | (70, 15731) | (90, 3099) |  |  |  |
| **IgM Ribo P0** |  |  |  | 0.66 | 0.852 |  |
| Mean (sd) | 445.7 (507.4) | 439.1 (556.3) | 459.5 (393.2) |  |  |  |
| Median (Q1,Q3) | 304.0 (207.2, 542.0) | 311.0 (204.5, 546.0) | 286.0 (231.5, 422.5) |  |  |  |
| Range (min, max) | (71.5, 4686.0) | (71.5, 4686.0) | (132.5, 1485.5) |  |  |  |
| **IgM Nucleolin** |  |  |  | 0.22 | 0.581 |  |
| Mean (sd) | 553.9 (548.6) | 499.5 (486.5) | 667.1 (652.3) |  |  |  |
| Median (Q1,Q3) | 393.0 (226.6, 676.2) | 314.0 (199.5, 669.5) | 414.5 (252.0, 741.0) |  |  |  |
| Range (min, max) | (57.5, 3461.5) | (57.5, 3461.5) | (102.5, 3040.0) |  |  |  |
| **IgM SmD3** |  |  |  | 0.54 | 0.823 |  |
| Mean (sd) | 408.0 (237.2) | 405.6 (197.6) | 413.0 (306.9) |  |  |  |
| Median (Q1,Q3) | 384.8 (232.9, 524.5) | 411.5 (230.5, 538.5) | 365.0 (242.0, 469.5) |  |  |  |
| Range (min, max) | (71.0, 1885.5) | (71, 843) | (105.5, 1885.5) |  |  |  |
