# Supplementary material for: Autoimmune PaneLs as PrEdictors of Toxicity in Patients TReated with Immune Checkpoint InhibiTors (ALERT)
Source: J Exp Clin Cancer Res. 2023 Oct 21;42:276. doi: 10.1186/s13046-023-02851-6 (PMC10589949; doi:10.1186/s13046-023-02851-6)
Supplement: Supplementary file 4 — Additional file 4: Supplementary Table 4. Autoantibodies with significantly different mean MFI levels at baseline (pre-ICI) in patients with and without irAEs. [file 13046_2023_2851_MOESM4_ESM.docx]

**Supplementary Table 4. Autoantibodies with significantly different mean MFI levels at baseline (pre-ICI) in patients with and without irAEs**

| **Autoantibody** | **Patients without irAEs G≥2 (mean MFI±DS)** | **Patients with irAEs G≥2 (mean MFI±DS)** | **P value** |
| --- | --- | --- | --- |
| **IgG Bovin Histone H4 and H2A** | 246.6 (±292.2) | 467.8 (±706.9) | 0.04 |
| **IgG Insulin** | -65.6 (±20.1) | -47.7 (±63.9) | 0.05 |
| **IgG MPO** | -33.6 (±120.2) | -12.9 (±103.7) | 0.03 |
| **IgG SPLUNC2** | 187.2 (±118.2) | 144.8 (±74.6) | 0.03 |
| **IgG DNA Topoisomerase I Scl 70 non recombinant bovine** | 79.7 (104.2) | 215.1 (±371.7) | 0.04 |
| **IgG Sm non recombinant bovine** | 218.0 (±549.1) | 241.6 (±273.8) | 0.01 |
| **IgG Collagen III C4407** | 46.8 (±43.4) | 85.8 (±82.5) | 0.008 |
| **IgG Ebna peptide** | 848.9 (±1911.4) | 346.8 (±753.3) | 0.02 |
| **IgG Ro SS A 60kD recombinant** | 349.8 (±1928.9) | 436.2 (±2332.4) | 0.02 |
| **IgG Actin Bovine** | -9.5 (±30.1) | 15.8 (±82.3) | 0.02 |
| **IgG Aggrecan recombinant** | -11.5 (±138.0) | -15.2 (±35.8) | 0.05 |
| **IgG BPI** | 362.9 (±1510.7) | 102.5 (±220.7) | 0.05 |
| **IgG HCEC memb** | 213.4 (±186.7) | 308.2 (±223.2) | 0.008 |
| **IgG Glycyl tRNA Synthetase EJ** | 89.2 (±542.0) | 331.6 (±1229.9) | 0.02 |
| **IgG LCI** | 167.9 (±372.1) | 471.6 (±1085.5) | 0.003 |
| **IgG alphaB crystallin** | 406.8 (±1061.4) | 165.5 (±182.6) | 0.04 |
| **IgG snRNP C** | 1003.8 (±3172.4) | 1102.4 (±1216.8) | 0.01 |
| **IgG Mucarinic Rceptor 3** | 110.0 (±851.0) | 159.3 (±312.4) | <0.001 |
| **IgG Bovine Histone H3** | 331.4 (±419.3) | 722.0 (±1217.3) | 0.03 |
| **IgG RNP Sm non recombinant bovine** | 154.0 (±558.2) | 135.7 (±174.5) | 0.02 |
| **IgG PDC E2** | 448.6 (±1040.6) | 470.2 (±679.3) | 0.009 |
| **IgG Collagen V C3657** | 120.7 (±125.5) | 612.4 (±2163.9) | <0.001 |
| **IgG M2** | 260.7 (±690.6) | 306.1 (±423.0) | 0.01 |
| **IgG Alpha KGDH** | 36.5 (±115.6) | 57.3 (±73.1) | 0.006 |
| **IgG PM Scl 75** | 541.9 (±1468.8) | 788.1 (±1686.1) | 0.005 |
| **IgG whole histones** | 678.7 (±674.5) | 1267.7 (±1765.5) | 0.002 |
| **IgG human IgG Fc** | 54725.6 (±8391.0) | 50648.4 (±9673.7) | 0.03 |
| **IgG Collagen I C7774** | 159.8 (±174.5) | 230.9 (±183.0) | 0.01 |
| **IgG PDH** | 123.9 (±125.5) | 169.6 (±131.1) | 0.02 |
| **IgG SmD** | 419.5 (±922.8) | 491.6 (±665.8) | 0.04 |
| **IgG Bovine Histone H2b F2b** | 302.5 (±503.3) | 566.1 (±762.1) | <0.001 |
| **IgG CENP B** | 574.7 (±1500.9) | 1223.3 (±2373.3) | 0.003 |
| **IgG Histone H4 1 103 aa** | 571.9 (±498.4) | 1027.6 (±1246.7) | 0.02 |
| **IgG beta 2 GPI non recombinant Human** | -1.5 (±36.4) | 16.4 (±35.9) | 0.007 |
| **IgG Nucleosome non recombinant bovine** | 242.0 (±366.9) | 422.3 (±482.0) | 0.006 |
| **IgG BCOADC E2** | 255.3 (±623.7) | 271.3 (±327.4) | 0.04 |
| **IgG Ribo P0** | 876.9 (±6081.7) | 359.0 (±626.1) | <0.001 |
| **IgG Nucleolin** | 226.5 (±195.9) | 281.2 (±182.1) | 0.02 |
| **IgG SmD3** | 97.4 (±260.3) | 162.6 (±278.0) | 0.009 |
| **IgM Tropoelastin** | 1085.1 (±748.1) | 830.9 (±838.2) | 0.002 |
| **IgM PBS** | 16.0 (±29.3) | 14.2 (±52.4) | 0.01 |
| **IgM BPI** | 77.5 (±106.7) | 150.6 (±322.5) | 0.003 |
| **IgM HCEC memb** | 192.3 (±130.8) | 287.8 (±221.2) | 0.03 |
| **IgM PM Scl 75** | 261.4 (±418.7) | 436.7 (±551.3) | 0.05 |
| **IgM whole histones** | 708.0 (±803.4) | 1321.5 (±1875.0) | 0.03 |
| **IgM La SS B Antigens Immunovision** | 197.0 (±244.0) | 287.7 (±297.5) | 0.04 |
| **IgM Enolase** | 90.5 (±219.7) | 148.6 (±248.0) | 0.03 |
