# Supplementary material for: Autoimmune PaneLs as PrEdictors of Toxicity in Patients TReated with Immune Checkpoint InhibiTors (ALERT)
Source: J Exp Clin Cancer Res. 2023 Oct 21;42:276. doi: 10.1186/s13046-023-02851-6 (PMC10589949; doi:10.1186/s13046-023-02851-6)
Supplement: Supplementary file 5 — Additional file 5: Supplementary Table 5. AutoAbs Distribution by History of Autoimmune Disease Status [file 13046_2023_2851_MOESM5_ESM.pdf]

Supplementary table 5: AutoAbs Distribution by History of Autoimmune Disease Status

There were eight patients with history of autoimmune disease. The distribution of each measure are compared by history status. Both the unadjusted and adjusted p-values are reported

Measure	Median, History of Autoimm	Median, No History of Aut	Unadjusted	Adjusted P-V
IgM_SP_D	657.25	456	0.258	1
IgM_Bovin_Histone_H4_and_H2A	522.5	411.5	0.591	1
IgM_Human_core_histones	304	191.25	0.366	1
IgM_Human_IgG	130.5	121.25	0.868	1
IgM_HCEC_cytop	331.5	290.5	0.583	1
IgM_MDA5	47.5	49.25	0.799	1
IgM_Gliadin	423.75	488.25	0.686	1
IgM_tTG_E_coli	31.5	24	0.743	1
IgM_Myosin_Bind_Protein_C	278.5	175	0.557	1
IgM_Tropoelastin	836.5	813	0.912	1
IgM_Insulin	28.75	18	0.868	1
IgM_Tropomyosin	632.5	479.75	0.415	1
IgM_Myosin_M1636	12.75	8	0.907	1
IgM_Jo_1	150.25	82.5	0.384	1
IgM_Ribo_P1	244.25	227.5	0.842	1
IgM_MPO	440.75	386	0.471	1
IgM_Scl_70_Full	183.25	187	0.499	1
IgM_Scl_70_trunc	274.25	234.25	0.572	1
IgM_SPLUNC2	108	73.25	0.136	1
IgM_Sm_Antigens	225	175.75	0.572	1
IgM_Histone_H3_1_136_aa	312.75	235.75	0.710	1
IgM_mouse_IgM	4115.25	3018	0.332	1
IgM_Beta_galactosidase_2B	428.5	259.5	0.256	1
IgM_DNA_Topoisomerase_I_Scl_70_non_recombinant_bovine	103.5	91.75	0.782	1
IgM_Sm_non_recombinant_bovine	452.5	432.5	0.890	1
IgM_OGDC_E2	116.5	111.25	0.846	1
IgM_mouse_fgl2	72.75	65	1.000	1
IgM_HSP_70	169	147.75	0.929	1
IgM_HSP_60	142	111.5	0.912	1
IgM_Collagen_III_C4407	90.75	68.25	0.829	1
IgM_Heparin	8.5	7.5	0.677	1
IgM_Ebna_peptide	1.25	0	0.172	1
IgM_Fib_I_S	169.75	131.25	0.925	1
IgM_PL_12	548.5	421.5	0.279	1
IgM_La_SS_B_Recombinant_human_diarect	260.25	138	0.192	1
IgM_Ku_p70_p80	79.5	52	0.246	1
IgM_empty	-1	-0.5	0.704	1
IgM_Human_H1_Chromatin	330.5	240.25	0.837	1
IgM_Human_LGALS3_Galectin_3	-1.5	-0.5	0.052	1
IgM_Human_IgA	167	158.5	0.502	1
IgM_kidney_endothelial_cell_lysate	484.75	402.5	0.169	1
IgM_MYH6	399.5	285.75	0.300	1
IgM_Ro_SS_A_60kD_recombinant	76.25	53.5	0.256	1
IgM_Sp100	158.5	153.75	0.850	1
IgM_B2AR	93.75	63.75	0.824	1
IgM_AT1	24.5	21.25	0.881	1
IgM_ICAM_1	12	8	0.502	1
IgM_HSP_27	196	216	1.000	1
IgM_Actin_Bovine	27	17	0.337	1
IgM_Actin_Rabbit	23	16	0.829	1
IgM_Aggrecan_recombinant	26.25	4.5	0.056	1
IgM_PBS	5.75	3	0.799	1
IgM_Thyrogobulin	62.5	59	0.885	1
IgM_BPI	80.75	55.5	0.378	1
IgM_SCGB1A1	261.25	158.75	0.769	1
IgM_Bovin_Histone_subclass_F1	627.25	553	0.629	1
IgM_Histone_H2B	1096.25	656.25	0.437	1
IgM_human_IgG_F_ab_2	85.25	96.75	0.769	1
IgM_HCEC_memb	162.5	172.25	0.807	1
IgM_Glycyl_tRNA_Synthetase_EJ	92	61.5	0.334	1
IgM_Nup62	85	60.75	0.837	1
IgM_LCI	112.75	71.75	0.474	1
IgM_alphaB_crystallin	386.25	463.5	0.579	1

IgM_Beta_2_Glyc_recombinant_human	192	118	0.256	1
IgM_Ox_LDL	91.5	60.5	0.244	1
IgM_Proteoglycan	25.5	18.25	0.474	1
IgM_LG3	163.25	218.5	0.991	1
IgM_Ribo_P2	18.75	14.75	0.991	1
IgM_SmD2	37.5	31.5	0.782	1
IgM_PR3	29.25	25.25	0.531	1
IgM_snRNP_C	346.25	353.75	0.485	1
IgM_snRNP_68_B_B	1257.25	1138.75	0.748	1
IgM_Mucarinic_Rceptor_3	98.75	87	0.777	1
IgM_Bovine_Histone_H3	2871	1491.5	0.409	1
IgM_Histone_H2A_H2B_dimers	579.5	430.5	0.454	1
IgM_mouse_IgG_F_ab_2	41	33.5	0.731	1
IgM_human_intestinal_smooth_muscle_cell	338	319.25	0.773	1
IgM_Intrinsic_Factor	136.5	87.25	0.235	1
IgM_RNP_Sm_non_recombinant_bovine	270.5	224.25	0.943	1
IgM_PDC_E2	188.25	96.25	0.189	1
IgM_dsDNA_plasmid	471.75	374.25	0.572	1
IgM_Measles	153	91.5	1.000	1
IgM_Laminin	20	11.75	0.641	1
IgM_Collagen_V_C3657	103.25	83.75	0.894	1
IgM_Porcine_Myosin_Heart	248.75	186.25	0.587	1
IgM_TUBA1B_protein	-0.5	1	0.424	1
IgM_Aldolase_Type_X	-1	-1.75	0.876	1
IgM_TPO	191	158.75	0.499	1
IgM_SRP54	578.25	344	0.216	1
IgM_PL_7	195.75	168.25	0.277	1
IgM_human_albumin	89.5	57	0.381	1
IgM_C1q_purified_non_recombinant	206.75	180.75	0.502	1
IgM_Human_LEDGF	424.75	397.25	0.222	1
IgM_mouse_IgG_Fc	106.5	82.25	0.925	1
IgM_Troponin_I	142.25	179.25	0.545	1
IgM_beta_2_GPI_non_recombinant_Bovine	57	55.5	0.842	1
IgM_Ro_SS_A_60_kD_non_recombinant_bovine	37.5	32.25	0.575	1
IgM_M2	264	173.5	0.396	1
IgM_M2AR	5.75	5.5	0.837	1
IgM_A1AR	23.25	6.75	0.077	1
IgM_Troponin_C	138	130.5	0.390	1
IgM_HSP_47	88.25	109.5	0.481	1
IgM_alpha_Actinin	6.25	5.5	1.000	1
IgM_Alpha_KGDH	23.75	24.25	0.842	1
IgM_Fib_IV	19.25	24.25	0.868	1
IgM_PCNA	129.25	73.5	0.297	1
IgM_snRNP_A	323.5	415.25	0.387	1
IgM_PM_Scl_75	169.5	148.25	0.553	1
IgM_human_C1q_Abcam	8804.5	8269.75	0.560	1
IgM_whole_histones	708.25	478.75	0.481	1
IgM_human_nucleosome	73.25	66.5	0.579	1
IgM_human_IgG_Fc	106.5	122.75	0.872	1
IgM_HCEC_total_Triton	1245	1160.75	0.769	1
IgM_TIF1_gamma	249.25	248.75	0.782	1
IgM_gp210	1129.75	1217.25	0.960	1
IgM_GP2	20.25	27.25	0.863	1
IgM_HSP_90	17	19.5	0.898	1
IgM_HMG_CoA	340.75	218	0.360	1
IgM_Collagen_I_C7774	85.75	70.25	0.881	1
IgM_dsDNA_genomic	927.5	831.25	0.502	1
IgM_PDH	100	75	0.346	1
IgM_SmD1	239.5	204.75	0.495	1
IgM_SmD	324.75	301.5	0.991	1
IgM_Mi_2	126	60.25	0.200	1
IgM_Ro_SS_A_52_kDa_human_recombinant	73.25	35.5	0.649	1
IgM_CENP_A	296	276	0.790	1
IgM_Carbonic_Anhydrase	634.25	609	0.960	1
IgM_Bovine_Histone_H2b_F2b	863.75	426.75	0.204	1
IgM_Recombinant_Histone_H2A_hu	858	486	0.454	1
IgM_Human_IgM	33262.25	24112	0.171	1
IgM_HCEC_total_SDS	1538	1467	0.833	1

IgM_Asparaginyl_tRNA_Synthetase	126.25	100.25	0.542	1
IgM_LKM_1_hp	102	99.5	0.960	1
IgM_tTG_baculovirus	40	31	0.481	1
IgM_Vimentin	4.75	3.5	0.837	1
IgM_Alpha_elastin	78.5	57.75	0.649	1
IgM_Troponin_T	188.5	216.5	0.502	1
IgM_Collagen_IV_C5533	70.75	54.25	0.665	1
IgM_GBM_diss	61.75	71.5	0.903	1
IgM_Cardiolipin_C1649	52.5	25.5	0.107	1
IgM_Collagen_VI_C7521	94	48.25	0.176	1
IgM_PM_Scl_100	666.25	602.25	0.553	1
IgM_snRNP_68	93.5	88	0.803	1
IgM_CENP_B	256.5	257.25	0.890	1
IgM_Human_IgE	342.5	251.5	0.198	1
IgM_La_SS_B_Antigens_Immunovision	143.25	145.75	0.765	1
IgM_Histone_H4_1_103_aa	159.5	126.75	0.402	1
IgM_whole_mouse_IgG	125	95.75	0.421	1
IgM_Desmin	203.5	259.75	0.390	1
IgM_beta_2_GPI_non_recombinant_Human	91.75	66.75	0.204	1
IgM_Nucleosome_non_recombinant_bovine	305	256.75	0.769	1
IgM_BCOADC_E2	275.5	207.75	0.428	1
IgM_human_fgl2	65.25	53.25	0.481	1
IgM_B1AR	172	91.25	0.176	1
IgM_Grp78_BiP	140	133.5	0.790	1
IgM_HSP_40	239.5	181.5	0.811	1
IgM_Enolase	73.5	51.75	0.424	1
IgM_ssDNA	1617.25	1331.5	0.606	1
IgM_Fib_I	342.5	266	0.591	1
IgM_Ribo_P0	331	304	0.934	1
IgM_Nucleolin	634.25	365.5	0.131	1
IgM_SmD3	425.75	382.75	0.598	1
IgG_SP_D	7119.5	5231	0.116	1
IgG_Bovin_Histone_H4_and_H2A	303.75	171.75	0.396	1
IgG_Human_core_histones	383	241.5	0.424	1
IgG_Human_IgG	35671.25	35503.5	0.752	1
IgG_HCEC_cytop	522.25	501.25	0.694	1
IgG_MDA5	-171	-114.5	0.516	1
IgG_Gliadin	1363	1969.5	0.114	1
IgG_tTG_E_coli	90.5	19.75	0.335	1
IgG_Myosin_Bind_Protein_C	759.25	520.5	0.743	1
IgG_Tropoelastin	625.5	366.25	0.040	1
IgG_Insulin	-59	-62.5	0.837	1
IgG_Tropomyosin	595.75	650.25	0.769	1
IgG_Myosin_M1636	-32.5	-34	0.610	1
IgG_Jo_1	-12.5	36	0.340	1
IgG_Ribo_P1	122.5	154.75	0.542	1
IgG_MPO	-24.5	-53.5	0.542	1
IgG_Scl_70_Full	77	141.25	0.181	1
IgG_Scl_70_trunc	75	169	0.097	1
IgG_SPLUNC2	191	148.25	0.454	1
IgG_Sm_Antigens	490.5	344	0.114	1
IgG_Histone_H3_1_136_aa	1871	820	0.081	1
IgG_mouse_IgM	132.5	152.5	0.960	1
IgG_Beta_galactosidase_2B	239.75	208.5	0.881	1
IgG_DNA_Topoisomerase_I_Scl_70_non_recombinant_bovine	60.5	55	0.731	1
IgG_Sm_non_recombinant_bovine	217.75	76.75	0.037	1
IgG_OGDC_E2	2	-10	0.653	1
IgG_mouse_fgl2	94.25	112.5	0.412	1
IgG_HSP_70	183.5	170.5	0.916	1
IgG_HSP_60	288.5	717.25	0.136	1
IgG_Collagen_III_C4407	54	47.75	0.842	1
IgG_Heparin	17.5	27.25	0.572	1
IgG_Ebna_peptide	69.5	127	0.649	1
IgG_Fib_I_S	166.75	166	0.591	1
IgG_PL_12	1261.75	1310	0.982	1
IgG_La_SS_B_Recombinant_human_diarect	103.5	175	0.421	1
IgG_Ku_p70_p80	5.5	41.75	0.033	1
IgG_empty	-0.5	-1	0.488	1

IgG_Human_H1_Chromatin	337	248.25	0.321	1
IgG_Human_LGALS3_Galectin_3	1.5	2	0.491	1
IgG_Human_IgA	1409.5	585.25	0.142	1
IgG_kidney_endothelial_cell_lysate	916.75	1017.25	0.686	1
IgG_MYH6	2879.25	2763.75	0.969	1
IgG_Ro_SS_A_60kD_recombinant	17	22.25	0.881	1
IgG_Sp100	74	170.25	0.372	1
IgG_B2AR	-9.75	1	0.114	1
IgG_AT1	28.75	35	0.714	1
IgG_ICAM_1	-23.75	-23.75	0.916	1
IgG_HSP_27	343.75	151	0.032	1
IgG_Actin_Bovine	-0.25	-4	0.973	1
IgG_Actin_Rabbit	-3.25	-21	0.418	1
IgG_Aggrecan_recombinant	-26.25	-27.25	0.657	1
IgG_PBS	-24.5	-39.5	0.233	1
IgG_Thyrogobulin	237.75	110.5	0.164	1
IgG_BPI	45	16.25	0.284	1
IgG_SCGB1A1	10355.5	7527.75	0.637	1
IgG_Bovin_Histone_subclass_F1	773.5	582	0.625	1
IgG_Histone_H2B	761.25	490.25	0.673	1
IgG_human_IgG_F_ab_2	5603.25	7162.5	0.622	1
IgG_HCEC_memb	219	185	0.842	1
IgG_Glycyl_tRNA_Synthetase_EJ	-25.75	4.75	0.208	1
IgG_Nup62	-20.5	7.25	0.625	1
IgG_LCI	39.25	86.25	0.181	1
IgG_alphaB_crystallin	113.5	133	0.246	1
IgG_Beta_2_Glyc_recombinant_human	50.5	44.5	0.478	1
IgG_Ox_LDL	182.25	210	0.499	1
IgG_Proteoglycan	-20	-21.25	0.811	1
IgG_LG3	16.75	47.25	0.180	1
IgG_Ribo_P2	160.75	94.25	0.297	1
IgG_SmD2	57.25	102	0.043	1
IgG_PR3	33.75	34.75	0.369	1
IgG_snRNP_C	338.25	452.25	0.246	1
IgG_snRNP_68_B_B	570.25	410.25	0.495	1
IgG_Mucarinic_Rceptor_3	16	-1.25	0.187	1
IgG_Bovine_Histone_H3	383.75	243.75	0.545	1
IgG_Histone_H2A_H2B_dimers	389	233.5	0.641	1
IgG_mouse_IgG_F_ab_2	61	69.75	0.424	1
IgG_human_intestinal_smooth_muscle_cell	305.75	247	0.246	1
IgG_Intrinsic_Factor	173.25	206.5	0.598	1
IgG_RNP_Sm_non_recombinant_bovine	86.75	38.25	0.354	1
IgG_PDC_E2	22.75	108.25	0.471	1
IgG_dsDNA_plasmid	406.5	145	0.244	1
IgG_Measles	112.5	106	0.960	1
IgG_Laminin	-20.75	-6.25	0.366	1
IgG_Collagen_V_C3657	159	91	0.598	1
IgG_Porcine_Myosin_Heart	71.5	126.25	0.265	1
IgG_TUBA1B_protein	-50.5	-49	0.855	1
IgG_Aldolase_Type_X	-73.75	-80	0.681	1
IgG_TPO	1530.75	514.75	0.208	1
IgG_SRP54	1093	288.75	0.181	1
IgG_PL_7	129	117.75	0.718	1
IgG_human_albumin	332.75	434	0.947	1
IgG_C1q_purified_non_recombinant	29.75	12.5	0.378	1
IgG_Human_LEDGF	907.75	873	0.799	1
IgG_mouse_IgG_Fc	184	101.75	0.334	1
IgG_Troponin_I	427.75	263	0.164	1
IgG_beta_2_GPI_non_recombinant_Bovine	-30.75	-29.75	0.951	1
IgG_Ro_SS_A_60_kD_non_recombinant_bovine	19.75	46.75	0.194	1
IgG_M2	118.5	57.25	0.872	1
IgG_M2AR	-12.5	-6.5	0.128	1
IgG_A1AR	-31	-33	0.752	1
IgG_Troponin_C	658.75	444	0.378	1
IgG_HSP_47	84.75	59.75	0.343	1
IgG_alpha_Actinin	2.25	0.25	0.665	1
IgG_Alpha_KGDH	17	10.25	0.610	1
IgG_Fib_IV	-42.5	-43	0.564	1

IgG_PCNA	-9	43.75	0.021	1
IgG_snRNP_A	23.75	135.25	0.171	1
IgG_PM_Scl_75	-115.75	141.5	0.033	1
IgG_human_C1q_Abcam	8917.75	9303.5	0.653	1
IgG_whole_histones	706	493	0.441	1
IgG_human_nucleosome	528.75	489.5	0.973	1
IgG_human_IgG_Fc	53059.25	54048.5	0.837	1
IgG_HCEC_total_Triton	767.75	769	0.890	1
IgG_TIF1_gamma	-43.25	9.75	0.028	1
IgG_gp210	436	242.25	0.454	1
IgG_GP2	39.25	-4.5	0.622	1
IgG_HSP_90	1.25	4	0.960	1
IgG_HMG_CoA	214.25	276.5	0.598	1
IgG_Collagen_I_C7774	136.75	116.5	0.846	1
IgG_dsDNA_genomic	858.25	539.25	0.447	1
IgG_PDH	70	86.75	0.606	1
IgG_SmD1	154.25	76	0.894	1
IgG_SmD	128.75	144.5	0.898	1
IgG_Mi_2	87.25	139.5	0.166	1
IgG_Ro_SS_A_52_kDa_human_recombinant	32	25.75	0.710	1
IgG_CENP_A	159.25	89.75	0.633	1
IgG_Carbonic_Anhydrase	1419.25	1300.25	0.349	1
IgG_Bovine_Histone_H2b_F2b	322.25	197.5	0.310	1
IgG_Recombinant_Histone_H2A_hu	637.25	477	0.516	1
IgG_Human_IgM	79.75	208	0.375	1
IgG_HCEC_total_SDS	790.25	567	0.326	1
IgG_Asparaginyl_tRNA_Synthetase	25.75	88.75	0.357	1
IgG_LKM_1_hp	20.5	31	0.531	1
IgG_tTG_baculovirus	-30.25	-32	0.731	1
IgG_Vimentin	-44.5	-44	0.461	1
IgG_Alpha_elastin	-18	0.75	0.471	1
IgG_Troponin_T	385.5	491.25	0.938	1
IgG_Collagen_IV_C5533	10.75	10.5	0.850	1
IgG_GBM_diss	-26	-34.5	0.718	1
IgG_Cardiolipin_C1649	93.75	34.5	0.405	1
IgG_Collagen_VI_C7521	-1	-6.25	0.859	1
IgG_PM_Scl_100	642.75	697.5	0.943	1
IgG_snRNP_68	158.25	92.25	0.991	1
IgG_CENP_B	338.25	235.25	0.855	1
IgG_Human_IgE	706.5	650.75	0.531	1
IgG_La_SS_B_Antigens_Immunovision	631.25	369.25	0.591	1
IgG_Histone_H4_1_103_aa	862.5	458.5	0.204	1
IgG_whole_mouse_IgG	256.25	174.25	0.441	1
IgG_Desmin	21.5	-16	0.956	1
IgG_beta_2_GPI_non_recombinant_Human	9.5	-1.5	0.412	1
IgG_Nucleosome_non_recombinant_bovine	287	148	0.275	1
IgG_BCOADC_E2	81.25	99.5	0.735	1
IgG_human_fgl2	189.25	205	0.208	1
IgG_B1AR	-25	-13.5	0.159	1
IgG_Grp78_BiP	685.75	605.25	0.409	1
IgG_HSP_40	72.5	78	0.657	1
IgG_Enolase	77.75	73.75	0.872	1
IgG_ssDNA	1262.25	1146	0.710	1
IgG_Fib_I	512.75	624.75	0.192	1
IgG_Ribo_P0	115	105.25	0.991	1
IgG_Nucleolin	296.25	187	0.251	1
IgG_SmD3	111	32.5	0.287	1
