# Supplementary material for: Autoimmune PaneLs as PrEdictors of Toxicity in Patients TReated with Immune Checkpoint InhibiTors (ALERT)
Source: J Exp Clin Cancer Res. 2023 Oct 21;42:276. doi: 10.1186/s13046-023-02851-6 (PMC10589949; doi:10.1186/s13046-023-02851-6)
Supplement: Supplementary file 7 — Additional file 7: Supplementary Table 7. Selected cases of patients in which a significant elevation of autoAb correlated with organ-related toxicity. [file 13046_2023_2851_MOESM7_ESM.docx]

**Supplementary Table 7. Selected cases of patients in which a significant elevation of autoAb correlated with organ-related toxicity.**

| **Tumor type** | **irAE** | **Ab of interest** | **MFI at baseline** | **MFI peak** | **Median MFI in HC** | **Median MFI in all pts pre-ICI** |
| --- | --- | --- | --- | --- | --- | --- |
| **SCCHN** | Myocarditis | IgG anti-cardiac myosin (MYH6)^1^ | 7200 | 12000 (week 4) | 1441 | 2764 |
| **SCCHN** | Hepatitis | IgG-CENP-B^2^ | 10900 | 17300 (week 14) | 200 | 240 |
| **SCCHN** | Skin rash | IgG Anti-HSP27^3^ | 4703 | 6481 (week 6) | 98 | 153 |
| **Melanoma** | Colitis | IgG anti-Desmin^4^ | 3389 | 3389 (baseline) | -84 | -14.5 |
| **Melanoma** | Colitis, Pneumonitis and Skin Rash | IgG anti-HSP47^5^ | 5946 | 5946 (baseline) | -10 | 61 |

1. Nussinovitch U, Shoenfeld Y. The diagnostic and clinical significance of anti-muscarinic receptor autoantibodies. *Clin Rev Allergy Immunol* 2012;42(3):298-308. doi: 10.1007/s12016-010-8235-x [published Online First: 2011/01/06]

2. Parveen S, Morshed SA, Nishioka M. High prevalence of antibodies to recombinant CENP-B in primary biliary cirrhosis: nuclear immunofluorescence patterns and ELISA reactivities. *J Gastroenterol Hepatol* 1995;10(4):438-45. doi: 10.1111/j.1440-1746.1995.tb01597.x [published Online First: 1995/07/01]

3. Yazdanpanah MJ, Taji AR, Javidi Z, et al. High levels of anti-heat shock protein 27 antibody in pemphigus vulgaris. *Ann Dermatol* 2013;25(2):266-8. doi: 10.5021/ad.2013.25.2.266 [published Online First: 2013/05/30]

4. Mayet WJ, Press AG, Hermann E, et al. Antibodies to cytoskeletal proteins in patients with Crohn's disease. *Eur J Clin Invest* 1990;20(5):516-24. doi: 10.1111/j.1365-2362.1990.tb01895.x [published Online First: 1990/10/01]

5. Morito D, Nagata K. ER Stress Proteins in Autoimmune and Inflammatory Diseases. *Front Immunol* 2012;3:48. doi: 10.3389/fimmu.2012.00048 [published Online First: 2012/05/09]
