# Supplementary figures and images for: Autoimmune PaneLs as PrEdictors of Toxicity in Patients TReated with Immune Checkpoint InhibiTors (ALERT)
Source: J Exp Clin Cancer Res. 2023 Oct 21;42:276. doi: 10.1186/s13046-023-02851-6 (PMC10589949; doi:10.1186/s13046-023-02851-6)

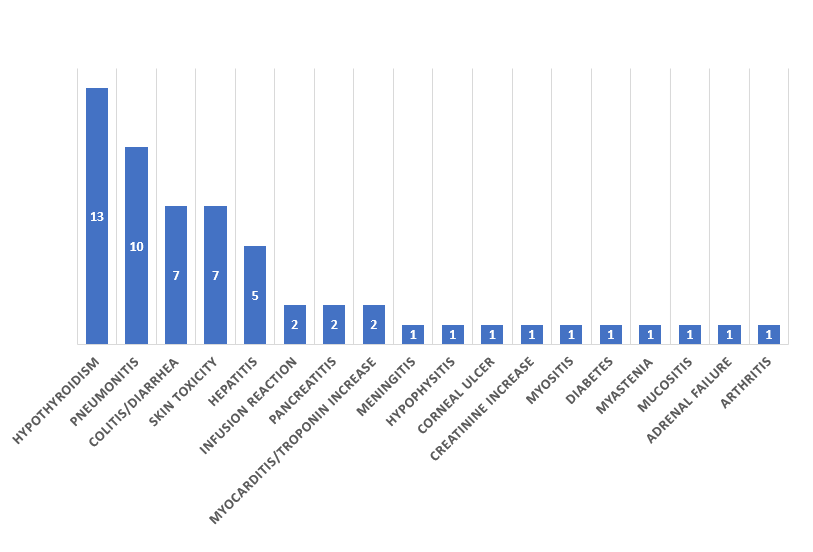


**Supplementary Figure 1:** frequencies of different irAEs G≥2.

Supplement: Supplementary file 8 — Additional file 8: Supplementary Fig. 1. Frequencies of different irAEs G≥2. [file 13046_2023_2851_MOESM8_ESM.docx]
