# Supplementary material for: Autoimmune PaneLs as PrEdictors of Toxicity in Patients TReated with Immune Checkpoint InhibiTors (ALERT)
Source: J Exp Clin Cancer Res. 2023 Oct 21;42:276. doi: 10.1186/s13046-023-02851-6 (PMC10589949; doi:10.1186/s13046-023-02851-6)
Supplement: Supplementary file 9 — Additional file 9: Supplementary Fig. 2. Changes in IgM and IgG levels in 61 patients without irAEs from baseline (pre-ICI collection) to the first collection after ICI administration. [file 13046_2023_2851_MOESM9_ESM.docx]

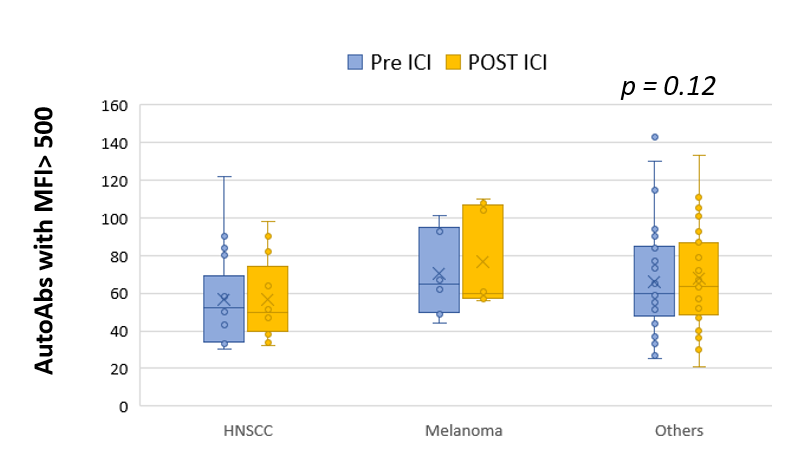


**Supplementary Figure 2. Changes in IgM and IgG levels in 61 patients without irAEs from baseline (pre-ICI collection) to the first collection after ICI administration.** The difference in the number of IgM and IgG with MFI> 500 is reported for different group of patients divided based on tumor types.
