# Supplementary material for: Autoimmune PaneLs as PrEdictors of Toxicity in Patients TReated with Immune Checkpoint InhibiTors (ALERT)
Source: J Exp Clin Cancer Res. 2023 Oct 21;42:276. doi: 10.1186/s13046-023-02851-6 (PMC10589949; doi:10.1186/s13046-023-02851-6)
Supplement: Supplementary file 10 — Additional file 10: Supplementary Fig. 3. Dynamic changes of autoAbs in patients with and without irAEs G≥2. [file 13046_2023_2851_MOESM10_ESM.docx]

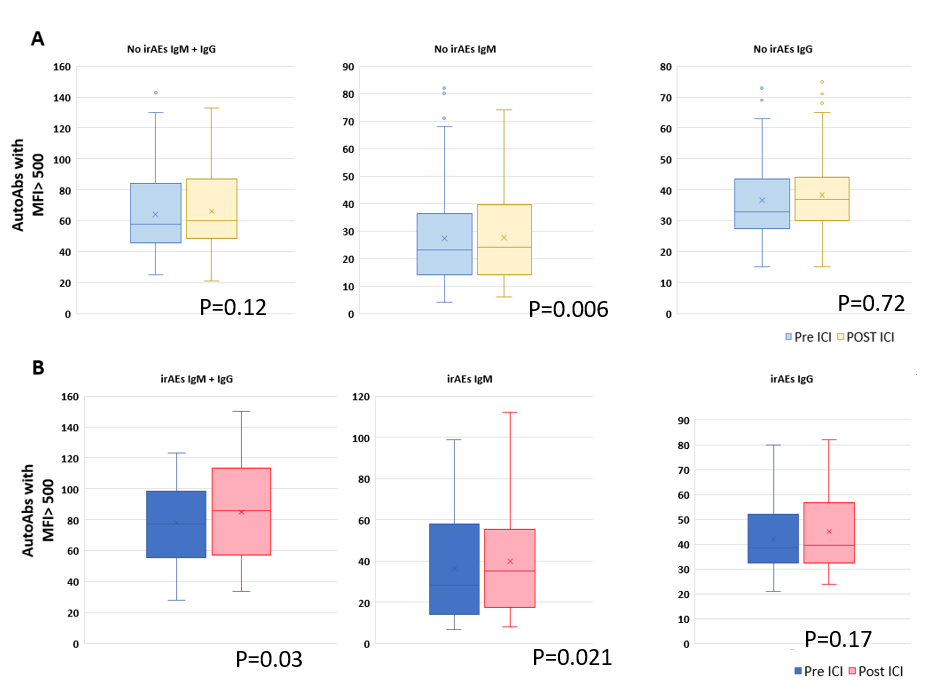


**Supplementary Figure 3. Dynamic changes of autoAbs in patients with and without irAEs G≥2.** A) Differences in the number of IgM and IgG with MFI>500 from baseline (pre-ICI collection) to the first post ICI collection in 61 patients who did not develop irAEs G≥2.B) Differences in the number of IgM and IgG with MFI>500 from baseline to the time or irAEs in 24 patients with irAEs G≥2.
