# Supplementary material for: Autoimmune PaneLs as PrEdictors of Toxicity in Patients TReated with Immune Checkpoint InhibiTors (ALERT)
Source: J Exp Clin Cancer Res. 2023 Oct 21;42:276. doi: 10.1186/s13046-023-02851-6 (PMC10589949; doi:10.1186/s13046-023-02851-6)
Supplement: Supplementary file 11 — Additional file 11: Supplementary Fig. 4. Comparison of the level of IgM and IgG with MFI> 500 at the time of irAEs and post steroids administration in 9 patients. [file 13046_2023_2851_MOESM11_ESM.docx]

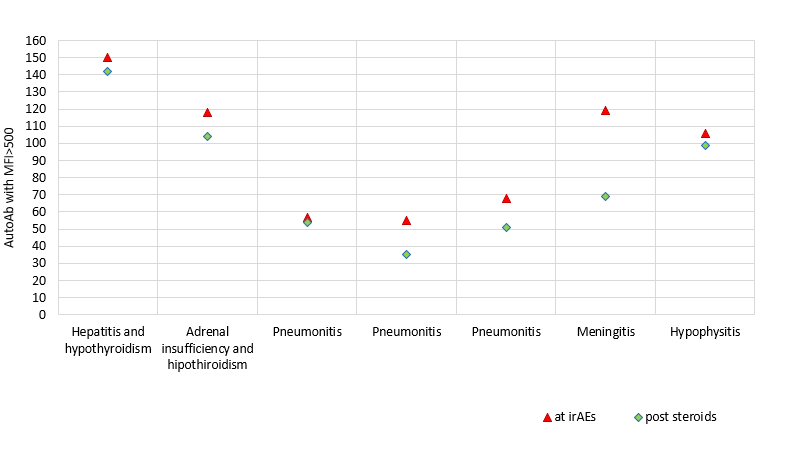


**Supplementary Figure 4. Comparison of the level of IgM and IgG with MFI> 500 at the time of irAEs and post steroids administration in 9 patients.** The number of elevated autoAbs at the time of irAEs is represented as a red triangle (one for each patient) while the number of elevate autoAbs after steroids administration is represented as a green rhombus. For each patient is reported the type of irAE developed close to the plasma collection.
