# Supplementary material for: Autoimmune PaneLs as PrEdictors of Toxicity in Patients TReated with Immune Checkpoint InhibiTors (ALERT)
Source: J Exp Clin Cancer Res. 2023 Oct 21;42:276. doi: 10.1186/s13046-023-02851-6 (PMC10589949; doi:10.1186/s13046-023-02851-6)
Supplement: Supplementary file 12 — Additional file 12: Supplementary Fig. 5. Comparison of AutoAbs levels measured with ELISA in patients with and without irAEs G≥2 and dynamic changes in patients who developed irAEs G>2. [file 13046_2023_2851_MOESM12_ESM.docx]

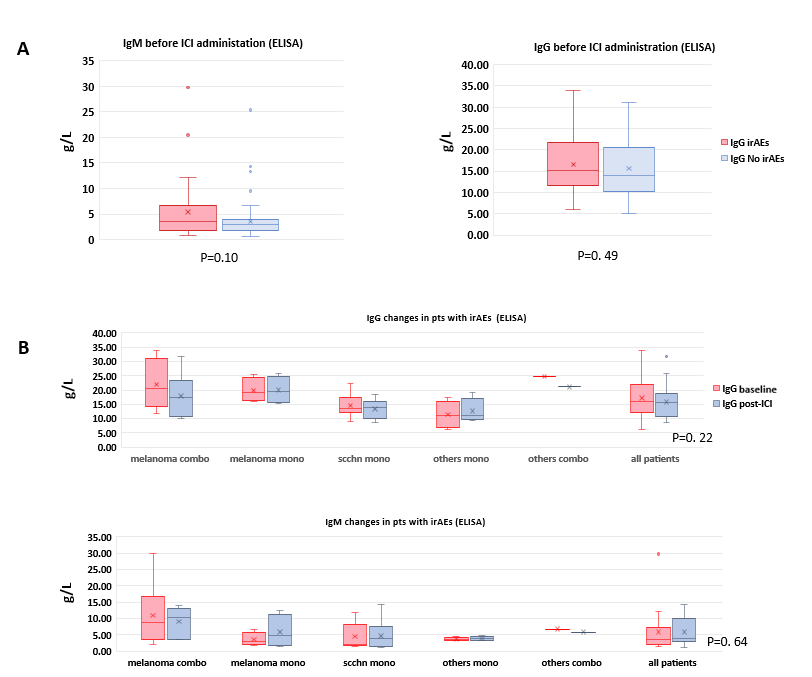


**Supplementary Figure 5. Comparison of AutoAbs levels measured with ELISA in patients with and without irAEs G≥2 and dynamic changes in patients who developed irAEs G>2**. A) Difference in the level of IgM and IgG measured with ELISA at baseline (before ICI) in patients with and without irAEs G≥2. B) Dynamic changes in the level of IgG and IgM measured with ELISA in 24 patients who developed irAEs G≥2 from baseline (before ICI administration) to the time of irAEs.
